# Identification of a Family of Glycoside Derivatives Biologically Active against *Acinetobacter baumannii* and Other MDR Bacteria Using a QSPR Model

**DOI:** 10.3390/ph16020250

**Published:** 2023-02-07

**Authors:** Francisco José Palacios-Can, Jesús Silva-Sánchez, Ismael León-Rivera, Hugo Tlahuext, Nina Pastor, Rodrigo Said Razo-Hernández

**Affiliations:** 1 Centro de Investigación en Dinámica Celular (CIDC), Universidad Autónoma del Estado de Morelos, Av. Universidad 1001, Col. Chamilpa, Cuernavaca 62209, Morelos, Mexico; 2 Centro de Investigaciones Químicas (CIQ), Universidad Autónoma del Estado de Morelos, Av. Universidad 1001, Col. Chamilpa, Cuernavaca 62209, Morelos, Mexico; 3 Centro de Investigación sobre Enfermedades Infecciosas, Instituto Nacional de Salud Pública (INSP), Av. Universidad 655, Col. Sta. Ma. Ahuacatitlan, Cuernavaca 62100, Morelos, Mexico

**Keywords:** QSPR model, antibacterials, *Acinetobacter baumannii*, natural products, virtual screening

## Abstract

As the rate of discovery of new antibacterial compounds for multidrug-resistant bacteria is declining, there is an urge for the search for molecules that could revert this tendency. *Acinetobacter baumannii* has emerged as a highly virulent Gram-negative bacterium that has acquired multiple resistance mechanisms against antibiotics and is considered of critical priority. In this work, we developed a quantitative structure-property relationship (QSPR) model with 592 compounds for the identification of structural parameters related to their property as antibacterial agents against *A. baumannii*. QSPR mathematical validation ($R^{2}$ = 70.27, $R^{N}\;$= −0.008, $a\left(R^{2}\right)\;$= 0.014, and δK = 0.021) and its prediction ability ($Q^{2}$*_LMO_*= 67.89, $Q^{2}$*_EXT_* = 67.75, $a\left(Q^{2}\right)$ = −0.068, δQ = 0.0, $\bar{r_{m}{}^{2}}$ = 0.229, and $\Delta r_{m}{}^{2}$ = 0.522) were obtained with different statistical parameters; additional validation was done using three sets of external molecules ($R^{2}$ = 72.89, 71.64 and 71.56). We used the QSPR model to perform a virtual screening on the BIOFACQUIM natural product database. From this screening, our model showed that molecules **32** to **35** and **54** to **68**, isolated from different extracts of plants of the *Ipomoea* sp., are potential antibacterials against *A. baumannii*. Furthermore, biological assays showed that molecules **56** and **60** to **64** have a wide antibacterial activity against clinically isolated strains of *A. baumannii*, as well as other multidrug-resistant bacteria, including *Staphylococcus aureus*, *Escherichia coli*, *Klebsiella pneumonia*, and *Pseudomonas aeruginosa*. Finally, we propose **60** as a potential lead compound due to its broad-spectrum activity and its structural simplicity. Therefore, our QSPR model can be used as a tool for the investigation and search for new antibacterial compounds against *A. baumannii*.

## 1. Introduction

Opportunistic infectious diseases caused by multidrug-resistant bacteria represent a world-concerning health problem that is growing at an accelerated rate. Despite the immense quantity of literature and efforts sponsored by health committees, academia, and other non-governmental organizations on antibiotic resistance [1,2,3], there is still a lack of real education campaigns to promote the correct use of antibiotics. In accordance with recent reports, more than 2.8 million antibiotic-resistant infections occur in the U.S. alone, with over 35,000 deaths as a result [3,4]. It is estimated that by 2050 a stunning 10 million deaths will be caused solely by antibiotic-resistant bacteria [5]. In Mexico, the number of deaths caused by septicemia in hospitals has been increasing in recent years, and since 2019, it has been among the 15 main causes of death [6,7,8].

From the twelve bacteria listed on the website [9] of the World Health Organization (WHO), *Acinetobacter baumannii*, *Pseudomonas aeruginosa*, and several *Enterobacteriaceae* are considered of “critical” urgency. *A. baumannii* is an opportunist Gram-negative (GN) pathogen that has gained notorious attention because of its high virulence, its multiple resistance mechanisms against antibiotics, and its great capacity for adaptation to different environments [10,11,12,13]. Its incidence has been mainly related to pneumonia (associated with the use of ventilators), septicemia (due to contamination of central and peripheral airways), and infections at the site of the injuries [14,15].

As companies have dropped out of the research and development (R&D) of new antibacterial drugs and fewer molecules have been approved by the FDA [16,17,18,19,20], the quest for novel potential candidates has decreased. Natural products (NPs) are a promising alternative to the use of traditional drugs because of their vast scaffold diversity and structural complexity, offering advantages and challenges in the drug discovery process [21]. These properties can be beneficial when compared to typical synthetic small-molecule compounds, for example, high molecular mass [22], a large number of sp^3^ carbon and oxygen atoms, which also correlate with low cLogP values (or higher hydrophilicity) [23,24,25,26], and greater rigidity [27]. Furthermore, small structural modifications to these scaffolds often lead to an improvement of the biological activity, for example, as in the case of camptothecin semisynthetic analogs topotecan and belotecan. Nonetheless, identifying bioactive compounds of interest is challenging and often takes additional time for isolation, complete characterization, and, if afforded, full synthesis [28,29]. Several analytical techniques have proven to be of relevance for this task, for example, the use of computational resources, which has reduced the amount of time and optimization of drug candidates. Quantitative Structure-Activity/Property relationships (QSAR/QSPR) have allowed the search and optimization of better bioactive molecules by determining which physicochemical and structural features (molecular descriptors) are key points for biological activity [30].

Virtual screening (VS) comprises the use of computational tools to search and analyze large databases of small molecules to identify potential bioactive compounds. VS can be divided into two major categories depending on the type of information available: ligand-based virtual screening (LBVS) and structure-based virtual screening (SBVS), both of which have been reviewed elsewhere [31,32,33]. Nevertheless, many other types of techniques have been developed to improve the accuracy of activity prediction. In this sense, the use of QSAR/QSPR as an approach for the virtual screening of large libraries of small compounds has proven to accelerate the rate of the discovery process by reducing the number of potential candidates. When comparing the hit rates of techniques like High-throughput screening (HTS) with the QSAR/QSPR-based virtual screening, it is seen that the hit rate of HTS ranges between 0.01% and 0.1%, while for the latter, it spans between 1% and 40%. [34] This has found application in the search for new antimalarial [35], anti-schistosomiasis [36], anti-tuberculosis [37], and antiviral [38,39] drugs, for which several compounds proved to be active. The usefulness of QSAR/QSPR models arises from the data used for the generation of the models, which is reflected within its applicability domain. In this sense, having a vast amount of structural information allows for a greater degree of confidence in the prediction data for virtual screening. Otherwise, data extrapolation may lead to false positives.

Due to the high resistance to different antibiotic treatments caused by *A. baumannii*, worldwide research groups have carried out important efforts in the search for compounds against this pathogen. Most of them have carried out QSAR-type studies to determine their biological properties based on the molecular structure. However, a problem regarding these QSAR models is the use of small sets of compounds, mainly those synthesized and tested in the same work with minor chemical changes at the core structure. Furthermore, small datasets considering molecules acting against multiple pathogens have the disadvantage that it is necessary to seek/use as many models as possible to determine and predict the antibacterial activity of these sets of compounds. Prado-Prado et al. developed a QSAR analysis by introducing entropy-like molecular descriptors for their models to predict the antibacterial activity of several drugs against different strains of bacteria [40]. Semenyuta and collaborators established several QSAR models for the activity of imidazolium ionic liquids with the use of neural networks and random-forest regressions [41], allowing them to use multiple molecular descriptors to correlate the structure with the bioactivity of these new compounds towards *A. baumannii*. Nonetheless, a main drawback of these QSAR analyses is the use of complex molecular descriptors that are often difficult to interpret and handle, limiting their applicability and simplicity.

One important aspect of drug design relies on the pharmacokinetics profile of molecular candidates. Indeed, a major problem at the early stages of clinical phases is due to poor pharmacokinetics of compounds, especially regarding absorption and distribution in the organism. Furthermore, when targeting microorganisms like bacteria, it is important to consider another biological barrier, the cell wall, which most molecules need to pass through to exert their bioactivity. Considering the vast amount of chemical structures for compounds that present activity against *A. baumannii*, it is possible to design a structure-property relationship model for the prediction of molecules that could have the possibility of harming the bacterium without considering a specific biological target, but that exert antibacterial activity.

In the present work, we have developed a QSPR model of active compounds (from synthetic to NPs) against multidrug resistance (MDR) *A. baumannii* by means of the genetic algorithms (GA) technique, using molecular 0D, 1D, and 2D-descriptors. The QSPR model was employed to identify structural features of the bioactivity compounds within the dataset that can be associated with their pharmacokinetic aspects (absorption and distribution). Therefore, one of the objectives of our QSPR model is to predict the entrance of the compounds into *A. baumannii* [42,43,44]. Then, our QSPR model was used to identify potential antibacterial candidates from an NP database. Furthermore, we obtained and carried out the biological evaluation of these candidates, corroborating the prediction of our QSPR model.

## 2. Results and Discussion

### 2.1. QSPR Model Validation

As a first approach, regression models were built using GA to select the most appropriate descriptors. After the selection of the descriptors, multiple linear regression analysis was performed to generate suitable models that could allow us to categorize the biological activity of the dataset. The best QSPR model for antibacterial activity against *A. baumannii* consists of fifteen descriptors as follows:

*p*MIC = (0.001 ± 0.000)**D/Dr06** + (–0.438 ± 0.004)**GATS6m** + (0.529 ± 0.004)**nArCOOH** + (1.249 ± 0.005)**nRCONH2** + (0.334 ± 0.001)**nROR** + (–0.429 ± 0.006)**nImidazoles** + (0.115 ± 0.000)**nHDon** + (–0.204 ± 0.001)**nHBonds** + (1.257 ± 0.005)**C018** + (0.476 ± 0.001)**C029** + (1.149 ± 0.004)**C032** + (–0.105 ± 0.000)**H051** + (–0.186 ± 0.001)**N075** + (–0.555 ± 0.001)**N079** + (0.025 ± 0.000)**TI2** + 4.292( ±0.005) (1)

$$\begin{array}{c} R^{2}=70.278\;(\pm 0.907);\;{R^{2}}_{\mathrm{ADJ}}=69.162\;(\pm 0.973);\;a\left(R^{2}\right)=0.014\;(\pm 0.000);\\ s=0.462\;(\pm 0.000);\;F=62.978\;(\pm 8.418);\;{Q^{2}}_{\mathrm{LMO}}=67.886\;(\pm 1.043);\;\\ {Q^{2}}_{\mathrm{BOOT}}=66.882\;(\pm 1.104);\;{Q^{2}}_{\mathrm{EXT}}=67.747\;(\pm 5.414);\;a\left(Q^{2}\right)=-0.068\;(\pm 0.000);\;\\ \delta K=0.021\;(0.000);\;\delta Q=0.000\;(-0.005);\;R^{P}=0.015\;(0.100);\;R^{N}=-0.008\;(-0.054) \end{array}$$

All statistical parameters were obtained as their average values (see Table S3), for example, the square correlation coefficient ($R^{2}$) of 70.278 (±0.907), and the $R^{2}$*_ADJ_* of 69.162 (±0.973). The Fischer F and the standard deviation (s) are 62.978 (±8.418) and 0.462 (±0.000), respectively, indicating that our model is acceptable. Moreover, redundancy and overfitting rules were checked to determine the nature of the descriptors used in the model. In this sense, the overfitting rule, $R^{N}$ = −0.008 (−0.054), was complied with fairly while the redundancy rule, $R^{P}$ = 0.015 (0.100), indicating that some descriptors, nHDon and nHBonds, are correlated to the dependent variable. However, these descriptors cannot be removed as they are important for the correct description of our regression model. Furthermore, the prediction ability of the model was validated by the leave-many-out cross-validation, $Q^{2}$*_LMO_* = 67.886 (±1.043), a value indicating that the regression model has good predictive power. The robustness parameter as indicated by the high value of $Q^{2}$*_BOOT_* = 66.882 (±1.104) based on bootstrapping, which was repeated 5000 times.

**Figure 1:**
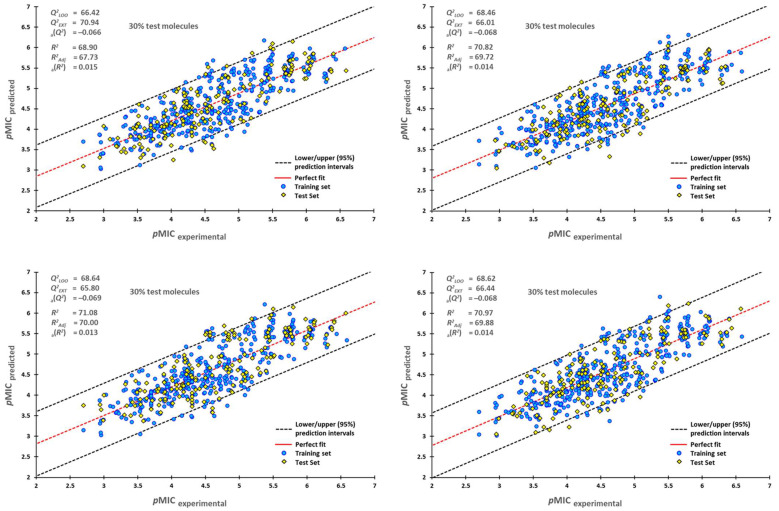
Scatterplots of predicted *p*MIC against experimental *p*MIC values. Blue dots represent molecules of the training set (70%), and yellow diamonds depict molecules used for the test set (30%). For each plot, the percentage of molecules used in the training and test datasets were randomly chosen.

External validation was essential as a high $Q^{2}$*_LMO_* only indicates a good internal validation, but it does not show a high prediction capability of the created model. Therefore, for the external validation procedure, 70% of all the molecules in the dataset were randomly selected for the training process, and the remaining 30% were used as the test set. This process was repeated six times; their plots are shown in Figure 1, with their upper/lower confidence intervals at a 95% confidence level. The Y-scrambling test was used on the training-test set, giving the new values of $a\left(R^{2}\right)$ = 0.014 (±0.000) and $a\left(Q^{2}\right)$ = −0.068 (±0.000). These new values were lower than the original ones, confirming that our model is reliable.

With the same purpose, the Asymptotic $Q^{2}$ rule, δQ = 0.000 (−0.005), was employed. Therefore, the model in (1) passed all the statistical tests proposed by Roy et al. [45,46,47], as an average value derived from ten experiments shows: (a) $Q^{2}=$ 67.88 (±1.043); (b) $r^{2}=$ 0.679 (±0.000); (c) $\left(r^{2}-r_{0}{}^{2}\right)/r^{2}=$ 0.001 (±0.000); (d) k= 0.999 (±0.000) (or $k^{\prime }=$ 0.991 (±0.000)); (e) $\left|r_{0}{}^{2}-{r^{\prime }}_{0}{}^{2}\right|=$ 0.127 (±0.000). For an acceptable prediction, the value of $\Delta r_{m}{}^{2}$ should preferably be less than 0.2, while $\bar{r_{m}{}^{2}}$ should be greater than 0.5. In our model, $\Delta r_{m}{}^{2}$ presents a value of 0.229 (±0.000), while $\bar{r_{m}{}^{2}}$ has an average value of 0.552 (±0.000). A complete list of each evaluation can be seen in Table S4.

**Figure 2:**
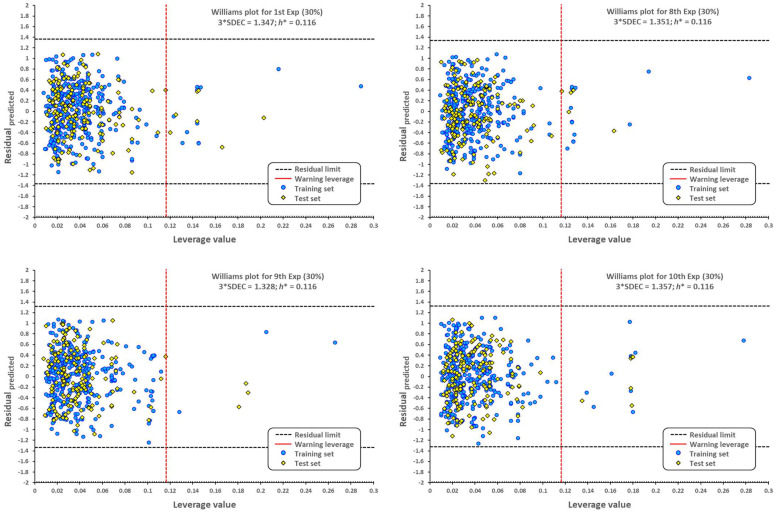
Williams plots for molecules with antibacterial activity against *A. baumannii*. The dotted vertical line in red indicates the warning leverage limit ($h^{*}=3p/n$, where *n* is the number of molecules and *p* is the number of descriptors in the model plus one). The upper/lower dotted horizontal lines in black represent the boundaries for which the triple of the standard deviation (3 × SDEC) value is used.

The applicability domain is graphically depicted by the Williams plot in Figure 2. For each compound, the leverage values can be calculated, and by plotting these values against the standardized residuals, it is possible to establish the applicability domain of the developed model [48]. This allows the detection of molecules that our model cannot predict adequately, thus considered as outliers [49,50], molecules with distinctive structures (high leverage outliers, $h>h^{*}$), or those associated with the response (predicted residuals > 3 × SDEC). All compounds that are outside the limits established by the leverage warning and three times the standard deviation in error calculation are outliers.

**Figure 3:**
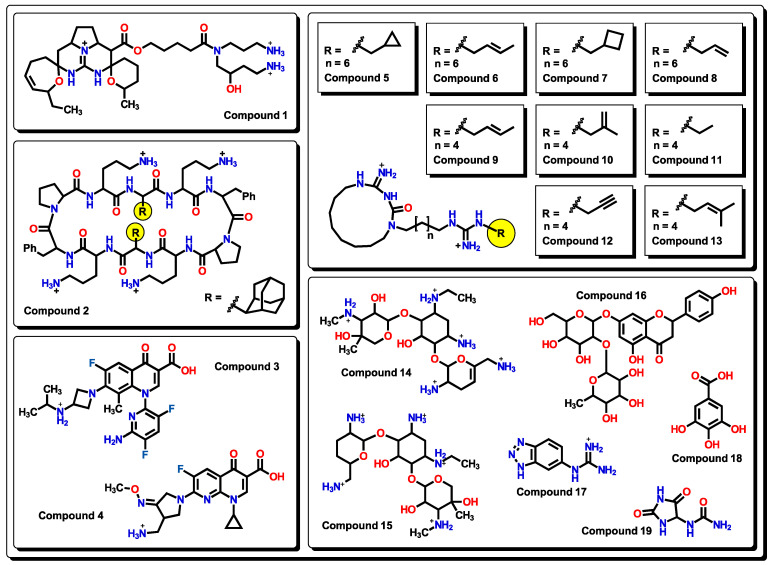
Chemical outliers obtained from the analysis of the Williams plots.

As seen from the Williams plot, outliers are correlated to the structure of molecules. Due to the relatively wide variety of molecular structures used in our model, detected outliers both from the training and test sets are very different (Figure 3).

In compound **1**, although it shares a similar structure with those of the Batzelladine alkaloids’ family used in this model [51], the two cyclic ether-like motifs at the central positively charged nitrogen core, as well as two pendant primary aliphatic charged amine arms, are distinctively different from the rest of the analyzed molecules. Compounds **1**, **2**, and **5** to **13**, **14**, **15**, and **17** are considered outliers because of the many positively charged nitrogen atoms present in the molecules. Two fluoroquinolone derivatives are present as outliers: compound **3** possesses a 3,5-difluoro-substituted pyridine instead of the common cyclopropyl or ethyl groups at nitrogen, while compound **4** has a pyridine-type structure at the core as in nalidixic acid. These two features are unique among the set of fluoroquinolones used in our model. Compounds **14** and **15**, being both aminoglycosides, are seen as outliers from our model as it is suggested that amino groups are responsible for this distinction. Compound **16** is a flavanone-7-O-glycoside. Although there are many flavanones in the dataset, none of them present a disaccharide (or any mono- or polysaccharide), which makes **16** unique. On the other hand, many examples of substituted triazoles are seen in our model, but molecule **17** has a benzotriazole that is unique; thus, it is considered an outlier. Even though there are many compounds with aromatic alcohols, **18** (gallic acid) possesses a benzenetriol motif that is not encountered in any other molecule. Structure **19** has the hydantoin functional group, which is unique among the set of active molecules against *A. baumannii*. 

To test the reliability of our QSPR model, molecules that were not introduced in our initial dataset were employed as an external validation set to obtain their predicted *p*MIC values. Three sets of compounds were used as follows: (a) the first set of molecules reported by Matsingos et al. [52]; (b) compounds reported by Singh [53], Wang [54], and Zhou [55] as the second set, and finally, (c) chemical structures described by Lyons and collaborators [56]. For the three sets of data, there is a good correlation between experimental and predicted *p*MIC values with $R^{2}$ values of 75.54, 71.64, and 71.56, respectively. 

On the other hand, compounds that exhibit, for example, many positively charged nitrogen atoms like those reported by Vereshchagin and co-workers [57] or molecules with unusual functional groups like those reported by Siricilla [58] are not well predicted by our model in accordance with the results of the outliers analyzed previously. This fact can be explained by the limited number of similar molecules (charge and scaffold related) used in the generation of the QSPR model. Nevertheless, this type of molecules is very interesting and important for the development of antibacterial compounds since many of them use facilitated diffusion transport related to amino acids, like Lys, His, and Arg.

**Figure 4:**
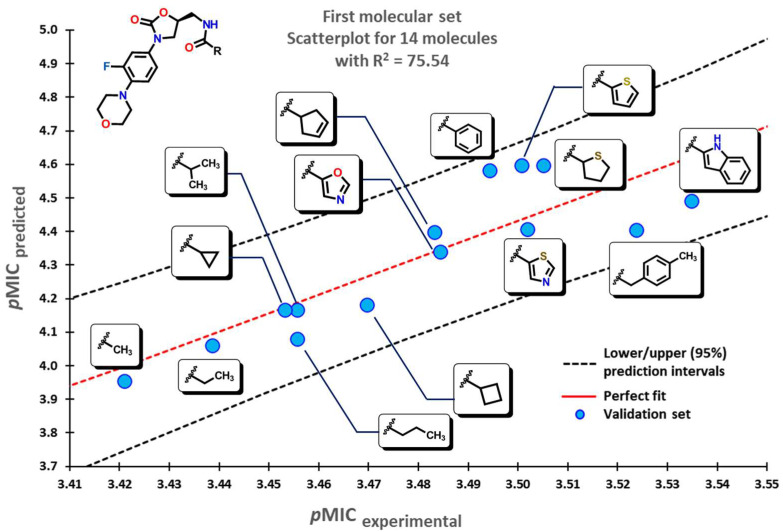
Scatterplot for molecular data set of linezolid analogs. Selected molecules are displayed within the plot showing the change of substituent.

Our model applied to the first set of linezolid analogs with different C5-acylamino substituents gives an insight into their structural features. An increase in the *p*MIC values is seen when moving from small-chain alkyl groups (Me, Et, or nPr) to small-branched or cyclic alkyls motifs (*i*Pr, cyclopropyl, cyclobutyl) and finally to aromatic substituents. This increase is shown in Figure 4.

**Figure 5:**
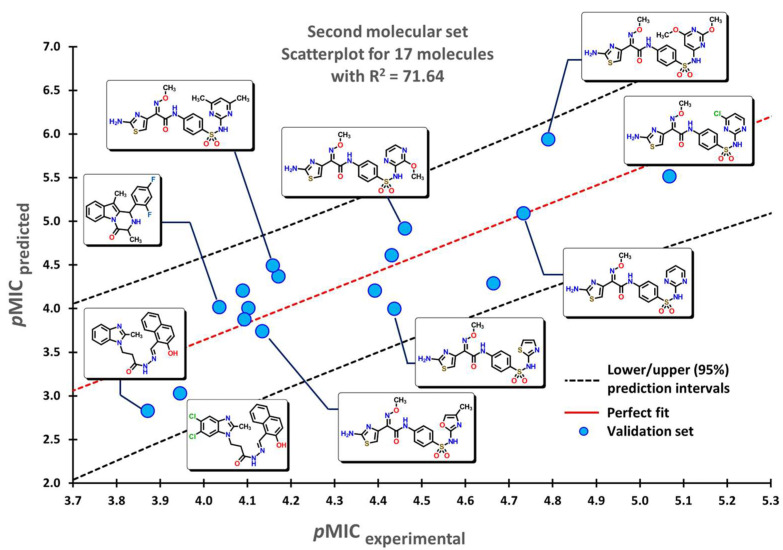
Scatterplots for the molecular data set were used for the validation of the QSPR model. Selected molecules are displayed within the plots.

**Figure 6:**
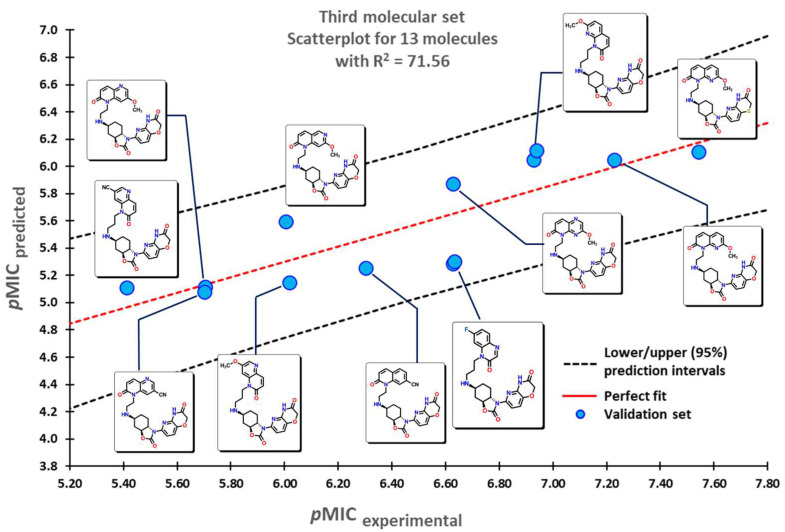
Scatterplot for oxazolidinone derivatives used for the validation of the QSPR model. Selected molecules are displayed within the plots.

The second set of compounds comprises three different groups of molecules for which our model classifies first the divin derivatives, moving into pyrazinoindole analogs, and finally with the subset of 2-aminothiazole sulfanilamide oximes, as seen in Figure 5. The last set of compounds comprises several oxazolidinone derivatives in Figure 6. The first molecules are classified in accordance with the structure of the 1,5-naphthyridin-2(1*H*)-one, while the last ones have a 1,8-naphthyridin-2(1*H*)-one. Molecules at the center possess the nitrogen atom at different positions of the quinolin-2(1*H*)-one core.

### 2.2. QSPR Interpretation

The understanding of the descriptors presented by the QSPR model allows us to gain some insights into the chemical features of the molecules used in the model that are relevant for their antibacterial activity towards *A. baumannii*. Equation (1) displays two topological descriptors (D/Dr06 and TI2), one 2D-autocorrelation (GATS6m), six functional group counts (nArCOOH, nRCONH2, nROR, nImidazoles, nHDon, and nHBonds), and six atom-centered fragments (C-018, C-029, C-032, H-051, N-075, and N-079), all of them being 2D-dimensional descriptors.

**Figure 7:**
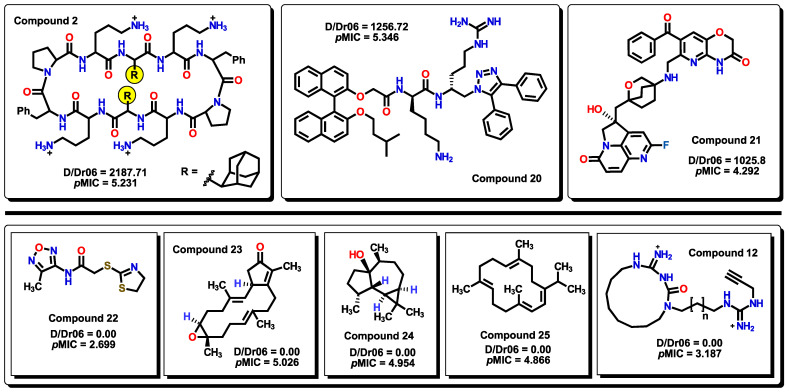
On the top row, the highest D/Dr06 values are displayed for compounds. Below, molecules that do not have any 6-membered rings in their structures display a zero value of the descriptor.

The first descriptor in the model is D/Dr06, a topological descriptor. Distance/detour ring indices (D/Dr*k*) are calculated by summing up distance/detour quotient matrix row sums of vertices belonging to single rings in the molecule. These descriptors can be considered special substructure descriptors reflecting local geometrical environments in complex cyclic systems [59]. D/Dr06 displays a positive coefficient value, indicating that the presence of this descriptor enhances the activity of the molecule. This descriptor appears when a 6-membered cyclic structure is present in the molecule. From the set of compounds, most of the cyclic structures belong to benzene-type rings (both carbocyclic and heterocyclic). D/Dr06 has been used in a similar way for the description of the anticancer activity of aromatic molecules [60]. The highest D/Dr06 value belongs to compound **2**, where two adamantyl moieties are present in the molecule. Values of zero correspond to molecules that do not display any 6-membered cyclic systems, such as compounds **22** to **25**, as seen in Figure 7. Furthermore, molecules that display high values of D/Dr06 also show high *p*MIC values.

The second Mohar index [61] (TI2) is calculated from the eigenvalues of the Laplacian matrix as shown:

(2) $$\mathrm{TI}2=\frac{4}{\mathrm{nSK}-\lambda_{\mathrm{nSK}-1}}$$

where the nSK is the number of non-H atoms and $\lambda_{\mathrm{nSK}-1}$ is the first non-zero eigenvalue. TI2 is a topological descriptor and belongs to the Mohar indices that are related to the solubility of compounds. In general, it is associated with size, shape, and symmetry, as well as with the branching or cyclicity of the molecule. TI2 shows a positive coefficient value, indicating that by increasing the value of the descriptor, the expected *p*MIC values will also increase. This descriptor has been used in the explanation of the activity of diaryl urea derivatives [62] and in the QSAR analysis of aminomethyl-piperidones [63].

**Figure 8:**
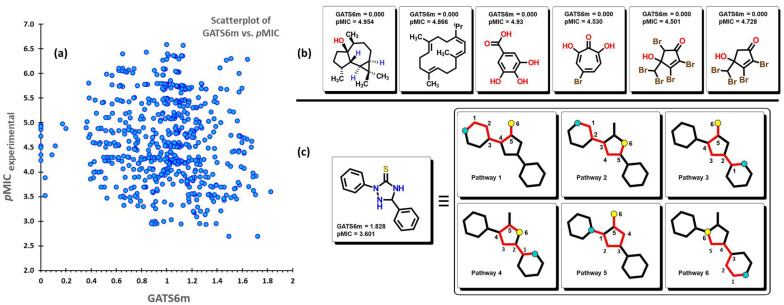
(**a**) Scatterplot of the GATS6m descriptor vs. the experimental *p*MIC value of the 592 molecules. (**b**) Molecular structures of compounds with zero value of GATS6m. (**c**) Selected pathways are used for the calculation of the descriptor.

The GATS6m [64,65] descriptor belongs to the 2D autocorrelation indices where the Geary coefficient is a distance-type function that can be any physicochemical property (*w*), calculated for each atom, such as atomic mass, polarizability, or volume, among others, and is represented by (3). By summing the products of a certain property of two atoms located at a certain distance or spatial lag (*k*), a spatial autocorrelation can be obtained.

(3) $$GATS\left(k,w\right)=\frac{\left(\frac{1}{2\Delta k}\right)\cdot \sum_{i=1}^{A}\sum_{j=1}^{A}{\left(w_{i}-w_{j}\right)}^{2}\cdot \delta (d_{ij};k)}{\left(\frac{1}{A-1}\right)\cdot \sum_{i=1}^{A}{\left(w_{i}-\bar{w}\right)}^{2}}$$

where *A* is the number of non-hydrogen atoms, $\bar{w}$ is the average of the $w_{i}$ atomic property value, $\delta (d_{ij};k)$ is the Kronecker delta, and Δk is the number of vertex pairs at a distance equal to *k*. GATS6m is the mean Geary autocorrelation of lag 6/weighted by atomic mass, which means that this descriptor considers the atomic mass of any atom in the structure at different path lengths (lag) of 6. Strong spatial autocorrelation between pairs of atoms produces low values of this index. Moreover, symmetric or low-branched structures, as well as molecules with a low number of heteroatoms (atoms besides C and H), are expected to produce low to zero values. The GATS6m descriptor displays a negative coefficient in (1), which indicates that by increasing the autocorrelation between pairs of atoms considering their atomic masses at a distance of 6 between them, the value of this descriptor will increase, causing a reduction in its *p*MIC value. As seen in Figure 8a, there is a homogeneous distribution of the data when plotting the GATS6m descriptor against the corresponding *p*MIC values. Eight molecules from the dataset have a zero value of GATS6m; their structures are displayed in Figure 8b. Furthermore, these molecules are seen to have a medium interval of *p*MIC (between 3.5 to 5) relative to their location in the scatterplot. In Figure 8c, for the molecule with the highest GATS6m value, selected pathways are shown for which the sum of their atomic masses produces the final value.

**Figure 9:**
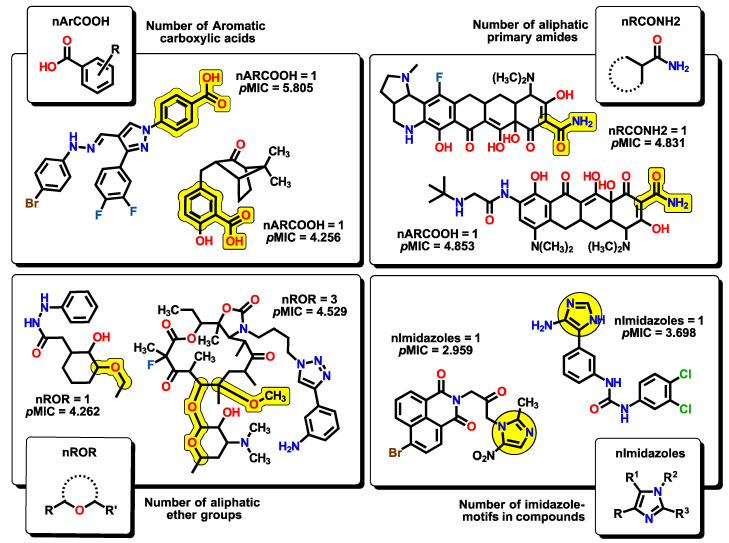
Functional-group count (FGC) descriptors with some representative molecules for each nArCOOH, nRCONH2, nROR, and nImidazoles. The corresponding functional groups are highlighted in yellow.

The next six descriptors belong to the functional-group counts (FGC), which are considered indicator variables. Their value will depend on the number of functional groups present or absent from the molecule, meaning that not all compounds will feature them. The FGC has been used to identify structural features that are important for a property of particular interest. Therefore, their presence or absence can significantly alter the predicted activity in the model. Each FGC descriptor can be easily understood in terms of the nature of functional groups. For example, nArCOOH, nRCONH2, nROR, and nImidazoles account for the number of aromatic carboxylic acids, the number of aliphatic primary amides, the number of aliphatic ethers, and the number of imidazole moieties, respectively (Figure 9).

**Figure 10:**
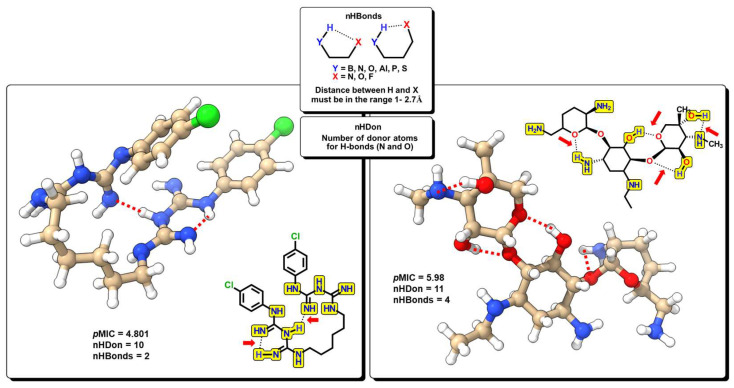
Functional-group count (FGC) descriptors with some representative molecules for nHDon and nHBonds. Highlighted in yellow are groups for which hydrogen donor atoms are counted (nHDon). Red arrows indicate the groups where intramolecular hydrogen bonds are possible (nHBonds).

The nHDon descriptor indicates the number of hydrogen donor atoms (–NH_2_ and –OH) for which the formation of hydrogen bonds is possible; in the same manner, nHBonds accounts for the number of intramolecular hydrogen bonds that are possible when there are acceptor atoms like N, O, or F, as shown in Figure 10. Intramolecular hydrogen bonds are crucial for the biological activity of many compounds. It is well established that intramolecular hydrogen bond formation can lead to temporarily closed ring systems which are more lipophilic in nature, while open forms are exposed to solvent, lending more hydrophilic character to the molecule [66]. For example, small hydrophilic molecules, such as β-lactams, use the pore-forming porins to enter cytoplasm/periplasm [67], while hydrophobic drugs like macrolides diffuse across the lipid bilayer [68]. In our model, the nHDon descriptor displays a positive value, indicating that a high number of hydrogen donor atoms (high aqueous solubility) leads to an increase in biological activity. However, as the nHBonds descriptor possesses a negative coefficient, it indicates that as the number of intramolecular hydrogen bonds increases, the biological activity will decrease, which is correlated to a more lipophilic nature of the molecules. Therefore, highly polar molecules are favored as antibacterials against *A. baumannii*, as expected for Gram-negative bacteria.

**Figure 11:**
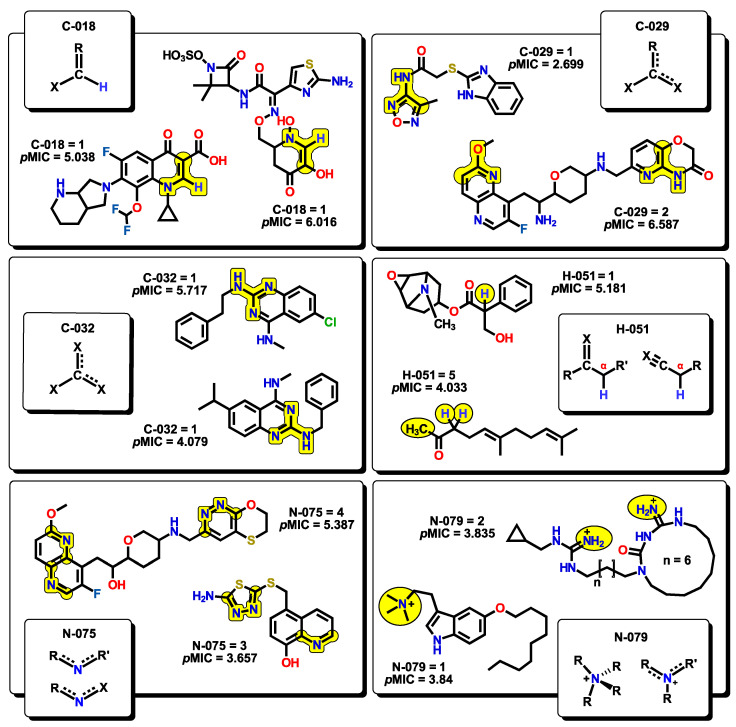
Atom-centered fragments (ACF) descriptors with representative molecules that incorporate them within their structures.

Six atom-centered fragment (ACF) descriptors are present. ACF descriptors are based on structural fragments which contain information about the central atom and their bonding neighbors [69,70,71]. Each ACF is defined by the type of bonding, as well as the number and nature of the neighbors bounded to the centered atom. For example, C018 (=CHX) corresponds to an sp^2^ C atom which is single-bonded to a hydrogen and to any electronegative atom (such as N, O, S, etc.). The C029 (R--CX--X) descriptor, for which the “--” represents an aromatic bond (e.g., benzene) or delocalized bonds (as in the N–O bond in a NO_2_ group), corresponds to a central sp^2^ C atom that is single-bonded to an electronegative X atom, and also both double-bonded to an X atom and an R group, in which their bonds are delocalized. The C032 (X--CX--X) descriptor behaves in a similar fashion to C029, but instead of an R group, it is replaced by a third X atom. This descriptor has also been used for the analysis of chemical features essential for anticoronaviral activity [72]. The H051 descriptor stands for the environment in which a hydrogen atom is bonded. It is defined as a hydrogen that is attached to an alpha-C atom; an alpha-C may be defined as a carbon connected through a single bond with –C=X (double bond), –C≡X (triple bond), or –C--X (aromatic bond), where X represents any electronegative atom, like in the case of alpha-hydrogens in carbonyl compounds. This descriptor has been used to explain the activity of a series of molecules containing nitroaromatics motifs as radiosensitizers [73]. The next two descriptors, N075 and N079, are nitrogen-based structural fragments. The first one is defined as a central sp^2^ N atom that is bonded to two R groups or to one R and X groups (R--N--R or R--N--X), like in pyridine-type motifs. This descriptor is particularly important as many molecules in our set present these kinds of motifs. The second descriptor is related to any nitrogen atom which bears a positive charge. Representative examples for each of the ACF descriptors are presented in Figure 11.

From a general view, descriptors in Equation (1) can be classified into global and indicator variables. Global terms like GATS6m and TI2 are present in the molecule and give information about the whole structure, while indicator variables only appear if the molecular structure contains the motif. Furthermore, descriptors can be associated with the steric and electronic properties of the molecule (D/Dr06, GATS6m, and nImidazoles, as well as the six-ACF descriptors), while others are more related to the solubility of compounds, like in the case of nHDon, nHBonds, TI2, as well as functional groups like nArCOOH, nRCONH2, and nROR. Electronic parameters can be associated with atom-centered fragments, which indicate the distribution of substituents around a specific atom. As many molecules include within their structure specific ACF moieties, their inclusion will lead to an increase or decrease in the predicted *p*MIC value. For example, the three ACF based on central carbon (C-018, C-029, and C-032) are positive in their signs, indicating that their presence enhances bioactivity. Furthermore, as they are carbon ACF descriptors, they can be associated with core-structure features. However, H051, N075, and N079 ACF descriptors lead to a decrease in the activity. H-051 counts hydrogen atoms that are reactive, and hence, they are prone to be abstracted by the use of bases. Nitrogen atoms like those described by the N075 descriptor are good hydrogen bond acceptors, leading to the generation of inter- and intramolecular interactions by the use of their lone pairs of electrons, which decreases the solubility of molecules, as stated by the nHBonds descriptor. On the other hand, molecules that are well-solvated in aqueous media are expected to be high in *p*MIC values.

**Figure 12:**
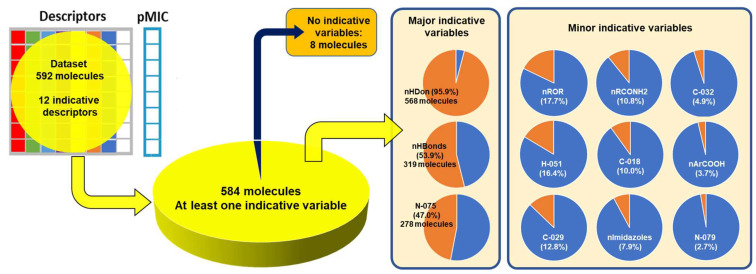
Local distribution of descriptors in molecules used in the model.

Figure 12 shows the percentage of distribution of the descriptors for molecules in the dataset. 95.9% of the molecules (568) have the nHDon functional group, and almost all other descriptors fall within this category. The second major descriptor that appears in the dataset is nHBonds, with 53.9% of the molecules (319), followed by the N075 descriptor in 278 molecules of the subset (47%). Considering the high number of bioactive compounds, which includes pyridine-fused or pyridine-containing heterocycles, as well as their tendency to participate in hydrogen bonding, the presence of the N075 descriptor in great percentage is important to account for the description of the activity of molecules [74,75].

**Figure 13:**
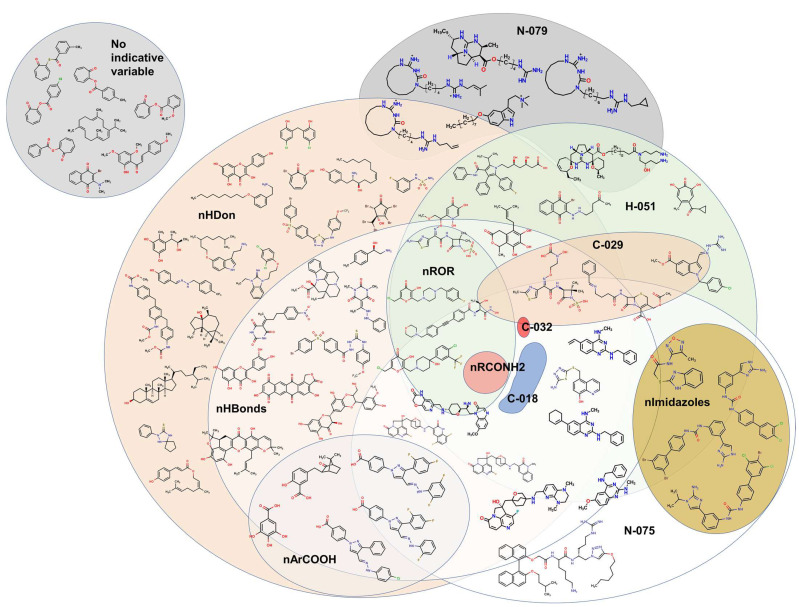
Venn diagram showing representative molecules from the dataset classified by the presence of at least one molecular descriptor and their correlations. Molecular descriptors used for the Venn diagram are nHDon, nHBonds, nArCOOH, nRCONH2, nROR, nImidazoles, C018, C029, C032, H051, N075, N079.

Some molecules are observed to be outside the boundaries of the nHDon/nHBonds descriptors, which agrees with the presence of compounds without donor groups, such as hydroxyls (–OH) or amines (–NH2), as in natural products. The rest of the molecules are located into these major categories, which can be seen adequately in the Venn diagram [76,77,78,79,80,81] in Figure 13. It is also seen that eight molecules lack the rest of the molecular descriptors used in the model. Thus, they are depicted outside the Venn diagram as a sole group.

### 2.3. Virtual Screening Using BIOFACQUIM Dataset

**Figure 14:**
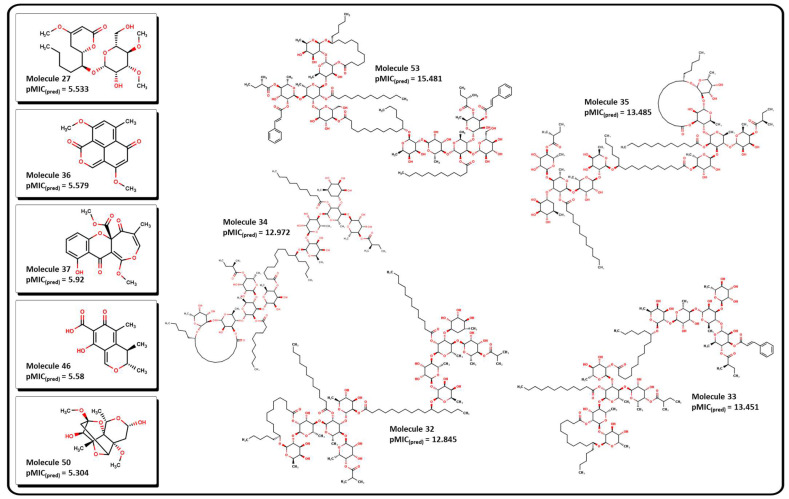
Molecular structure of selected natural products. Molecules **32** to **35** and **53** exhibits the highest predicted *p*MIC value. Molecules **27**, **36**, **37**, **46**, and **50** show low predicted values according to Equation (12).

Once we fully validated our model for antibacterial activity against *A. baumannii*, we proceeded to search for new molecular candidates in an online database of molecular compounds. NP databases are relevant sources of biologically active compounds, which often comprise complex molecular structures isolated from diverse organisms. However, because there is no globally accepted community resource for NPs, an impressive number of sites contain information for several isolated molecules, which often differ in annotation and structure [82,83,84]. BIOFACQUIM [85] is a Mexican natural product database that comprises 528 compounds isolated from many plants and other organisms across Mexico. The BIOFACQUIM database was selected for the initial evaluation since those natural products isolated from different species from Mexico would represent a reliable and affordable option for biological assays. As a second approach for tracking new molecular candidates, larger databases from different regions across a continent would be beneficial. After careful curation of the database and calculation of their descriptors (Table S5), we performed the analysis of the molecules using our QSPR model. The predicted *p*MIC values from molecules of the database range between 1.65 and 11.24. Table 1 shows these values for the most active molecules, suggested by our model, and depicted for some structures in Figure 14. As stated in Equation (1), a high value of the calculated *p*MIC implies a small concentration of the compound, which correlates to an increase in its potency. In this sense, desirable molecules should exhibit high *p*MIC values.

Table 1 shows that molecules with the highest predicted *p*MIC values are compounds **32** to **35** and **53**, which were isolated from several plants of the genus *Ipomoea* [86,87]. Their molecular structures contain several functional groups that contribute to their predicted activity. Three important features are observed: (1) all of them have a high number of pyranose-like rings, which may contribute to their hydrophilicity properties; (2) most of them contain large aliphatic side chains and/or macrocyclic lactone rings, which may contribute to their lipophilicity; (3) all of them present at least one terminal ester group which may be prone to cleavage by hydrolysis in aqueous media. Molecules **59** to **62** and **64** also exhibit terminal carboxylic acid fragments.

Analyzing these characteristics in our model, we can obtain some insights regarding the structural information that correlates to the predicted values. For example, all the molecules exhibit a great number of aliphatic ether groups and, according to our model in Equation (12), as the number of aliphatic ether motifs (nROR) increases, the greater their activity will be. This is highly correlated to a large number of donor atoms (oxygens) and, therefore, as the number of nHDon increases, so does the predicted bioactivity. Nonetheless, a great number of donor atoms also increases the possible number of intramolecular hydrogen bonds (nHBonds), which, according to our model, diminishes the predicted values. Another descriptor that appears to affect the predicted values is H-051, which implies the presence of hydrogens attached to alpha-carbon atoms, known as alpha-hydrogens (α-H). As the number of α-H increases, the bioactivity tends to decrease. In those molecules that are predicted with the highest *p*MIC values, ester and carboxylic acid groups appear in great numbers, suggesting that these kinds of functional groups are not adequate for their pharmacokinetic profile, as all of them exhibit α-H. Another feature is the presence of a high number of pyranose-like rings, which are 6-membered rings; thus, the high D/Dr06 value is displayed. Furthermore, because of their structure, these molecules are highly branched, which is seen in their high TI2 values. GATS6m is complex in nature but correlates well with the molecules under analysis. As the average number of possible 6-pathways for which heavy atoms can be included, there is a decrease in the predicted bioactivity. There are many known bioactive compounds for which their molecular masses are substantially high, for example, the macrolides and some other natural products like digitoxin [21,22,88,89]; thus, they violate one of Lipinski’s rules used for the evaluation of possible new drugs [90]. Molecules **31**, **48**, and **53** present relatively medium-high GATS6m values, hence high molecular mass; however, our model predicts elevated *p*MIC for these compounds. This can suggest that there could be a limit in the mass of the molecule and the number of oxygen atoms or any other heavy elements that will cause molecules to be less active.

### 2.4. Antibacterial Activity Evaluation

**Figure 15:**
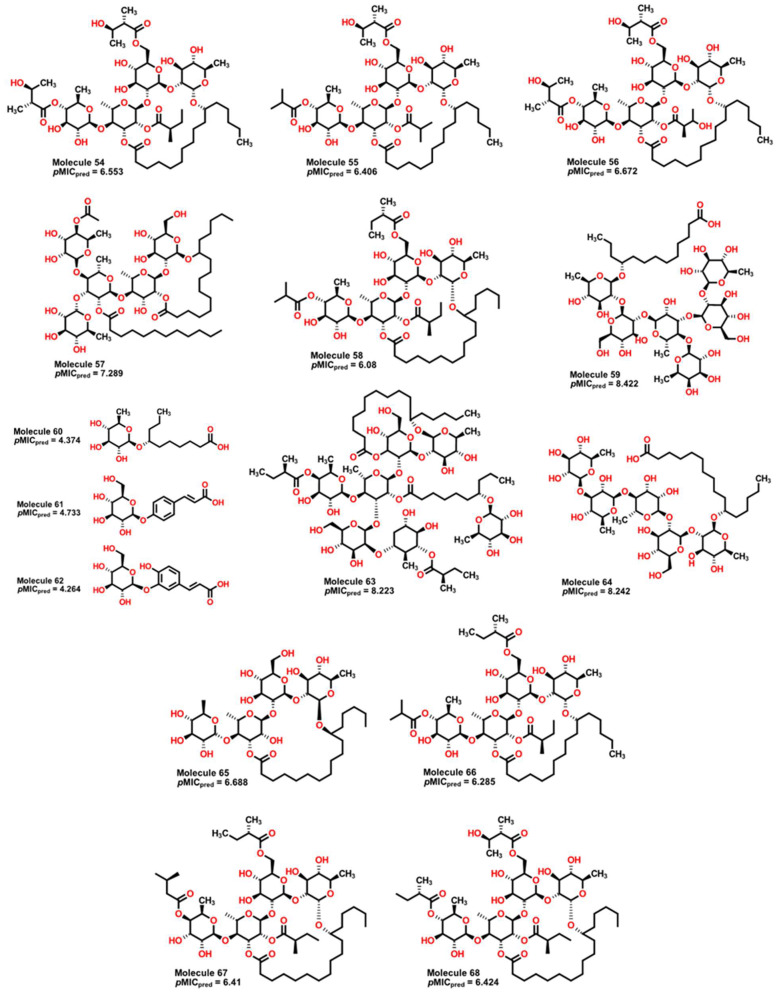
Molecular structure of natural products isolated from different species of *Ipomoea*. Molecules **59**, **63**, and **64** display high values of predicted *p*MIC, while compounds **60** to **62** show the lowest values.

Having identified molecular properties with high potential activity against *A. baumannii* from plants, we searched for similar molecules from the same genus of plants *Ipomoea*. Several isolated molecules from plants of the species *I. stans*, *I. purga*, *I*. *murucoides*, and *I. tyrianthina* [91,92,93] were subject to treatment with our model to obtain their predicted values prior to experimental work. Results are shown in Table 2, and Figure 15 depicts molecules with the highest and lowest predicted *p*MIC values.

Compounds **59**, **63**, and **64** exhibit the highest predicted values of *p*MIC of 8.422, 8.223, and 8.242. From our QSPR model, we can observe some important features which are present in these compounds. First, molecules from **54** to **68** present many aliphatic ether groups from the pyranose-type rings, consequently a great number of hydrogen donors (nHDon descriptor), which contributes to an increase in their antibacterial activity. As the pyranose-type rings are six-membered structures, the D/Dr06 descriptor also promotes a rise in the expected *p*MIC. However, because of a large number of oxygen atoms and carbonyl motifs, the nHBonds and H-051 descriptors have a considerable effect in decreasing the predicted *p*MIC. Furthermore, the value of the calculated GATS6m, compared to other molecules, implies a small negative contribution to the predicted activity, which is balanced by the contribution of the D/Dr06 descriptor. Compounds **60**, **61**, and **62** (Figure 15) are predicted to have the lowest *p*MIC values (4.781, 4.733, and 4.264, respectively). This situation is due to the presence of only one pyranose-type ring in each structure, hence, only one aliphatic ether group and a reduced number of oxygen atoms. Moreover, given their molecular structure (low symmetry), their GAST6m values are also the highest among the compounds, thus diminishing the predicted value. Consequently, it is expected that a great number of pyranose-type rings that do not form intramolecular hydrogen bonds are valuable for the antibacterial activity of these compounds.

### 2.5. Glycoside SAR Analysis

As stated before, the increasing number of multidrug-resistant bacteria represents an important risk to human health worldwide. Although *A. baumannii* represents a serious threat, the search for wide-spectrum antibiotics for the treatment of infections caused by several of the ESKAPE pathogens is crucial. To determine if the proposed molecules display antibacterial activity towards this bacterial critical group, the corresponding bioassays were tested using clinical isolates, which are metallo-β-lactamase producers and resistant to beta-lactam antibiotics (Table 3). Experiments were conducted by adding 3 μL of compound solution over each agar plate.

**Figure 16:**
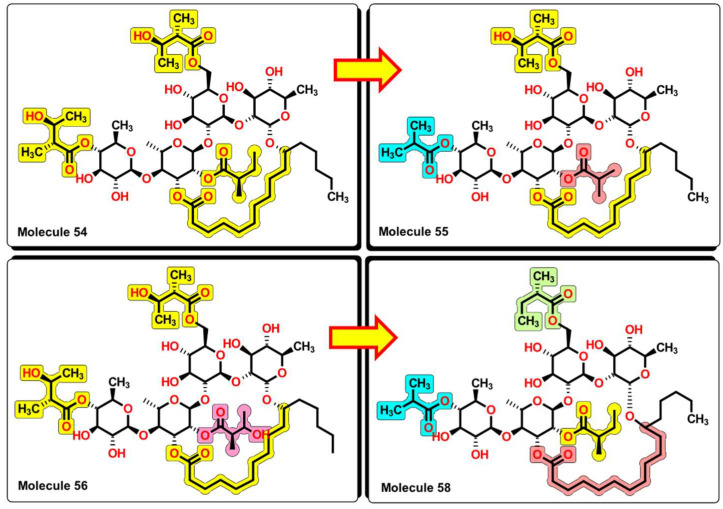
SAR analysis of compounds **54**, **55**, **56**, and **58**. Small changes in the structure expand the antibacterial activity from **54** to **56**. Removal of –OH groups and elongation of the alkyl chain in the macrocyclic ring decrease the bioactivity of the molecule.

From the results, important features arise from the molecular structures of the glycosides. First, the molecular structures of compounds **54**, **55**, **56**, and **58** contain the same tetrasaccharide core, which is connected by a macrolactone ring. From **54** to **55**, the removal of one carbon atom from central 2-methylbutyrate to 2-methylpropionate increases the activity of the glycoside, being active not only in *K. pneumonia* but also now to *P. aeruginosa* and *S. aureus*. In compound **56**, the reinsertion of the carbon atom but with the addition of a hydroxyl group at position three of the 2-methylbutyrate group reinforces the activity spectrum by being active to *A. baumannii*, as seen in Figure 16. However, the removal of the hydroxyl group of the central and outer 2-methylbutyrate groups and the addition of one carbon atom of the macrolactone ring (from ten atoms to eleven) causes molecule **58** to lose wide spectrum activity and to be only active against *K. pneumoniae*. This suggests that hydroxyl groups located in specific regions of this molecular core enhance the bioactivity of this set of glycosides.

Compounds **60** to **62** are the smallest compounds. They share in common a terminal carboxylic acid alongside a pyranose ring. Although **60** has wide antibacterial activity against multidrug-resistant bacteria, **61** and **62** only display activity against *A. baumannii*. This important loss of activity may be attributed to the removal of the aliphatic chain connecting the pyranose ring and the terminal carboxylic acid, being replaced by a more rigid phenyl core. Close inspection of compound **63** reveals the structure of compound **60** within it, forming an ester bond at the terminal carboxylic acid group. This feature could explain the retained activity against *A. baumannii*. Similar to this, molecules **54** to **56** share common structural features, like at the macrolactone ring with the same set of atoms; in addition, the lack of hydroxyl groups at the outer methylbutyrates may affect the expected activity.

**Figure 17:**
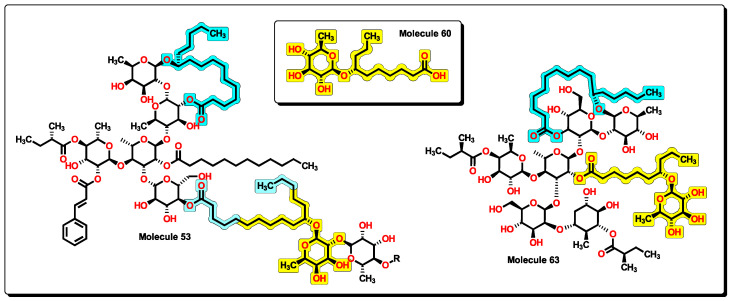
Partial chemical structure of **53** and complete molecule **63**. A chemical core of **60** is displayed within the other structures. Furthermore, the macrolactone ring is shared between compounds.

An insight into the chemical structures of **32** to **35** and **53**, the most potent molecules according to the model in Equation (12), reveals that the core of **60** is present (Figure 17). Furthermore, the macrolactone ring alongside the chiral carbon is also a common feature, with the cycle formed of ten or eleven methylene groups as in **54** to **58**. This could suggest that molecules of the BIOFACQUIM database would also exert antibacterial activity towards *A. baumannii* and other resistant bacteria.

**Figure 18:**
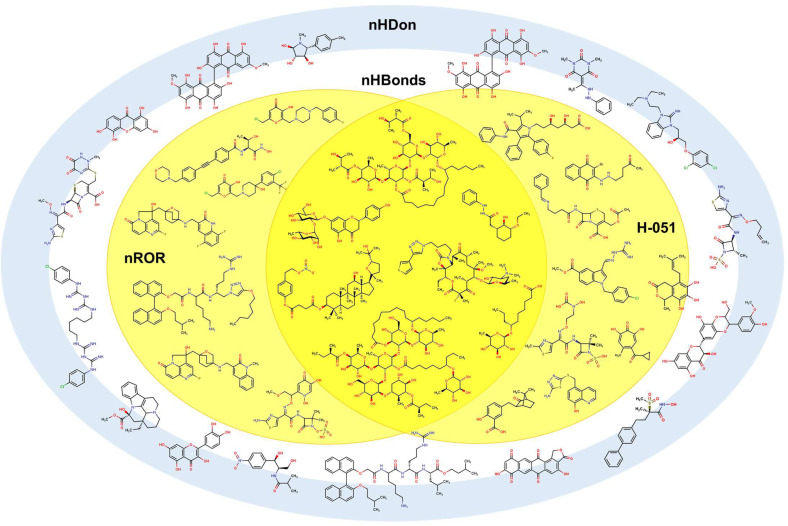
Simplified Venn diagram with representative molecules showing the nHDon, nHBonds, nROR, and H-051 descriptors which appear in molecules **54** to **68**.

One of our remaining questions is which action mechanisms can be exerted by these molecules. To propose one, we constructed a simplified version of the Venn diagram in which it is possible to observe the correlation between the H-051 and the nROR descriptors seen in the isolated molecules. The purpose of this diagram in Figure 18 is to identify molecules with known action mechanisms and with structural similarity (same molecular descriptors) to our compounds. Furthermore, other types of compounds used are also part of the inner set of molecules. These compounds have different structural motifs when compared to compounds **54** to **68**, and they present different mechanisms of action.

**Figure 19:**
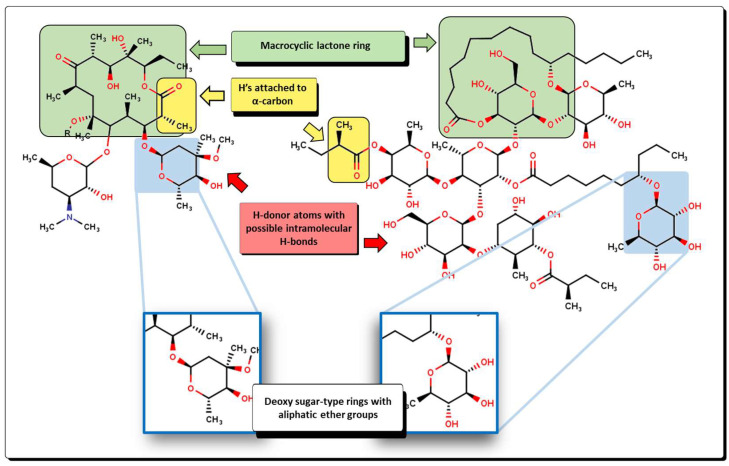
Structural comparison between a macrolide (clarithromycin) and compound **63**, where arrows indicate the descriptors that are shared.

From a structural point of view, compounds **56** and **63** resemble those of the macrolide antibiotics [89]. Examples of macrolides are erythromycin A, oleandomycin, josamycin, and spiramycin, isolated from different microorganisms, as well as many semisynthetic derivatives like clarithromycin, flurithromycin, and other unique compounds like azithromycin. Moreover, the latest new members, the ketolides and fluoroketolides, are also structurally related to the macrolide family. As stated above, when comparing the new molecules with macrolides, several features are shared (Figure 19). Macrolides are well characterized by the presence of a 14- to 16-membered macrocyclic lactone ring to which one or more deoxy sugars are attached. In the case of compounds like **56** and **63**, the macrolactone ring is shown connecting two or three sugar-type rings. Furthermore, because of the relatively high number of carbonyl motifs in macrolides, α-H are also present in great numbers. This is also true for many compounds from **54** to **58** and **65** to **68**, where the ester group is observed. Moreover, a great number of aliphatic ether groups and a great number of oxygen atoms present at the hydroxyl groups and other motifs are also features that are in common. Macrolides are potential bacteriostatic compounds for which one mechanism of action relies on binding to the P site on the 50S subunit of the bacterial ribosome. Because of this, we can suggest that compounds **56** and **64**, among others, could exhibit a similar action on bacteria, thus acting as protein synthesis inhibitors.

**Figure 20:**
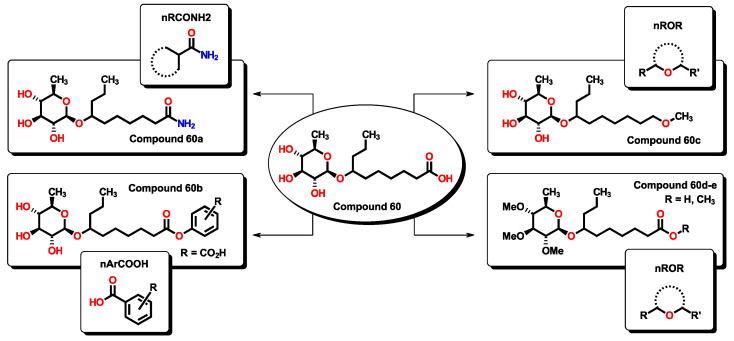
Proposed **60**-derivates which, in accordance with Equation (1), are expected to improve their antibacterial activity by incorporation of specific functional groups.

Finally, compound **60,** being a small molecule, can be considered a lead compound for which specific chemical transformations could improve its efficacy. Compound 60 is expected to be water soluble, having a calculated LogP value below 1.0, hence, with good gastrointestinal (GI) absorption. According to Equation (1), the incorporation of specific functional groups contributes favorably to the expected *p*MIC. In this sense, aromatic carboxylic acids, primary aliphatic amides, and aliphatic ethers can be employed to improve the activity of **60**-derived analogs as they represent simple chemical transformations, as seen in Figure 20. Calculated *p*MIC values for the new derivatives can be seen in Table S7. In all proposed compounds, their predicted *p*MIC values are higher than the lead compound. The physicochemical properties of compounds **60** and derivatives were tested using the SwissADME server, for which specific alerts are given in Figure S4. All derivatives are expected to be soluble in water and predicted to be passively absorbed by the GI tract, and **60e** could also permeate through the blood-brain barrier. Furthermore, all compounds also obey Lipinski’s rules. 

Moreover, molecules isolated from *Ipomoea* share within their structures a deoxy-sugar moiety, as in compound **60**, that could be relevant to their activity. By close inspection of the fragment, we searched for molecules in the ChEMBL database for bioactive compounds which incorporate deoxy sugar in their structures. A wide variety of molecules possesses the motif, from anticancer to anti-allergenic [94,95,96,97,98,99,100,101,102], demonstrating its importance; further research is needed to validate this point. Chemical structures for these compounds can be seen in Figure S3 as well as their predicted ADME properties.

In summary, the model was validated statistically by internal and external parameters, showing good predictive power. This was demonstrated using the model, first applied to the BIOFACQUIM natural products database in search of potential candidates and finally, by exploring the properties of isolated natural products from *Ipomoea* sp. We observed wide antibacterial spectra activity of compounds **56**, **60**, and **63** against several isolated bacterial strains, which agrees with the properties calculated by the model.

## 3. Materials and Methods

### 3.1. Data Set

An initial dataset of 944 compounds was obtained from the literature between 1995 and 2020. These compounds shared the same evaluation method, as follows. To improve the reliability of the data, all the compounds were curated [103,104,105] to point out outliers, uncertainties, and potential errors that could affect the models generated at later stages, which included: (1) removal of mixtures, salts, and inorganic/organometallic compounds; (2) ring aromatization as well as standardization of the carboxyl, nitro, and sulfonyls groups; (3) deletion of duplicates and exclusion of stereoisomers of the same compound, as 3D-molecular descriptors are not used in this work (see below). After data curation, compounds with undefined Minimum Inhibitory Concentration (MIC) values and values greater than 300 µg/mL were removed [106], leaving a final set of 592 molecules for the generation of the models. Finally, logarithmic transformation of MIC values was achieved to normalize the experimental information; a conversion of MIC values from µg/mL to molar concentration (M = mol/L) was done, followed by a transformation to *p*MIC according to:

(4) $$p\mathrm{MIC}=–\log_{10}[\mathrm{MIC}]$$

### 3.2. Calculation of Molecular Descriptors

The structures of the molecules of interest were drawn in Avogadro [107,108] and MarvinSketch [109] (ChemAxon, Budapest, Hungary). For the calculation of molecular descriptors, the Dragon [110] computational package was employed. For most of the molecules, their action mechanism is unknown, as in the case of many natural products. Therefore, because molecular conformation is not considered, only zero-, mono- and bi-dimensional descriptors were calculated. The number of descriptors employed per family for the Genetic Algorithms (GA) technique were as follows: 45 constitutional, 105 topological, 33 connectivity indices, 96 2D autocorrelations, 21 topological charge indices, 93 functional groups, 88 atom-centered fragments, and ten molecular properties. A complete list with the molecular descriptors and biological activities reported as MIC in µg/mL is found in the Supplementary Information (SI, Tables S1 and S2).

### 3.3. Generation of the Mathematical Model

The regression models were built using GA techniques with the Mobydigs software [111]. GA are a statistical method that can be employed for analyzing complex systems that correlate with multiple variables. In an analogous manner to genetic evolution, this approximation allows the selection of the most suitable mathematical models from a large set [112]. Molecular descriptors were used as independent variables, and the experimental MIC value expressed as *p*MIC was used as the dependent variable. The selection of the best model was based on parameter values such as the coefficient of determination ($R^{2}$); additionally, the standard deviation (*s*) and the Fischer test (*F*) were employed. The Y-scrambling test was used to guarantee that the QSPR model was built adequately in terms of correlation obtained by chance. This was performed first by randomly permuting the *p*MIC values of the data set and then using the new column of values with the same variables to generate new models. The procedure was repeated 300 times, and the quality parameters of these new models were compared to the original values of the QSPR model: if the original model has no chance correlation, the new $R^{2}$ and $Q^{2}$ values calculated for the permuted *p*MIC QSPR models will have a significant difference with respect to the original values; otherwise, the model is rejected. Non-collinearity between descriptors is determined using the QUIK rule. Accordingly, the QUIK rule is based on the *K* multivariate correlation index that measures the total correlation of a set of variables as follows:

(5) $$K=\frac{\sum_{j}\left|\frac{\lambda_{j}}{\sum_{j}\lambda_{j}}-\frac{1}{p}\right|}{\frac{2\left(p-1\right)}{p}}$$

where *j* = 1, … , *p* and 0 ≤ *K* ≤ 1

*λ* are the eigenvalues obtained from the correlation matrix of the data set X(n, p), n represents the number of compounds, and p the number of variables (descriptors). The total correlation in the set given by the model descriptors X plus the response $Y\left(K_{XY}\right)$ should always be greater than that measured only in the set of descriptors ($K_{X}$). In other words, if $K_{XY}-K_{X}<\delta K,$ then the model is rejected. The typical δK threshold values for models are between 0.01–0.05. Models that have negative values are not allowed. To detect models with an excess of “good” or “bad” descriptors, the redundancy ($R^{P}$) and overfitting ($R^{N}$) rules were applied. $R^{P}$ is defined as:

(6) $$R^{P}=\overset{p^{+}}{\underset{j=1}{\prod }}\left(1-M_{j}\left(\frac{p}{p-1}\right)\right)$$

with $M_{j}>0$ and $0\leq R^{P}\leq 1$.

While $R^{N}$ is defined as

(7) $$R^{N}=\overset{p^{-}}{\underset{j=1}{\sum }}M_{j}$$

where $M_{j}<0$ and $-1<R^{N}\leq 0$.

Given a regression model with p variables, $R_{jy}$ is the absolute value of the regression coefficient between the *j*th descriptors and the response Y. In this sense, $M_{j}$ can be calculated as follows:

(8) $$M_{j}=\frac{R_{jy}}{R}-\frac{1}{P}\;\;\mathrm{and}-\frac{1}{P}\leq M_{j}\leq \frac{p-1}{p}$$

The redundancy rule establishes that if $R^{P}<t^{P}$, then the model is rejected, where depending on the data, $t^{P}$, which is a user-defined threshold, can range from 0.01 and 0.1, with a suggested value of 0.05. The overfitting rule specifies that if $R^{N}<t^{N}\left(\varepsilon \right)$, then the model is rejected. Calculating $t^{N}\left(\varepsilon \right)$ follows:

(9) $$t^{N}\left(\varepsilon \right)=\frac{p\epsilon -R}{pR}$$

where values of ε can range from 0.01 to 0.1, and p is the number of variables.

### 3.4. QSPR Validation of Prediction Capability

The model reported herein was validated internally by the leave-many-out cross-validation method ($Q^{2}$*_LMO_*) for which the data set was randomly divided into a training set (415 molecules) and a test set (117 molecules) which represented 70% and 30%, respectively of the complete data set. The robustness of the model was further evaluated by bootstrap ($Q^{2}$*_BOOT_*) and $Q^{2}$*_EXT_*. The predictive ability validation was performed by applying the Asymptotic $Q^{2}$ rule (δQ). It is assumed that a good model should have a small difference between fitting and predictive ability, in which significant variations between the $R^{2}$ and $Q^{2}$ values can be due to overfitting or to some not predictable samples [113,114]. The Asymptotic $Q^{2}$ rule evaluates the asymptotic $Q^{2}$ versus the $Q^{2}$ values of the model:

(10) $$Q^{2}{}_{LMO}-Q^{2}{}_{ASYM}<\delta Q$$

If the difference is less than the threshold, typically δQ = −0.005, then the model is rejected. As $Q^{2}$*_LMO_* is asymptotically related to the value of $R^{2}$, it is possible to calculate the $Q^{2}$*_ASYM_* by using the following expression:

(11) $$Q^{2}{}_{ASYM}=1-\left(1-R^{2}\right){\left(\frac{n}{n-p^{\prime }}\right)}^{2}$$

where *n* is the number of objects and $p^{\prime }$ the number of model parameters. To further evaluate the predictive applicability of the model, some statistical parameters developed by Roy et al. were used [45,46,47]. According to the statistical parameters, the following criteria must be present for each evaluation as shown: (i) $Q^{2}>0.5$; (ii) $r^{2}>0.6$; (iii) $\left(r^{2}-r_{0}{}^{2}\right)/r^{2}<0.1$ (or $\left(r^{2}-{r^{\prime }}_{0}{}^{2}\right)/r^{2}<0.1$); (iv) 0.85 ≤ k ≤ 1.15 (or $0.85\leq k^{\prime }\leq 1.15$) and (v) $\left|r_{0}{}^{2}-{r^{\prime }}_{0}{}^{2}\right|<0.3$. Additionally, two parameters derived from the above, $\bar{r_{m}{}^{2}}$ and $\Delta r_{m}{}^{2}$ were also used to evaluate the predictive power of the model [115]. According to, $\bar{r_{m}{}^{2}}$ follows that:

(12) $$\bar{r_{m}{}^{2}}=\frac{\left(r_{m}{}^{2}+{r^{\prime }}_{m}{}^{2}\right)}{2}$$

where $r_{m}{}^{2}$ is calculated as

(13) $$r_{m}{}^{2}=r^{2}\left(1-\sqrt{r^{2}-r_{0}{}^{2}}\right)$$

and where:

(14) $${r^{\prime }}_{m}{}^{2}=r^{2}\left(1-\sqrt{r^{2}-{r^{\prime }}_{0}{}^{2}}\right)$$

While according to (15), $\Delta r_{m}{}^{2}$ is obtained by the following expression:

(15) $$\Delta r_{m}{}^{2}=\left|r_{m}{}^{2}-{r^{\prime }}_{m}{}^{2}\right|$$

The calculation of the $r^{2}$, $r_{0}{}^{2}$, ${r^{\prime }}_{0}{}^{2}$, k and $k^{\prime }$ are shown in the SI.

### 3.5. External Validation

The generated model was validated externally by the prediction of different sets of molecules that were not included in the generation of the model with the following specifications: (1) only molecules with reported MIC values and active towards *A. baumannii* were used, and (2) molecules above 300 µg/mL were excluded. Data curation, as stated above, was performed on a total of 98 molecules, which were drawn in Avogadro, and their molecular descriptors were obtained from the Dragon software package. A complete list of descriptors and references can be found in the SI, Table S5.

### 3.6. Virtual Screening

Five hundred twenty-eight natural products were obtained from the molecular database BIOFACQUIM. To improve the consistency of the data, all the compounds were curated by (1) ring aromatization, (2) standardization of the carboxyl, nitro, and sulfonyl groups if present, and (3) addition of missing bonds where required. The structures of the molecules of interest were drawn in Avogadro, and their molecular descriptors were obtained from the Dragon software package. A complete list of descriptors can be found in Table S6.

### 3.7. Plant Material

Roots of *Ipomoea stans* were collected in the state of Puebla, México. The botanical classification was carried out by Abigail Aguilar, Head of the Instituto Mexicano del Seguro Social Herbarium in Mexico City (IMSSM), and a voucher specimen (number 15077) was deposited at IMSSM. Exudates from the bark of *Ipomoea murucoides* were collected manually on the campus of the Universidad Autónoma del Estado de Morelos (UAEM) in Cuernavaca, Morelos, Mexico. The plant material was identified by Biol. Alejandro Flores and a voucher specimen (No. 22444) were deposited at the Herbarium of the Centro de Investigación en Biodiversidad y Conservación, UAEM. Roots of *Ipomoea purga* were authenticated and donated by M. Sc. Abigail Aguilar, Head of the Instituto Mexicano del Seguro Social Herbarium in Mexico City (IMSSM). A voucher specimen (number 16180) is deposited at IMSSM.

### 3.8. Extraction and Isolation of Compounds

The dried, powdered roots of *I. purga* and *I. stans* (250.0 g each one) were extracted by maceration with MeOH (500 mL × 3) to obtain a dark syrup (25.0 g *I. purge*, and 20.4 g *I. stans*). The dark syrups were extracted with distilled water (3 × 50 mL) and dichloromethane (DCM, 3 × 50 mL) to afford a dark solid (9.3 g *I. purga* and 7.6 g *I. stans*). The dark solids (1.0 g *I. purga*, 1.1 g *I. stans*) were submitted to a C18 column (Supelco, 10 × 15 mm) with a gradient of MeOH:H_2_O (0:100 to 100:0, at increments of 10%), fractions were collected and (0.7 g *I. purga*, 0.6 g *I. stans*) was obtained. The resinous solids were percolated on an activated charcoal column, eluting with MeOH. Fractions of 5 mL were collected and reunited, giving the convolvulin (0.42 g *I. purga* and 0.32 g *I. stans*). Convolvulin of *I. purga* was chromatographed on normal and inverse phase silica gel columns, using mobile phase DCM/MeOH/H_2_O (84:14:2), respectively, with MeOH gradient, yielding 80 mg IPJALB (compound **63**). From the convolvulin of *I. stans* in the same conditions, 30 mg of ISACAF (compound **62**) and 27 mg of ISACAR (compound **61**) were obtained. Exudates from the bark of *Ipomoea murucoides* (15 g) were dried, ground, and dissolved in MeOH to give, after filtration and removal of the solvent, a brown solid material (10 g). The brown solid was dissolved in a mixture of CHCl_3_:MeOH (9:1). This solution was subsequently subjected to passage over a silica gel column eluted with a gradient system of CHCl_3_:MeOH (from 9:1 to 7:3), leading to the separations of two chromatographic fractions. Purification of the less polar chromatographic fraction was carried out by preparative HPLC. Eluates with retention time, tR, and value of 23.5 min were collected and reinjected into the HPLC system to achieve pure IM620 (compound **57**). The rest of the compounds tested for biological essays were given by Dr. Ismael León, purified by similar methods, and used as received.

### 3.9. Bacterial Strains

*Escherichia coli* ATCC 25922 and *Staphylococcus aureus* ATCC 29,213 were purchased from the American Type Culture Collection. MDR clinical isolates, which are non-susceptible to at least one agent in three or more antimicrobial categories and cause nosocomial infections, were obtained from the Center for Research on Infectious Diseases collection of the National Institute of Public Health (Instituto Nacional de Salud Pública), Cuernavaca, Morelos, Mexico. The various strains include the following isolates: *A. baumannii* 9736 and 10324, *E. coli* 10225, *K. pneumoniae* 6411 and 3407-2, and two *P. aeruginosa* 4899 and 4677. These isolates are metallo-β-lactamase-producers and are resistant to all betalactam antibiotics, including cephalosporins, and carbapenems.

### 3.10. Antibacterial Assays

The antibacterial activity of the compounds was qualitatively measured following the Kirby–Bauer method (1996), according to the CLSI (Clinical and Laboratory Standards Institute) recommendations [116]. Briefly, Petri dishes containing Müeller–Hinton agar were sown with bacteria inoculums from 1 to 2 × 10^8^ colony-forming units (CFU)/mL, and then 3 μL of the compound solution was placed over the agar. Incubation time was from 16 to 19 h at 35 ± 2 °C. A halo of growth inhibition was observed as a positive result. Two reference susceptible strains were used: *E. coli* ATCC 25922 and *S. aureus* ATCC 29213.

## 4. Conclusions

There is a great number of compounds that have been biologically tested as antibacterials against *A. baumannii*. Nevertheless, a careful selection of them needs to be done before their use for the generation of a QSPR/QSAR model. Our QSPR model comprises fifteen 2D-dimensional descriptors: one 2D-autocorrelation, two topological, six functional group counts, and six atom-centered fragments descriptors. These molecular descriptors were used to describe their suitability as antibacterial compounds against *A. baumannii*. Additionally, our QSPR model prediction ability, which was fully evaluated by means of different test and validation sets of molecules, allowed us the identification of antibacterial compounds against *A. baumannii* by means of a virtual screening of the BIOFACQUIM database, an interesting source for potential bioactive compounds. The identified compounds, isolated from *Ipomoea* sp., indicated specific molecular features consistent with antibacterial activity. Furthermore, our model proved to be predictively reliable by identifying compounds isolated from local collections of *Ipomoea* sp. that showed a promising wide antibacterial spectrum. Upon experimental testing, compound **60** showed wide antibacterial activity against clinically isolated multidrug-resistant bacteria. Its structure can be found in other compounds also isolated from *Ipomoea*, as in the case of molecule **63**. Molecule **60** could serve as a lead compound for the development of new compounds with possible wide-spectrum antimicrobial activity, like **60a**–**60e** proposed in this work.

## Figures and Tables

**Table 1:** Molecular descriptor values for natural products **26** to **53** with their predicted *p*MIC value. These molecules show the highest values from the BIOFACQUIM database.

MolID	MW	D/Dr06	GATS6m	nROR	nHDon	nHBonds	C018	H051	N075	TI2	*p*MIC
**26**	274.24	101.297	0.399	1	1	0	1	0	0	1.208	5.910
**27**	404.51	208.41	1.05	5	2	2	0	0	0	3.678	5.533
**28**	1167.41	1692.143	0.951	12	16	8	0	2	0	8.304	9.049
**29**	1195.47	1739.777	0.958	12	16	8	0	2	0	8.397	9.076
**30**	1341.63	2360.184	0.965	13	19	10	0	2	0	10.723	9.756
**31**	1690.16	2257.102	1.01	14	13	8	0	9	0	6.18	8.879
**32**	2473.43	6328.172	1.071	19	16	11	0	10	0	20.998	12.845
**33**	2449.3	7015.971	1.082	19	16	11	0	8	0	21.234	13.451
**34**	2445.37	6531.924	1.071	19	16	11	0	10	0	21.416	12.972
**35**	2501.49	6713.547	1.076	19	16	9	0	10	0	21.566	13.485
**36**	272.27	101.297	0.894	1	0	0	1	0	0	1.208	5.579
**37**	346.31	89.966	1.133	2	1	0	1	0	0	1.397	5.920
**38**	560.71	440.654	0.966	4	8	4	0	0	0	5.474	5.695
**39**	250.27	80.687	0.942	1	2	1	1	0	0	1.546	5.580
**40**	1151.41	1669.117	0.921	11	16	9	0	2	0	8.266	8.511
**41**	1179.47	1715.713	0.923	11	16	9	0	2	0	8.347	8.539
**42**	869.18	889.623	0.957	7	10	5	0	2	0	8.006	6.834
**43**	1035.28	1396.119	0.967	10	14	8	0	2	0	8.601	7.984
**44**	1165.44	1695.422	0.921	11	15	10	0	2	0	8.575	8.216
**45**	1193.5	1742.017	0.923	11	15	9	0	2	0	8.623	8.446
**46**	250.27	80.687	0.942	1	2	1	1	0	0	1.546	5.580
**47**	1199.65	1656.598	1.024	10	10	7	0	4	0	8.71	7.644
**48**	1223.67	1189.333	1.054	10	8	6	0	5	0	5.649	7.156
**49**	512.56	409.063	0.805	3	6	2	1	2	1	4.621	6.430
**50**	302.36	106.192	1.753	5	2	1	0	0	0	1.027	5.304
**51**	1369.82	1718.197	1.079	10	8	6	0	5	0	6.149	7.460
**52**	1383.85	1778.609	1.097	10	8	6	0	6	0	6.324	7.386
**53**	2795.76	9251.423	1.099	20	16	8	0	10	0	22.254	15.481

**Table 2:** Molecular descriptors for natural products **54** to **68** with their predicted pMIC value.

MolID	MW	D/Dr06	GATS6m	nROR	nHDon	nHBonds	H051	TI2	*p*MIC
**54**	1139.49	637.544	1.05	8	8	4	5	4.452	6.553
**55**	1095.43	603.051	1.06	8	7	4	5	4.134	6.406
**56**	1155.49	645.374	1.047	8	9	4	5	4.4	6.672
**57**	1225.64	1192.314	1.016	10	9	5	7	5.92	7.289
**58**	1107.49	600.453	1.072	8	6	5	5	4.095	6.080
**59**	1153.38	1669.117	0.957	12	16	11	2	8.266	8.422
**60**	334.46	90.65	0.904	2	4	3	2	4.886	4.374
**61**	326.33	164.888	1.054	1	5	1	0	4.222	4.733
**62**	342.33	166.979	1.09	1	6	2	0	3.98	4.624
**63**	1646.15	2393.949	1.019	13	14	12	6	5.644	8.223
**64**	1019.28	1307.203	0.962	10	13	6	2	9.197	8.242
**65**	855.1	437.562	0.955	8	9	5	2	3.704	6.688
**66**	1093.46	601.959	1.069	8	6	4	5	4.081	6.285
**67**	1037.39	565.718	1.019	8	7	4	5	4.448	6.410
**68**	1123.49	624.213	1.054	8	7	4	5	4.266	6.424

**Table 3:** Biological assays for compounds isolated from *Ipomoea* sp. towards different bacterial strains. For each bacterial strain, essays marked as (+) were positive and (−) negative in susceptibility tests.

Bacterial Strains									
IDSample	*E. coli ATCC 25922*	*S aureus ATCC*	*A baumannii 9736 (1)*	*A. baumannii 10324*	*E. coli 10225*	*K. pneumoniae 6411*	*K. pneumoniae* *3407−2*	*P. aeruginosa 4899*	*P. aeruginosa 4677*
**54**	−	−	−	−	−	+	−	−	−
**55**	−	+	−	−	−	+	−	+	+
**56**	+	+	+	−	−	+	+	+	+
**58**	−	−	−	−	−	−	+	−	−
**60**	+	+	+	+	+	+	+	+	+
**61**	−	−	−	+	−	−	−	−	−
**62**	−	−	−	+	−	−	−	−	−
**63**	−	−	+	+	−	−	−	−	−
**64**	−	−	+	−	−	−	−	−	−

## Data Availability

Data is contained within the article and Supplementary Material.

## Supplementary Materials

The following supporting information can be downloaded at: https://www.mdpi.com/article/10.3390/ph16020250/s1, Figure S1. Scatterplots and Williams plots for each training-test experiment; Figure S2. Scatterplots for each of the molecular descriptors against experimental *p*MIC; Figure S3. Chemical structures from the ChEMBL database, which incorporate the deoxy-sugar moiety found in compound 60; Figure S4. SwissADME predictions for compound 60 and derivatives; Figure S5. BOILED-Egg model for 60-derivatives and compounds (a–f) and 60 to 60h; Table S1. Complete list of descriptors from the Dragon package; Table S2. List of molecules used for the generation of the QSPR model; Table S3. Average values for each molecular descriptor; Table S4. Statistical parameters for the evaluation of the predictive power; Table S5. List of molecules used for the validation test sets, their calculated molecular descriptors, and predicted *p*MIC values; Table S6. List of molecules from the BIOFACQUIM database, their calculated molecular descriptors, and predicted *p*MIC values; Table S7. List of 60-derivatives, their calculated molecular descriptors, and predicted pMIC values.
