# Supplementary material for: Identification of a Family of Glycoside Derivatives Biologically Active against Acinetobacter baumannii and Other MDR Bacteria Using a QSPR Model
Source: Pharmaceuticals (Basel). 2023 Feb 7;16(2):250. doi: 10.3390/ph16020250 (PMC9964118; doi:10.3390/ph16020250)
Supplement: Supplementary file 1 [file pharmaceuticals-16-00250-s001.zip › pharmaceuticals-2181986-supplementary.pdf]

Supplementary Information

Identification of glycoside derivatives against *Acinetobacter baumannii* and other MDR bacteria through a QSPR model

Francisco José Palacios-Can [1,2], Jesús Silva-Sánchez [3], Ismael León-Rivera [2], Hugo Tlahuext [2], Nina Pastor [1] and Rodrigo Said Razo-Hernández [1,*]

[1] Centro de Investigación en Dinámica Celular, Universidad Autónoma del Estado de Morelos, Av. Universidad 1001, Col. Chamilpa, Cuernavaca, Morelos, 62209 México
[2] Centro de Investigaciones Químicas, Universidad Autónoma del Estado de Morelos, Av. Universidad 1001, Col. Chamilpa, Cuernavaca, Morelos, 62209 México.
[3] Centro de Investigación sobre Enfermedades Infecciosas, Instituto Nacional de Salud Pública, Av. Universidad 655, Col. Sta. Ma. Ahuacatitlan, Cuernavaca, Morelos, 62100 México
* Correspondence: rodrigo.razo@uaem.mx

Table of contents

1. **Figure S1**. Scatterplots and Williams plots for each training-test experiment
2. **Figure S2**. Scatterplots for each of the molecular descriptors against experimental *p*MIC
3. **Figure S3**. Chemical structures from the ChEMBL database which incorporate the deoxy-sugar moiety found in compound 60
4. **Figure S4**. SwissADME predictions for compound 60 and derivatives
5. **Figure S5**. BOILED-Egg model for 60-derivatives and compounds (a-f) and 60 to 60h
6. **Table S1**. Complete list of descriptors from the Dragon package
7. **Table S2**. List of molecules used for the generation of the QSPR model
8. **Table S3**. Average values for each molecular descriptor
9. **Table S4**. Statistical parameters for the evaluation of the predictive power
10. **Table S5**. List of molecules used for the validation tests sets, their calculated molecular descriptors, and predicted *p*MIC values
11. **Table S6**. List of molecules from the BIOFACQUIM database, their calculated molecular descriptors, and predicted *p*MIC values.
12. **Table S7**. List of 60-derivatives, their calculated molecular descriptors, and predicted *p*MIC values.

Figure S1. Scatterplots and Williams plots for each training-test experiment.

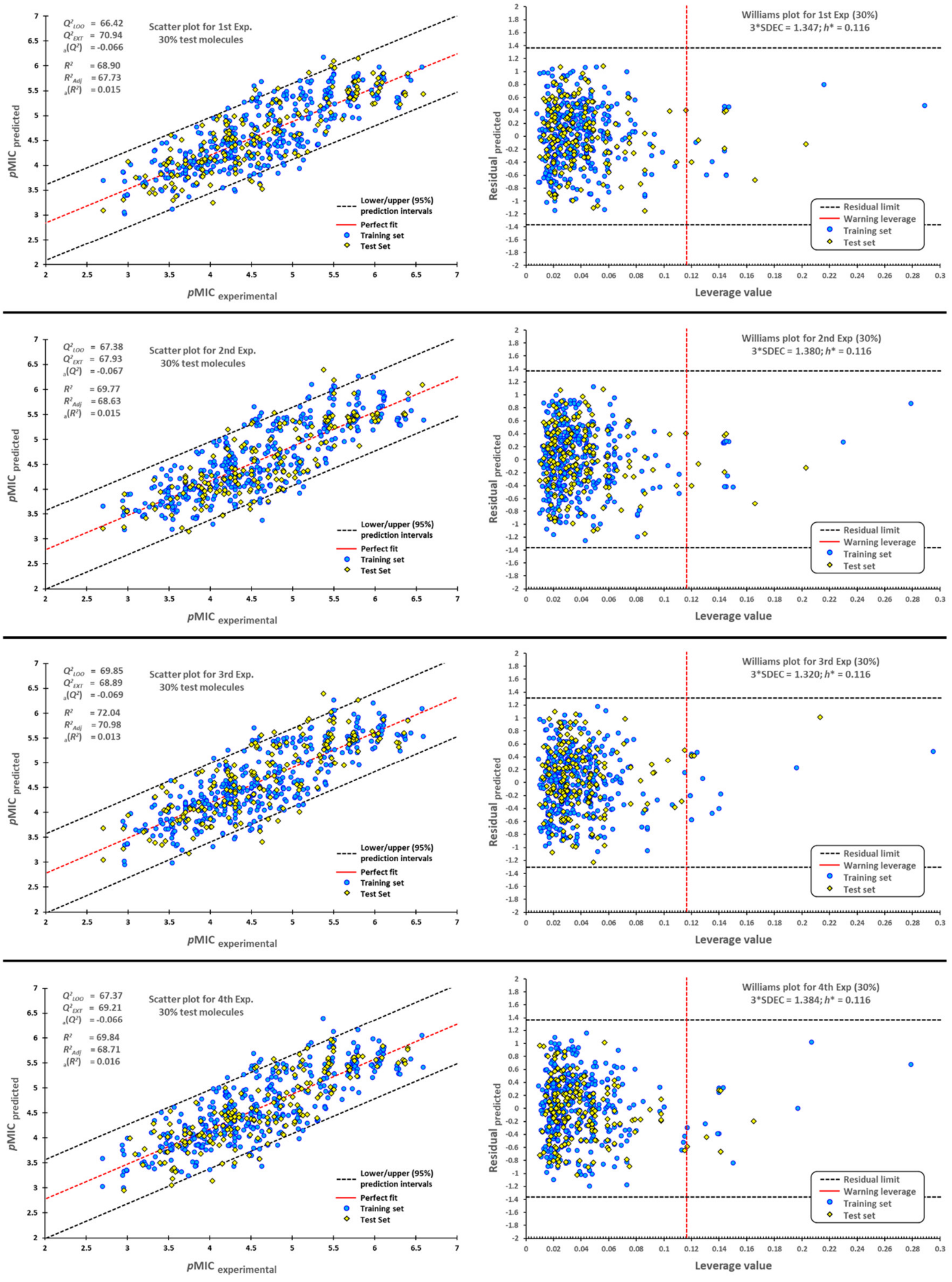

Figure S1. Scatterplots and Williams plots for each training-test experiment (cont.)

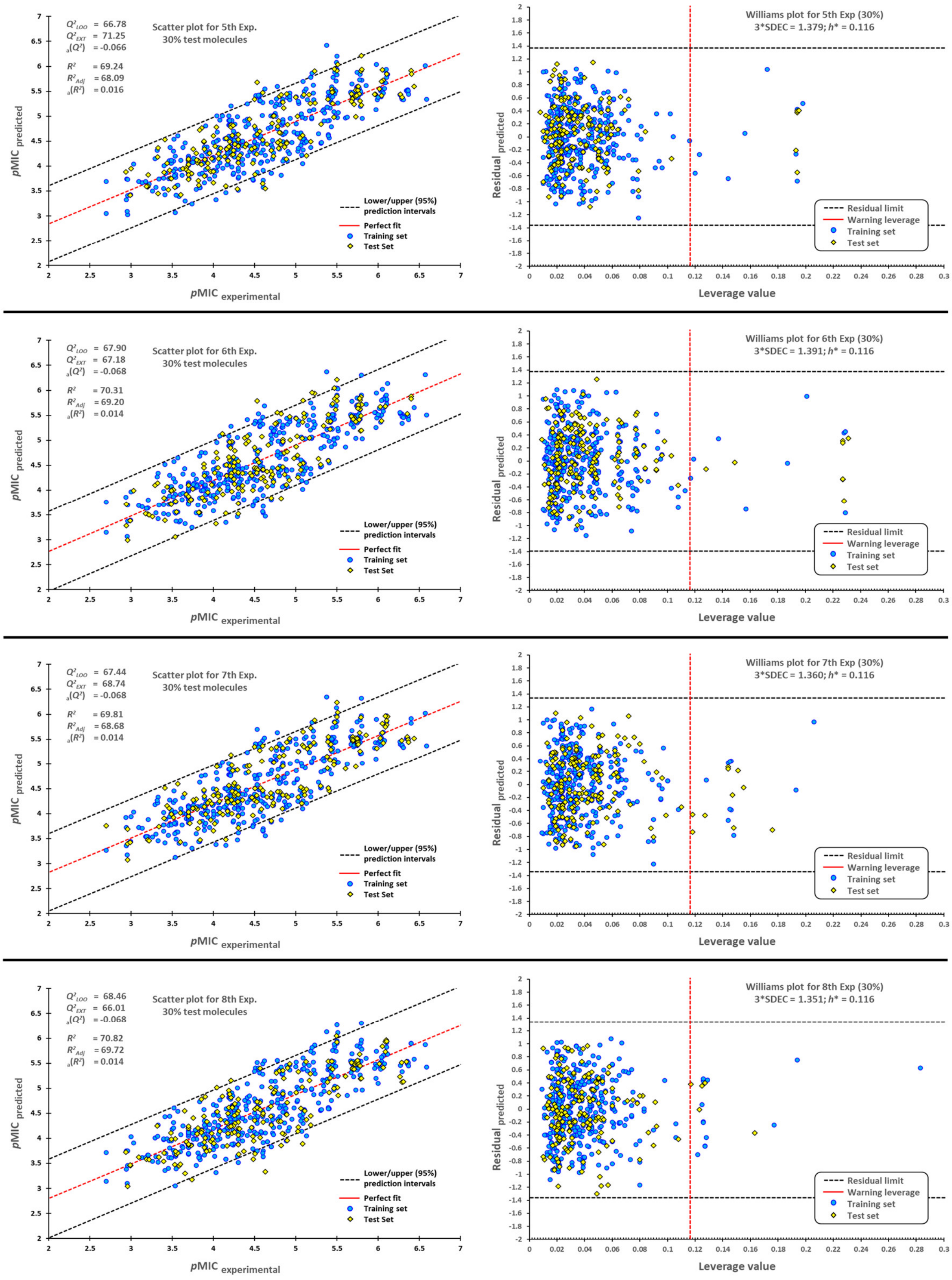

Figure S1. Scatterplots and Williams plots for each training-test experiment (cont.)

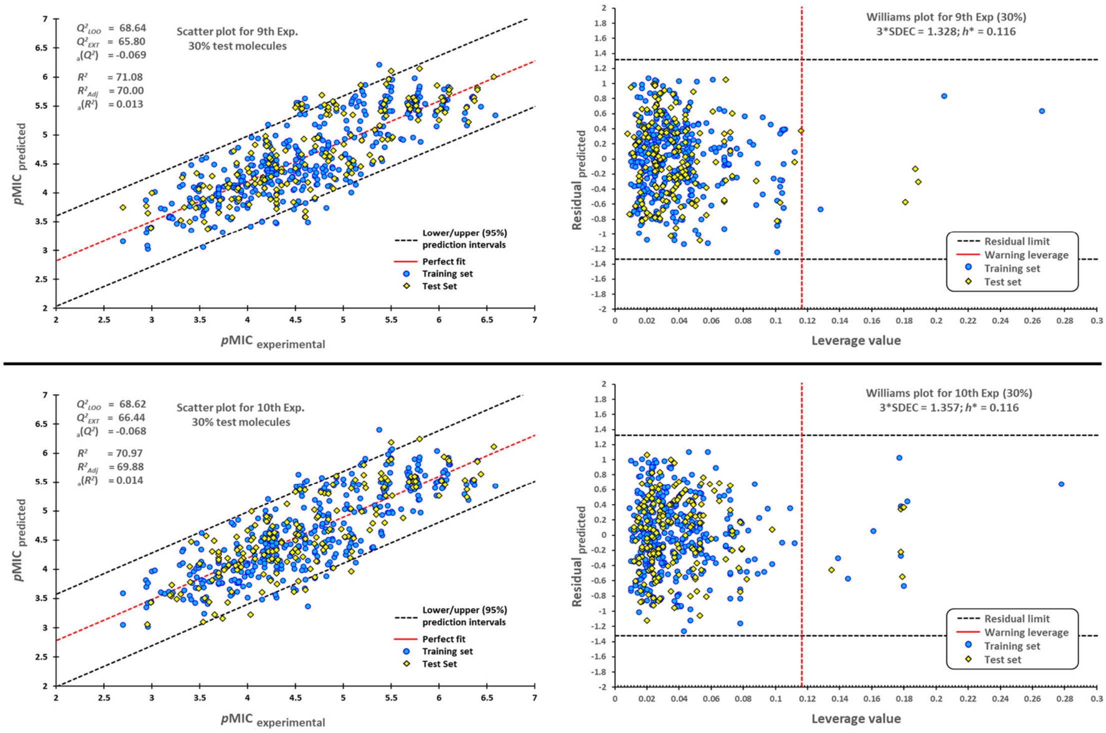

Figure S2. Scatterplots for each of the molecular descriptors against experimental *p*MIC

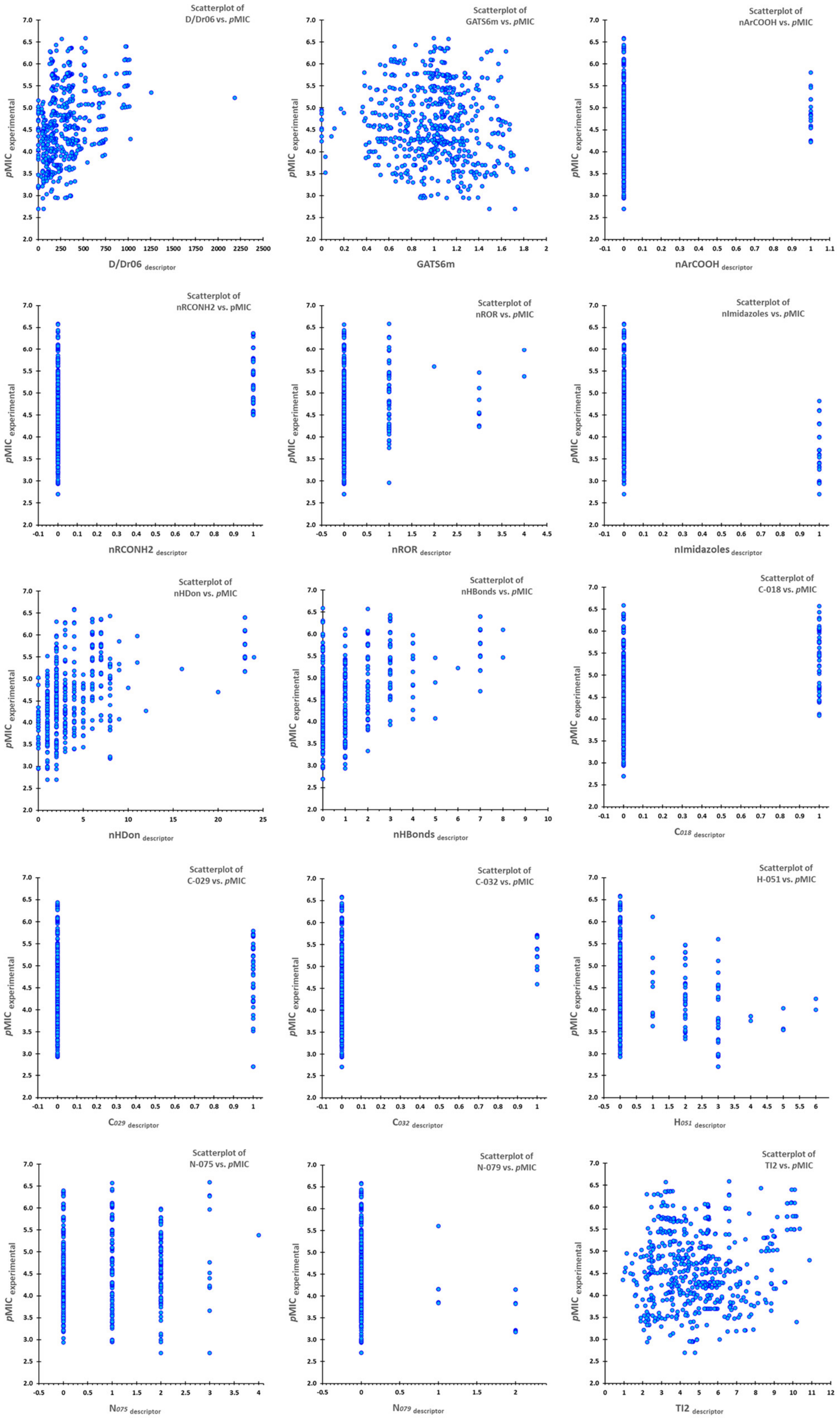

Figure S3. Chemical structures from the ChEMBL database which incorporate the deoxy-sugar moiety found in compound 60 (highlighted in yellow) and their predicted ADMET properties using SwissADME. References for (a) in [1]; for (b) in [2]; for (c) in [3]; for (d) in [4]; for (e) in [5-6]; and for (f) in [7].

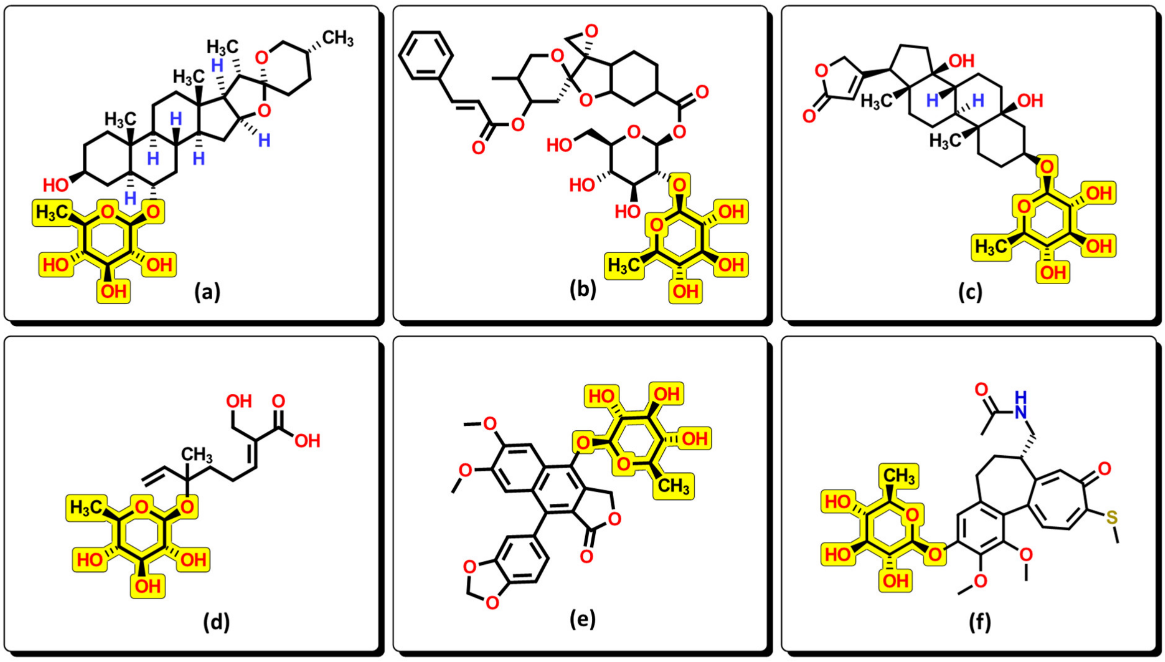

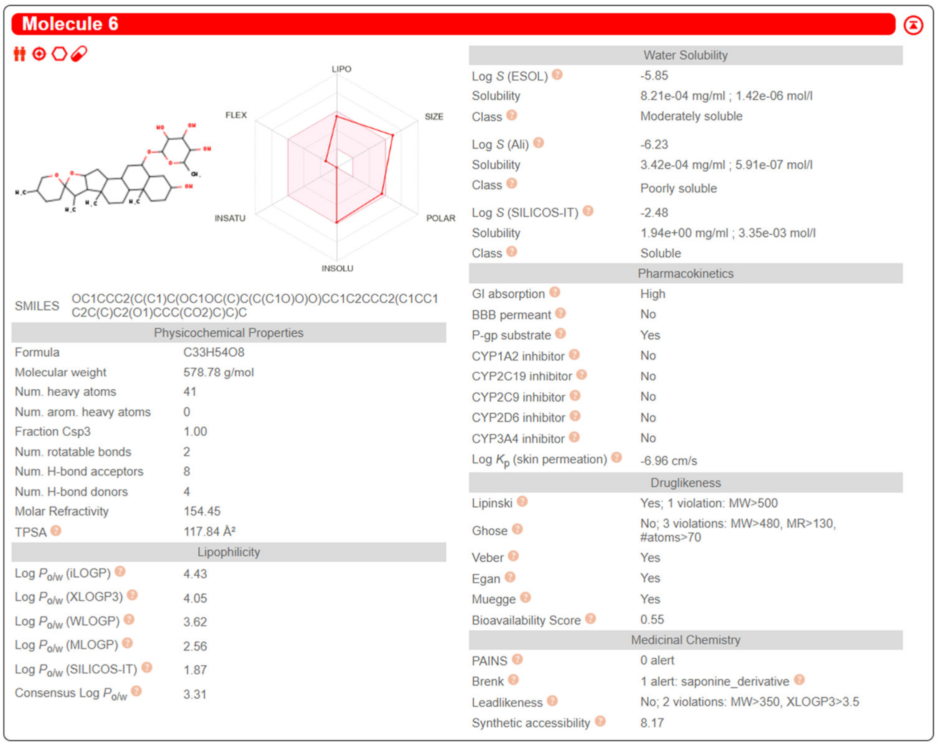

SMILES OC1CCC2(C(C1)C(OC1OC(C)C(C(C1O)O)O)CC1C2CCC2(C1CC1C2C(C)C2(O1)CCC(CO2)C)C)C

Physicochemical Properties	
Formula	C33H54O8
Molecular weight	578.78 g/mol
Num. heavy atoms	41
Num. arom. heavy atoms	0
Fraction Csp3	1.00
Num. rotatable bonds	2
Num. H-bond acceptors	8
Num. H-bond donors	4
Molar Refractivity	154.45
TPSA	117.84 Å²

Lipophilicity	
Log $P_{o/w}$ (iLOGP)	4.43
Log $P_{o/w}$ (XLOGP3)	4.05
Log $P_{o/w}$ (WLOGP)	3.62
Log $P_{o/w}$ (MLOGP)	2.56
Log $P_{o/w}$ (SILICOS-IT)	1.87
Consensus Log $P_{o/w}$	3.31

Water Solubility	
Log *S* (ESOL)	-5.85
Solubility	8.21e-04 mg/ml ; 1.42e-06 mol/l
Class	Moderately soluble
Log *S* (Ali)	-6.23
Solubility	3.42e-04 mg/ml ; 5.91e-07 mol/l
Class	Poorly soluble
Log *S* (SILICOS-IT)	-2.48
Solubility	1.94e+00 mg/ml ; 3.35e-03 mol/l
Class	Soluble

Pharmacokinetics	
GI absorption	High
BBB permeant	No
P-gp substrate	Yes
CYP1A2 inhibitor	No
CYP2C19 inhibitor	No
CYP2C9 inhibitor	No
CYP2D6 inhibitor	No
CYP3A4 inhibitor	No
Log K_p (skin permeation)	-6.96 cm/s

Druglikeness	
Lipinski	Yes; 1 violation: MW>500
Ghose	No; 3 violations: MW>480, MR>130, #atoms>70
Veber	Yes
Egan	Yes
Muegge	Yes
Bioavailability Score	0.55

Medicinal Chemistry	
PAINS	0 alert
Brenk	1 alert: saponine_derivative
Leadlikeness	No; 2 violations: MW>350, XLOGP3>3.5
Synthetic accessibility	8.17

Molecule 7

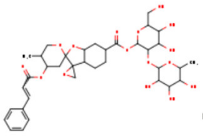

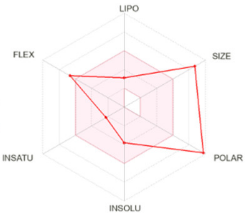

SMILES OCC1OC(OC(=O)C2CCC3C(C2)OC2(C43CO4)OCC(C(C2)OC(=O)/C=C/c2ccccc2)C)C(C(C1O)O)OC1OC(C)C(C(C1O)O)O

Physicochemical Properties	
Formula	C36H48O16
Molecular weight	736.76 g/mol
Num. heavy atoms	52
Num. arom. heavy atoms	6
Fraction Csp3	0.72
Num. rotatable bonds	10
Num. H-bond acceptors	16
Num. H-bond donors	6
Molar Refractivity	174.57
TPSA	232.66 Å²

Lipophilicity	
Log $P_{o/w}$ (iLOGP)	3.37
Log $P_{o/w}$ (XLOGP3)	-0.04
Log $P_{o/w}$ (WLOGP)	-0.97
Log $P_{o/w}$ (MLOGP)	-1.50
Log $P_{o/w}$ (SILICOS-IT)	-0.56
Consensus Log $P_{o/w}$	0.06

Water Solubility	
Log *S* (ESOL)	-3.81
Solubility	1.15e-01 mg/ml ; 1.56e-04 mol/l
Class	Soluble
Log *S* (Ali)	-4.40
Solubility	2.96e-02 mg/ml ; 4.02e-05 mol/l
Class	Moderately soluble
Log *S* (SILICOS-IT)	-0.43
Solubility	2.72e+02 mg/ml ; 3.69e-01 mol/l
Class	Soluble

Pharmacokinetics	
GI absorption	Low
BBB permeant	No
P-gp substrate	Yes
CYP1A2 inhibitor	No
CYP2C19 inhibitor	No
CYP2C9 inhibitor	No
CYP2D6 inhibitor	No
CYP3A4 inhibitor	No
Log K_p (skin permeation)	-10.82 cm/s

Druglikeness	
Lipinski	No; 3 violations: MW>500, NorO>10, NHorOH>5
Ghose	No; 4 violations: MW>480, WLOGP<-0.4, MR>130, #atoms>70
Veber	No; 1 violation: TPSA>140
Egan	No; 1 violation: TPSA>131.6
Muegge	No; 4 violations: MW>600, TPSA>150, H-acc>10, H-don>5
Bioavailability Score	0.17

Medicinal Chemistry	
PAINS	0 alert
Brenk	3 alerts: Three-membered_heterocycle, michael_acceptor_1, more_than_2_esters
Leadlikeness	No; 2 violations: MW>350, Rotors>7
Synthetic accessibility	8.24

Molecule 8

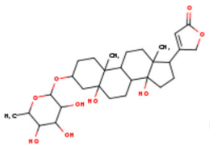

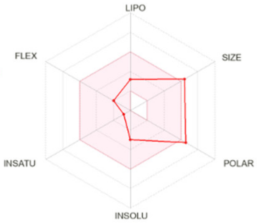

SMILES O=C1OCC(=C1)C1CCC2(C1(C)CCC1C2CCC2(C1(C)CCC(C2)OC1OC(C)C(C(C1O)O)O)O)O

Physicochemical Properties	
Formula	C29H44O9
Molecular weight	536.65 g/mol
Num. heavy atoms	38
Num. arom. heavy atoms	0
Fraction Csp3	0.90
Num. rotatable bonds	3
Num. H-bond acceptors	9
Num. H-bond donors	5
Molar Refractivity	137.18
TPSA	145.91 Å²

Lipophilicity	
Log $P_{o/w}$ (iLOGP)	3.40
Log $P_{o/w}$ (XLOGP3)	0.05
Log $P_{o/w}$ (WLOGP)	1.57
Log $P_{o/w}$ (MLOGP)	1.18
Log $P_{o/w}$ (SILICOS-IT)	1.41
Consensus Log $P_{o/w}$	1.52

Water Solubility	
Log *S* (ESOL)	-3.00
Solubility	5.36e-01 mg/ml ; 9.98e-04 mol/l
Class	Soluble
Log *S* (Ali)	-2.67
Solubility	1.15e+00 mg/ml ; 2.15e-03 mol/l
Class	Soluble
Log *S* (SILICOS-IT)	-1.90
Solubility	6.77e+00 mg/ml ; 1.26e-02 mol/l
Class	Soluble

Pharmacokinetics	
GI absorption	Low
BBB permeant	No
P-gp substrate	Yes
CYP1A2 inhibitor	No
CYP2C19 inhibitor	No
CYP2C9 inhibitor	No
CYP2D6 inhibitor	No
CYP3A4 inhibitor	No
Log K_p (skin permeation)	-9.54 cm/s

Druglikeness	
Lipinski	Yes; 1 violation: MW>500
Ghose	No; 3 violations: MW>480, MR>130, #atoms>70
Veber	No; 1 violation: TPSA>140
Egan	No; 1 violation: TPSA>131.6
Muegge	Yes
Bioavailability Score	0.55

Medicinal Chemistry	
PAINS	0 alert
Brenk	1 alert: saponine_derivative
Leadlikeness	No; 1 violation: MW>350
Synthetic accessibility	6.92

Molecule 9

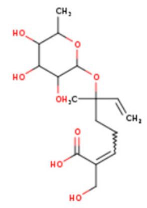

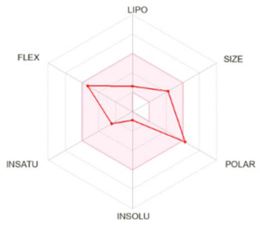

SMILES OC/C(=C\CCC(OC1OC(C)C(C(C1O)O)O)(C=C)C)/C(=O)O

Physicochemical Properties	
Formula	C16H26O8
Molecular weight	346.37 g/mol
Num. heavy atoms	24
Num. arom. heavy atoms	0
Fraction Csp3	0.69
Num. rotatable bonds	8
Num. H-bond acceptors	8
Num. H-bond donors	5
Molar Refractivity	84.59
TPSA	136.68 Å²

Lipophilicity	
Log $P_{o/w}$ (iLOGP)	1.38
Log $P_{o/w}$ (XLOGP3)	-0.92
Log $P_{o/w}$ (WLOGP)	-0.44
Log $P_{o/w}$ (MLOGP)	-0.96
Log $P_{o/w}$ (SILICOS-IT)	-0.12
Consensus Log $P_{o/w}$	-0.21

Water Solubility	
Log *S* (ESOL)	-0.88
Solubility	4.57e+01 mg/ml ; 1.32e-01 mol/l
Class	Very soluble
Log *S* (Ali)	-1.47
Solubility	1.18e+01 mg/ml ; 3.41e-02 mol/l
Class	Very soluble
Log *S* (SILICOS-IT)	0.79
Solubility	2.15e+03 mg/ml ; 6.19e+00 mol/l
Class	Soluble

Pharmacokinetics	
GI absorption	High
BBB permeant	No
P-gp substrate	Yes
CYP1A2 inhibitor	No
CYP2C19 inhibitor	No
CYP2C9 inhibitor	No
CYP2D6 inhibitor	No
CYP3A4 inhibitor	No
Log K_p (skin permeation)	-9.07 cm/s

Druglikeness	
Lipinski	Yes; 0 violation
Ghose	No; 1 violation: WLOGP<-0.4
Veber	Yes
Egan	No; 1 violation: TPSA>131.6
Muegge	Yes
Bioavailability Score	0.56

Medicinal Chemistry	
PAINS	0 alert
Brenk	2 alerts: isolated_alkene, michael_acceptor_1
Leadlikeness	No; 1 violation: Rotors>7
Synthetic accessibility	5.17

Molecule 10

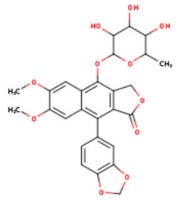

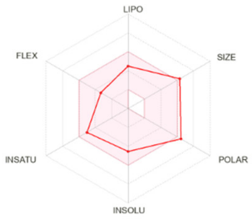

SMILES COc1cc2c(OC3OC(C)C(C(C3O)O)O)c3COC(=O)c3c(c2cc1OC)c1ccc2c(c1)OCO2

Physicochemical Properties	
Formula	C27H26O11
Molecular weight	526.49 g/mol
Num. heavy atoms	38
Num. arom. heavy atoms	16
Fraction Csp3	0.37
Num. rotatable bonds	5
Num. H-bond acceptors	11
Num. H-bond donors	3
Molar Refractivity	130.74
TPSA	142.37 Å²

Lipophilicity	
Log $P_{o/w}$ (iLOGP)	2.99
Log $P_{o/w}$ (XLOGP3)	2.32
Log $P_{o/w}$ (WLOGP)	1.98
Log $P_{o/w}$ (MLOGP)	0.64
Log $P_{o/w}$ (SILICOS-IT)	2.62
Consensus Log $P_{o/w}$	2.11

Water Solubility	
Log *S* (ESOL)	-4.55
Solubility	1.49e-02 mg/ml ; 2.84e-05 mol/l
Class	Moderately soluble
Log *S* (Ali)	-4.95
Solubility	5.93e-03 mg/ml ; 1.13e-05 mol/l
Class	Moderately soluble
Log *S* (SILICOS-IT)	-4.95
Solubility	5.92e-03 mg/ml ; 1.12e-05 mol/l
Class	Moderately soluble

Pharmacokinetics	
GI absorption	Low
BBB permeant	No
P-gp substrate	No
CYP1A2 inhibitor	No
CYP2C19 inhibitor	No
CYP2C9 inhibitor	Yes
CYP2D6 inhibitor	Yes
CYP3A4 inhibitor	Yes
Log K_p (skin permeation)	-7.86 cm/s

Druglikeness	
Lipinski	No; 2 violations: MW>500, NorO>10
Ghose	No; 2 violations: MW>480, MR>130
Veber	No; 1 violation: TPSA>140
Egan	No; 1 violation: TPSA>131.6
Muegge	No; 1 violation: H-acc>10
Bioavailability Score	0.17

Medicinal Chemistry	
PAINS	0 alert
Brenk	0 alert
Leadlikeness	No; 1 violation: MW>350
Synthetic accessibility	5.49

Molecule 11

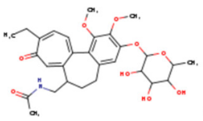

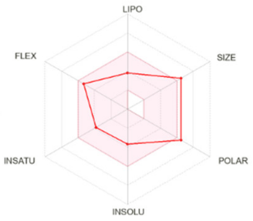

SMILES COc1c(OC)c(OC2OC(C)C(C(C2O)O)O)cc2c1c1ccc(c(=O)cc1C(CC2)CNC(=O)C)CC

Physicochemical Properties	
Formula	C29H37NO9
Molecular weight	543.61 g/mol
Num. heavy atoms	39
Num. arom. heavy atoms	13
Fraction Csp3	0.52
Num. rotatable bonds	8
Num. H-bond acceptors	9
Num. H-bond donors	4
Molar Refractivity	143.94
TPSA	143.78 Å²

Lipophilicity	
Log $P_{o/w}$ (iLOGP)	3.42
Log $P_{o/w}$ (XLOGP3)	1.17
Log $P_{o/w}$ (WLOGP)	1.67
Log $P_{o/w}$ (MLOGP)	0.25
Log $P_{o/w}$ (SILICOS-IT)	3.18
Consensus Log $P_{o/w}$	1.94

Water Solubility	
Log *S* (ESOL)	-3.67
Solubility	1.17e-01 mg/ml ; 2.16e-04 mol/l
Class	Soluble
Log *S* (Ali)	-3.78
Solubility	8.92e-02 mg/ml ; 1.64e-04 mol/l
Class	Soluble
Log *S* (SILICOS-IT)	-5.83
Solubility	8.10e-04 mg/ml ; 1.49e-06 mol/l
Class	Moderately soluble

Pharmacokinetics	
GI absorption	Low
BBB permeant	No
P-gp substrate	Yes
CYP1A2 inhibitor	No
CYP2C19 inhibitor	No
CYP2C9 inhibitor	No
CYP2D6 inhibitor	No
CYP3A4 inhibitor	Yes
Log K_p (skin permeation)	-8.79 cm/s

Druglikeness	
Lipinski	Yes; 1 violation: MW>500
Ghose	No; 3 violations: MW>480, MR>130, #atoms>70
Veber	No; 1 violation: TPSA>140
Egan	No; 1 violation: TPSA>131.6
Muegge	Yes
Bioavailability Score	0.55

Medicinal Chemistry	
PAINS	0 alert
Brenk	0 alert
Leadlikeness	No; 2 violations: MW>350, Rotors>7
Synthetic accessibility	5.92

Figure S4. SwissADME predictions for compound 60 and derivatives

Molecule 1

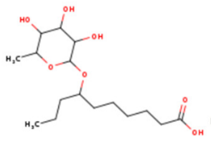

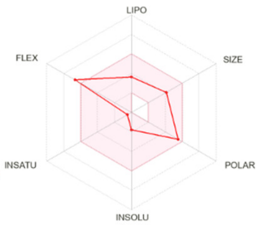

SMILES CCCC(OC1OC(C)C(C(C1O)O)O)CCCCCC(=O)O

Physicochemical Properties	
Formula	C16H30O7
Molecular weight	334.41 g/mol
Num. heavy atoms	23
Num. arom. heavy atoms	0
Fraction Csp3	0.94
Num. rotatable bonds	10
Num. H-bond acceptors	7
Num. H-bond donors	4
Molar Refractivity	84.34
TPSA	116.45 Å²

Lipophilicity	
Log $P_{o/w}$ (iLOGP)	2.22
Log $P_{o/w}$ (XLOGP3)	0.87
Log $P_{o/w}$ (WLOGP)	1.03
Log $P_{o/w}$ (MLOGP)	0.03
Log $P_{o/w}$ (SILICOS-IT)	0.95
Consensus Log $P_{o/w}$	1.02

Water Solubility	
Log *S* (ESOL)	-1.80
Solubility	5.28e+00 mg/ml ; 1.58e-02 mol/l
Class	Very soluble
Log *S* (Ali)	-2.90
Solubility	4.22e-01 mg/ml ; 1.26e-03 mol/l
Class	Soluble
Log *S* (SILICOS-IT)	-0.87
Solubility	4.48e+01 mg/ml ; 1.34e-01 mol/l
Class	Soluble

Pharmacokinetics	
GI absorption	High
BBB permeant	No
P-gp substrate	Yes
CYP1A2 inhibitor	No
CYP2C19 inhibitor	No
CYP2C9 inhibitor	No
CYP2D6 inhibitor	No
CYP3A4 inhibitor	No
Log K_p (skin permeation)	-7.72 cm/s

Druglikeness	
Lipinski	Yes; 0 violation
Ghose	Yes
Veber	Yes
Egan	Yes
Muegge	Yes
Bioavailability Score	0.56

Medicinal Chemistry	
PAINS	0 alert
Brenk	0 alert
Leadlikeness	No; 1 violation: Rotors>7
Synthetic accessibility	5.16

Molecule 2

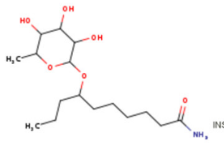

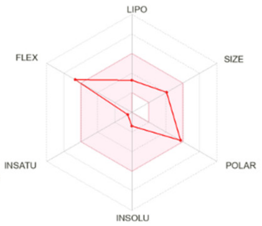

SMILES CCCC(OC1OC(C)C(C(C1O)O)O)CCCCCC(=O)N

Physicochemical Properties	
Formula	C16H31NO6
Molecular weight	333.42 g/mol
Num. heavy atoms	23
Num. arom. heavy atoms	0
Fraction Csp3	0.94
Num. rotatable bonds	10
Num. H-bond acceptors	6
Num. H-bond donors	4
Molar Refractivity	85.47
TPSA	122.24 Å²

Lipophilicity	
Log $P_{o/w}$ (iLOGP)	2.61
Log $P_{o/w}$ (XLOGP3)	0.22
Log $P_{o/w}$ (WLOGP)	0.44
Log $P_{o/w}$ (MLOGP)	-0.37
Log $P_{o/w}$ (SILICOS-IT)	0.71
Consensus Log $P_{o/w}$	0.72

Water Solubility	
Log *S* (ESOL)	-1.39
Solubility	1.37e+01 mg/ml ; 4.11e-02 mol/l
Class	Very soluble
Log *S* (Ali)	-2.35
Solubility	1.50e+00 mg/ml ; 4.50e-03 mol/l
Class	Soluble
Log *S* (SILICOS-IT)	-1.09
Solubility	2.71e+01 mg/ml ; 8.13e-02 mol/l
Class	Soluble

Pharmacokinetics	
GI absorption	High
BBB permeant	No
P-gp substrate	Yes
CYP1A2 inhibitor	No
CYP2C19 inhibitor	No
CYP2C9 inhibitor	No
CYP2D6 inhibitor	No
CYP3A4 inhibitor	No
Log K_p (skin permeation)	-8.18 cm/s

Druglikeness	
Lipinski	Yes; 0 violation
Ghose	Yes
Veber	Yes
Egan	Yes
Muegge	Yes
Bioavailability Score	0.55

Medicinal Chemistry	
PAINS	0 alert
Brenk	0 alert
Leadlikeness	No; 1 violation: Rotors>7
Synthetic accessibility	4.96

Molecule 3

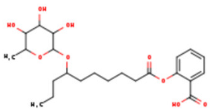

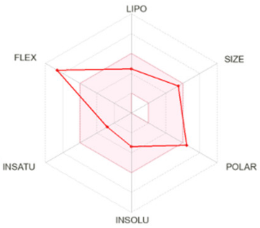

SMILES CCCC(OC1OC(C)C(C(C1O)O)O)CCCCCC(=O)Oc1ccccc1C(=O)O

Physicochemical Properties	
Formula	C23H34O9
Molecular weight	454.51 g/mol
Num. heavy atoms	32
Num. arom. heavy atoms	6
Fraction Csp3	0.65
Num. rotatable bonds	13
Num. H-bond acceptors	9
Num. H-bond donors	4
Molar Refractivity	115.74
TPSA	142.75 Å²

Lipophilicity	
Log $P_{o/w}$ (iLOGP)	2.83
Log $P_{o/w}$ (XLOGP3)	2.29
Log $P_{o/w}$ (WLOGP)	2.25
Log $P_{o/w}$ (MLOGP)	1.03
Log $P_{o/w}$ (SILICOS-IT)	2.21
Consensus Log $P_{o/w}$	2.12

Water Solubility	
Log *S* (ESOL)	-3.38
Solubility	1.89e-01 mg/ml ; 4.16e-04 mol/l
Class	Soluble
Log *S* (Ali)	-4.93
Solubility	5.40e-03 mg/ml ; 1.19e-05 mol/l
Class	Moderately soluble
Log *S* (SILICOS-IT)	-2.98
Solubility	4.77e-01 mg/ml ; 1.05e-03 mol/l
Class	Soluble

Pharmacokinetics	
GI absorption	Low
BBB permeant	No
P-gp substrate	Yes
CYP1A2 inhibitor	No
CYP2C19 inhibitor	No
CYP2C9 inhibitor	No
CYP2D6 inhibitor	No
CYP3A4 inhibitor	Yes
Log K_p (skin permeation)	-7.45 cm/s

Druglikeness	
Lipinski	Yes; 0 violation
Ghose	Yes
Veber	No; 2 violations: Rotors>10, TPSA>140
Egan	No; 1 violation: TPSA>131.6
Muegge	Yes
Bioavailability Score	0.56

Medicinal Chemistry	
PAINS	0 alert
Brenk	1 alert: phenol_ester
Leadlikeness	No; 2 violations: MW>350, Rotors>7
Synthetic accessibility	5.25

Molecule 4

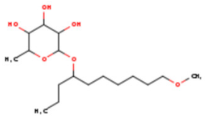

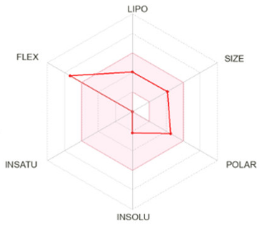

SMILES CCCC(OC1OC(C)C(C(C1O)O)O)CCCCCCOC

Physicochemical Properties	
Formula	C17H34O6
Molecular weight	334.45 g/mol
Num. heavy atoms	23
Num. arom. heavy atoms	0
Fraction Csp3	1.00
Num. rotatable bonds	11
Num. H-bond acceptors	6
Num. H-bond donors	3
Molar Refractivity	88.46
TPSA	88.38 Å²

Lipophilicity	
Log $P_{o/w}$ (iLOGP)	2.45
Log $P_{o/w}$ (XLOGP3)	1.59
Log $P_{o/w}$ (WLOGP)	1.60
Log $P_{o/w}$ (MLOGP)	0.41
Log $P_{o/w}$ (SILICOS-IT)	1.94
Consensus Log $P_{o/w}$	1.60

Water Solubility	
Log *S* (ESOL)	-2.19
Solubility	2.16e+00 mg/ml ; 6.47e-03 mol/l
Class	Soluble
Log *S* (Ali)	-3.06
Solubility	2.93e-01 mg/ml ; 8.77e-04 mol/l
Class	Soluble
Log *S* (SILICOS-IT)	-2.03
Solubility	3.11e+00 mg/ml ; 9.29e-03 mol/l
Class	Soluble

Pharmacokinetics	
GI absorption	High
BBB permeant	No
P-gp substrate	Yes
CYP1A2 inhibitor	No
CYP2C19 inhibitor	No
CYP2C9 inhibitor	No
CYP2D6 inhibitor	No
CYP3A4 inhibitor	No
Log K_p (skin permeation)	-7.21 cm/s

Druglikeness	
Lipinski	Yes; 0 violation
Ghose	Yes
Veber	No; 1 violation: Rotors>10
Egan	Yes
Muegge	Yes
Bioavailability Score	0.55

Medicinal Chemistry	
PAINS	0 alert
Brenk	0 alert
Leadlikeness	No; 1 violation: Rotors>7
Synthetic accessibility	5.52

Molecule 5

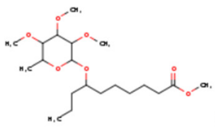

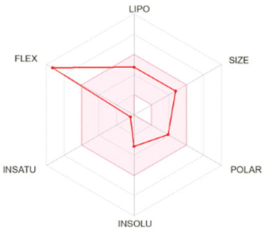

SMILES CCCC(OC1OC(C)C(C(C1OC)OC)OC)CCCCCC(=O)OC

Physicochemical Properties	
Formula	C20H38O7
Molecular weight	390.51 g/mol
Num. heavy atoms	27
Num. arom. heavy atoms	0
Fraction Csp3	0.95
Num. rotatable bonds	14
Num. H-bond acceptors	7
Num. H-bond donors	0
Molar Refractivity	102.85
TPSA	72.45 Å²

Lipophilicity	
Log $P_{o/w}$ (iLOGP)	4.23
Log $P_{o/w}$ (XLOGP3)	2.81
Log $P_{o/w}$ (WLOGP)	3.09
Log $P_{o/w}$ (MLOGP)	0.98
Log $P_{o/w}$ (SILICOS-IT)	3.20
Consensus Log $P_{o/w}$	2.86

Water Solubility	
Log *S* (ESOL)	-3.11
Solubility	3.05e-01 mg/ml ; 7.81e-04 mol/l
Class	Soluble
Log *S* (Ali)	-3.99
Solubility	4.01e-02 mg/ml ; 1.03e-04 mol/l
Class	Soluble
Log *S* (SILICOS-IT)	-3.64
Solubility	8.88e-02 mg/ml ; 2.28e-04 mol/l
Class	Soluble

Pharmacokinetics	
GI absorption	High
BBB permeant	Yes
P-gp substrate	No
CYP1A2 inhibitor	No
CYP2C19 inhibitor	No
CYP2C9 inhibitor	No
CYP2D6 inhibitor	Yes
CYP3A4 inhibitor	No
Log K_p (skin permeation)	-6.69 cm/s

Druglikeness	
Lipinski	Yes; 0 violation
Ghose	Yes
Veber	No; 1 violation: Rotors>10
Egan	Yes
Muegge	Yes
Bioavailability Score	0.55

Medicinal Chemistry	
PAINS	0 alert
Brenk	0 alert
Leadlikeness	No; 2 violations: MW>350, Rotors>7
Synthetic accessibility	5.61

Molecule 12

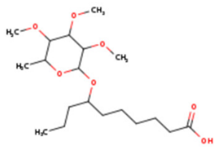

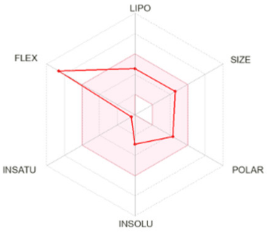

SMILES CCCC(OC1OC(C)C(C(C1OC)OC)OC)CCCCCC(=O)O

Physicochemical Properties	
Formula	C19H36O7
Molecular weight	376.48 g/mol
Num. heavy atoms	26
Num. arom. heavy atoms	0
Fraction Csp3	0.95
Num. rotatable bonds	13
Num. H-bond acceptors	7
Num. H-bond donors	1
Molar Refractivity	98.53
TPSA	83.45 Å²

Lipophilicity	
Log $P_{o/w}$ (iLOGP)	3.27
Log $P_{o/w}$ (XLOGP3)	2.48
Log $P_{o/w}$ (WLOGP)	3.00
Log $P_{o/w}$ (MLOGP)	0.75
Log $P_{o/w}$ (SILICOS-IT)	2.64
Consensus Log $P_{o/w}$	2.43

Water Solubility	
Log *S* (ESOL)	-2.88
Solubility	4.98e-01 mg/ml ; 1.32e-03 mol/l
Class	Soluble
Log *S* (Ali)	-3.88
Solubility	5.00e-02 mg/ml ; 1.33e-04 mol/l
Class	Soluble
Log *S* (SILICOS-IT)	-2.95
Solubility	4.21e-01 mg/ml ; 1.12e-03 mol/l
Class	Soluble

Pharmacokinetics	
GI absorption	High
BBB permeant	No
P-gp substrate	No
CYP1A2 inhibitor	No
CYP2C19 inhibitor	No
CYP2C9 inhibitor	No
CYP2D6 inhibitor	No
CYP3A4 inhibitor	No
Log K_p (skin permeation)	-6.84 cm/s

Druglikeness	
Lipinski	Yes; 0 violation
Ghose	Yes
Veber	No; 1 violation: Rotors>10
Egan	Yes
Muegge	Yes
Bioavailability Score	0.56

Medicinal Chemistry	
PAINS	0 alert
Brenk	0 alert
Leadlikeness	No; 2 violations: MW>350, Rotors>7
Synthetic accessibility	5.51

Molecule 13

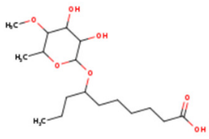

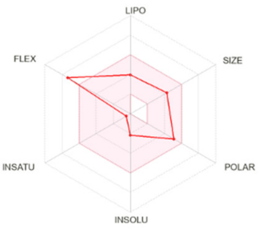

SMILES CCCC(OC1OC(C)C(C(C1O)O)OC)CCCCCC(=O)O

Physicochemical Properties	
Formula	C17H32O7
Molecular weight	348.43 g/mol
Num. heavy atoms	24
Num. arom. heavy atoms	0
Fraction Csp3	0.94
Num. rotatable bonds	11
Num. H-bond acceptors	7
Num. H-bond donors	3
Molar Refractivity	89.07
TPSA	105.45 Å²

Lipophilicity	
Log $P_{o/w}$ (iLOGP)	2.41
Log $P_{o/w}$ (XLOGP3)	1.41
Log $P_{o/w}$ (WLOGP)	1.69
Log $P_{o/w}$ (MLOGP)	0.28
Log $P_{o/w}$ (SILICOS-IT)	1.51
Consensus Log $P_{o/w}$	1.46

Water Solubility	
Log *S* (ESOL)	-2.16
Solubility	2.40e+00 mg/ml ; 6.88e-03 mol/l
Class	Soluble
Log *S* (Ali)	-3.23
Solubility	2.06e-01 mg/ml ; 5.90e-04 mol/l
Class	Soluble
Log *S* (SILICOS-IT)	-1.57
Solubility	9.44e+00 mg/ml ; 2.71e-02 mol/l
Class	Soluble

Pharmacokinetics	
GI absorption	High
BBB permeant	No
P-gp substrate	Yes
CYP1A2 inhibitor	No
CYP2C19 inhibitor	No
CYP2C9 inhibitor	No
CYP2D6 inhibitor	No
CYP3A4 inhibitor	No
Log K_p (skin permeation)	-7.42 cm/s

Druglikeness	
Lipinski	Yes; 0 violation
Ghose	Yes
Veber	No; 1 violation: Rotors>10
Egan	Yes
Muegge	Yes
Bioavailability Score	0.56

Medicinal Chemistry	
PAINS	0 alert
Brenk	0 alert
Leadlikeness	No; 1 violation: Rotors>7
Synthetic accessibility	5.32

Molecule 14

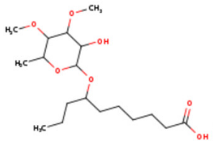

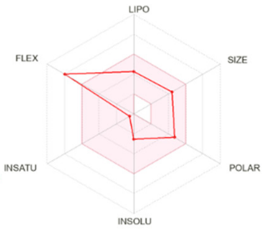

SMILES CCCC(OC1OC(C)C(C(C1O)OC)OC)CCCCCC(=O)O

Physicochemical Properties	
Formula	C18H34O7
Molecular weight	362.46 g/mol
Num. heavy atoms	25
Num. arom. heavy atoms	0
Fraction Csp3	0.94
Num. rotatable bonds	12
Num. H-bond acceptors	7
Num. H-bond donors	2
Molar Refractivity	93.80
TPSA	94.45 Å²

Lipophilicity	
Log $P_{o/w}$ (iLOGP)	3.46
Log $P_{o/w}$ (XLOGP3)	1.95
Log $P_{o/w}$ (WLOGP)	2.34
Log $P_{o/w}$ (MLOGP)	0.52
Log $P_{o/w}$ (SILICOS-IT)	2.07
Consensus Log $P_{o/w}$	2.07

Water Solubility	
Log *S* (ESOL)	-2.52
Solubility	1.09e+00 mg/ml ; 2.99e-03 mol/l
Class	Soluble
Log *S* (Ali)	-3.56
Solubility	1.00e-01 mg/ml ; 2.77e-04 mol/l
Class	Soluble
Log *S* (SILICOS-IT)	-2.26
Solubility	2.00e+00 mg/ml ; 5.51e-03 mol/l
Class	Soluble

Pharmacokinetics	
GI absorption	High
BBB permeant	No
P-gp substrate	No
CYP1A2 inhibitor	No
CYP2C19 inhibitor	No
CYP2C9 inhibitor	No
CYP2D6 inhibitor	No
CYP3A4 inhibitor	No
Log K_p (skin permeation)	-7.13 cm/s

Druglikeness	
Lipinski	Yes; 0 violation
Ghose	Yes
Veber	No; 1 violation: Rotors>10
Egan	Yes
Muegge	Yes
Bioavailability Score	0.56

Medicinal Chemistry	
PAINS	0 alert
Brenk	0 alert
Leadlikeness	No; 2 violations: MW>350, Rotors>7
Synthetic accessibility	5.39

Molecule 15

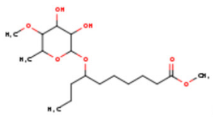

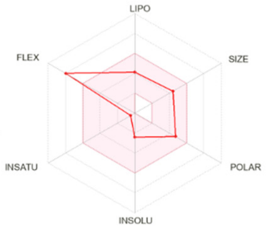

SMILES CCCC(OC1OC(C)C(C(C1O)O)OC)CCCCCC(=O)OC

Physicochemical Properties	
Formula	C18H34O7
Molecular weight	362.46 g/mol
Num. heavy atoms	25
Num. arom. heavy atoms	0
Fraction Csp3	0.94
Num. rotatable bonds	12
Num. H-bond acceptors	7
Num. H-bond donors	2
Molar Refractivity	93.39
TPSA	94.45 Å²

Lipophilicity	
Log $P_{o/w}$ (iLOGP)	3.84
Log $P_{o/w}$ (XLOGP3)	1.74
Log $P_{o/w}$ (WLOGP)	1.78
Log $P_{o/w}$ (MLOGP)	0.52
Log $P_{o/w}$ (SILICOS-IT)	2.07
Consensus Log $P_{o/w}$	1.99

Water Solubility	
Log *S* (ESOL)	-2.39
Solubility	1.47e+00 mg/ml ; 4.06e-03 mol/l
Class	Soluble
Log *S* (Ali)	-3.34
Solubility	1.66e-01 mg/ml ; 4.57e-04 mol/l
Class	Soluble
Log *S* (SILICOS-IT)	-2.26
Solubility	2.00e+00 mg/ml ; 5.51e-03 mol/l
Class	Soluble

Pharmacokinetics	
GI absorption	High
BBB permeant	No
P-gp substrate	Yes
CYP1A2 inhibitor	No
CYP2C19 inhibitor	No
CYP2C9 inhibitor	No
CYP2D6 inhibitor	No
CYP3A4 inhibitor	No
Log K_p (skin permeation)	-7.28 cm/s

Druglikeness	
Lipinski	Yes; 0 violation
Ghose	Yes
Veber	No; 1 violation: Rotors>10
Egan	Yes
Muegge	Yes
Bioavailability Score	0.55

Medicinal Chemistry	
PAINS	0 alert
Brenk	0 alert
Leadlikeness	No; 2 violations: MW>350, Rotors>7
Synthetic accessibility	5.42

Figure S5. BOILED-Egg model for 60-derivatives and compounds (a-f) and 60 to 60h. Compound (b) is seen outside the boundaries of the plot.

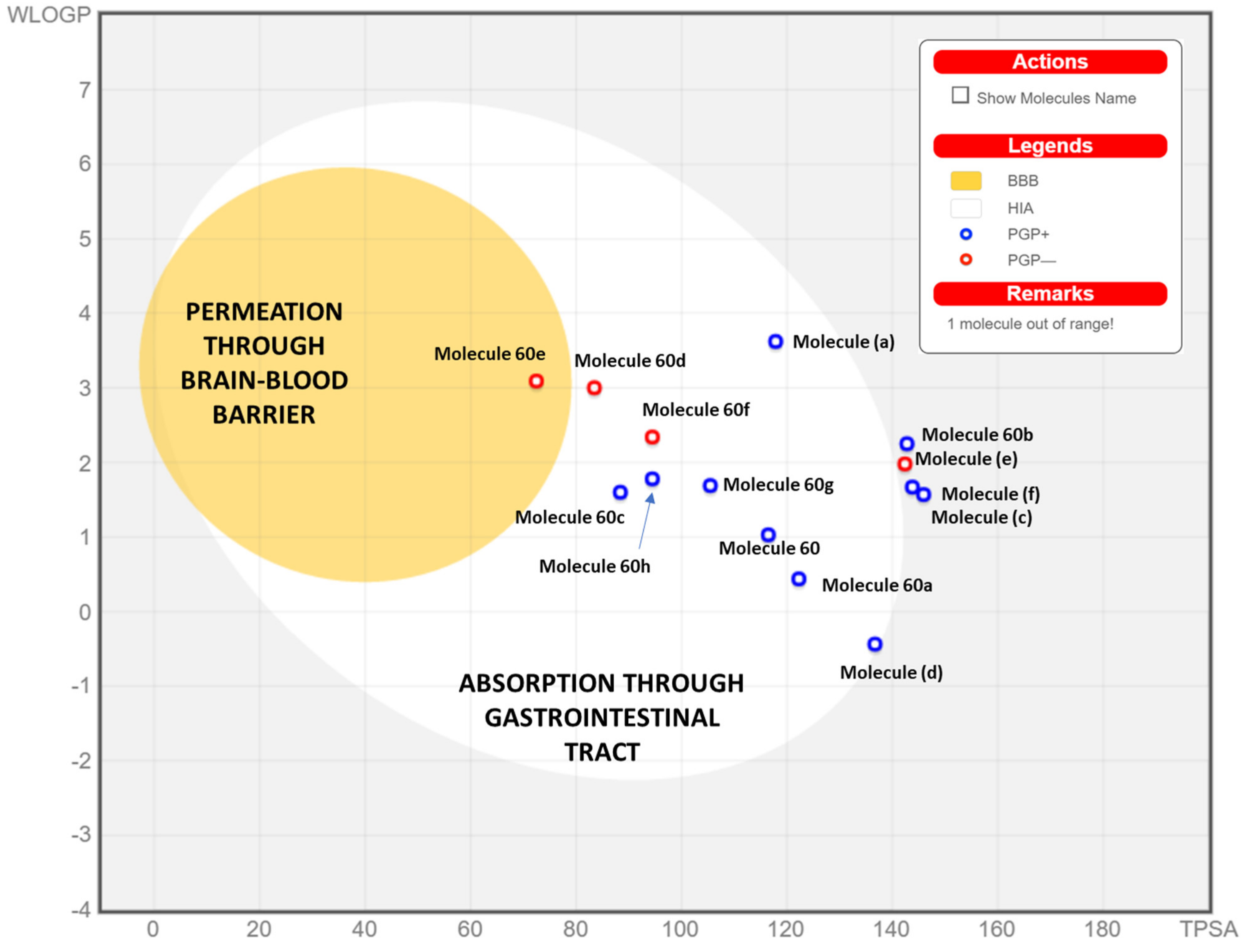

Table S1. Complete list of descriptors from the Dragon package

Dimension	Descriptor family	Descriptor
**0D**	Constitutional	MW, AMW, Sv, Se, Sp, Ss, Mv, Me, Mp, Ms, nAT, nSK, nBT, nBO, nBM, SCBO, ARR, nCIC, nCIR, RBN, RBF, nDB, nTB, nAB, nH, nC, nN, nO. nS, nF, nCL, nBR, nI, nX, nR03, nR04, nR05, nR06, nR07, nR08, nR09, nR10, nR11, nR12, nBnz
**1D**	Functional group counts	nCp, nCs, nCt, nCq, nCrs, nCrt, nCrq, nCar, nCbH, nCb-, nCconj, nR=Cp, nR=Cs, nR=Ct, nR#CH/X, nR#C-, nRCOOH, nArCOOH, nRCOOR, nArCOOR, nRCONH2, nRCONHR, nArCONHR, nRCONR2, nArCONR2, nROCON, nArOCON, nArCOSR, nRCO, nArCO, nCONN, nN=C-N<, nC(=N)N2, nRC=N, nArC=N, nArCNO, nRNH2, nArNH2, nRNHR, nArNHR, nRNR2, nArNR2, nN-N, nRCN, nArCN nN+, nNq, nRNHO, nArNHO, nArNO2, nN(CO)2, nC=N-N<, nROH, nArOH, nOHp, nOHs, nOHt, nROR, nArOR, nRSR, nSO, nS(=O)2, nSO2OH, nSO3OH, nSO2N, nCH2RX, nR=CRX, nCHRX2, nCRX3, nArX, nCXr, nCXr=, nCconjX, nOxiranes, nAzetidines, nBeta-Lactams, nPyrrolidines, nOxolanes, nPyrroles, nPyrazoles, nImidazoles, nFuranes, nThiophenes, nIsoxazoles, nThiazoles, nTriazoles, nPyridines, nPyridazines, nPyrimidines, nPyrazines, nHDon, nHAcc, nHBonds
	Atom-centred fragments	C-001, C-002, C-003, C-004, C-005, C-006, C-007, C-008, C-009, C-010, C-011, C-012, C-013, C-014, C-015, C-016, C-017, C-018, C-019, C-020, C-021, C-022, C-024, C-025, C-026, C-027, C-028, C-029, C-030, C-031, C-032, C-033, C-034, C-035, C-036, C-037, C-038, C-039, C-040, C-041, C-042, C-043, C-044, H-046, H-047, H-048, H-049, H-050, H-051, H-052, H-053, H-054, O-056, O-057, O-058, O-059, O-060, O-061, N-066, N-067, N-068, N-069, N-070, N-071, N-072, N-073, N-074, N-075, N-076, N-077, N-078, N-079, F-081, F-082, F-083, F-084, F-085, Cl-086, Cl-089, Cl-090, Br-091, Br-092, Br-094, S-106, S-107, S-108, S-109, S-110
**2D**	Topological	ZM1, ZM1V, ZM2, ZM2V, Qindex, SNar, HNar, GNar, Xt, Dz, Ram, Pol, LPRS, VDA, MSD, SMTI, SMTIV, GMTI, GMTIV, Xu, SPI, W WA, Har, Har2, QW, TI1, TI2, STN, HyDp, RHyDp, w, ww, Rww, D/D, Wap, WhetZ, Whetm, Whetv, Whete, Whetp, J, JhetZ, Jhetm, Jhetv, Jhete, Jhetp, MAXDN, MAXDP, DELS, TIE, S0K, S1K, S2K, S3K, PHI, BLI, PW2, PW3, PW4, PW5, PJI2, CSI, ECC, AECC, DECC, MDDD, UNIP, CENT, VAR, BAC, Lop, ICR, D/Dr03, D/Dr04, D/Dr05, D/Dr06, D/Dr07, D/Dr08, D/Dr09, D/Dr10, D/Dr11, D/Dr12, T(N..N), T(N..O), T(N..S), T(N..F), T(N..Cl), T(N..Br), T(N..I), T(O..O), T(O..S), T(O..F), T(O..Cl), T(O..Br), T(S..S), T(S..F), T(S..Cl), T(S..Br),T(F..F), T(F..Cl), T(F..Br), T(Cl..Cl), T(Cl..Br), T(Br..Br)

	Connectivity indices	X0, X1, X2, X3, X4, X5, X0A, X1A, X2A, X3A, X4A, X5A, X0v, X1v, X2v, X3v, X4v, X5v, X0Av, X1Av, X2Av, X3Av, X4Av, X5Av, X0sol, X1sol, X2sol, X3sol, X4sol, X5sol, XMOD, RDCHI, RDSQ
	2D-autocorrelations	ATS1m, ATS2m, ATS3m, ATS4m, ATS5m, ATS6m, ATS7m, ATS8m, ATS1v, ATS2v, ATS3v, ATS4v, ATS5v, ATS6v, ATS7v, ATS8v, ATS1e, ATS2e, ATS3e, ATS4e, ATS5e, ATS6e, ATS7e, ATS8e, ATS1p, ATS2p, ATS3p, ATS4p, ATS5p, ATS6p, ATS7p, ATS8p, MATS1m, MATS2m, MATS3m, MATS4m, MATS5m, MATS6m, MATS7m, MATS8m, MATS1v, MATS2v, MATS3v, MATS4v, MATS5v, MATS6v, MATS7v, MATS8v, MATS1e, MATS2e, MATS3e, MATS4e, MATS5e, MATS6e, MATS7e, MATS8e, MATS1p, MATS2p, MATS3p, MATS4p, MATS5p, MATS6p, MATS7p, MATS8p, GATS1m, GATS2m, GATS3m, GATS4m, GATS5m, GATS6m, GATS7m, GATS8m, GATS1v, GATS2v, GATS3v, GATS4v, GATS5v, GATS6v, GATS7v, GATS8v, GATS1e, GATS2e, GATS3e, GATS4e, GATS5e, GATS6e, GATS7e, GATS8e, GATS1p, GATS2p, GATS3p, GATS4p, GATS5p, GATS6p, GATS7p, GATS8p
	Topological charge indices	GGI1, GGI2, GGI3, GGI4, GGI5, GGI6, GGI7, GGI8, GGI9, GGI10, JGI1, JGI2, JGI3, JGI4, JGI5, JGI6, JGI7, JGI8, JGI9, JGI10, JGT
**Other**	Molecular properties	Ui, Hy, Hy2, AMR, TPSA(NO), TPSA(Tot), MLOGP, MLOGP2, ALOGP, ALOGP2

Table S2. List of molecules used for the generation of the QSPR model. A complete list of their calculated molecular descriptors alongside their experimental MIC values (expressed as the *p*MIC) is shown.

No.	MolID	pMIC	D/Dr06	GATS6m	nArCOOH	nRCONH2	nROR	nImidazoles	nHDon	nHBonds	C-018	C-029	C-032	H-051	N-075	N-079	TI2
1	L014	4.062	149.672	0.89	0	0	0	0	2	1	0	0	0	0	0	0	3.967
2	L015	4.018	133.662	0.838	0	0	0	0	4	1	0	0	0	0	0	0	3.992
3	L017	4.481	199.93	0.701	0	0	0	0	2	2	0	0	0	0	0	0	4.745
4	L036	4.699	318.348	0.381	1	0	0	0	2	0	0	0	0	0	1	0	4.44
5	L037	5.018	318.348	0.368	1	0	0	0	2	0	0	0	0	0	1	0	4.44
6	L038	4.761	318.348	0.389	1	0	0	0	2	0	0	0	0	0	1	0	4.44
7	L039	4.224	328.184	0.641	1	0	0	0	2	0	0	0	0	0	1	0	3.912
8	L040	4.543	338.772	0.619	1	0	0	0	2	1	0	0	0	0	1	0	4.16
9	L041	4.844	339.89	0.619	1	0	0	0	2	0	0	0	0	0	1	0	4.201
10	L042	4.844	341.154	0.559	1	0	0	0	2	0	0	0	0	0	1	0	4.215
11	L043	4.561	350.478	0.613	1	0	0	0	2	1	0	0	0	0	1	0	4.407
12	L044	4.591	351.741	0.432	1	0	0	0	2	0	0	0	0	0	1	0	4.422
13	L045	5.504	341.154	0.385	1	0	0	0	2	0	0	0	0	0	1	0	4.215
14	L046	4.826	329.403	0.779	1	0	0	0	2	0	0	0	0	0	1	0	3.915
15	L047	4.844	339.99	0.723	1	0	0	0	2	1	0	0	0	0	1	0	4.164
16	L048	4.844	341.109	0.723	1	0	0	0	2	0	0	0	0	0	1	0	4.205
17	L049	4.844	342.372	0.664	1	0	0	0	2	0	0	0	0	0	1	0	4.22
18	L050	4.862	351.696	0.697	1	0	0	0	2	1	0	0	0	0	1	0	4.412
19	L051	5.193	382.75	0.567	1	0	0	0	2	0	0	0	0	0	1	0	5.086
20	L052	5.463	342.372	0.431	1	0	0	0	2	0	0	0	0	0	1	0	4.22
21	L053	5.193	352.959	0.439	1	0	0	0	2	0	0	0	0	0	1	0	4.428
22	L054	5.805	342.372	0.38	1	0	0	0	2	0	0	0	0	0	1	0	4.22
23	L060	5.603	521.705	1.114	0	0	2	0	7	0	0	0	0	3	0	1	14.471
24	L064	5.026	0	0.949	0	0	1	0	0	0	0	0	0	0	0	0	1.929

25	L065	4.954	0	0	0	0	0	0	1	0	0	0	0	0	0	0	1.151
26	L066	4.866	0	0	0	0	0	0	0	0	0	0	0	0	0	0	1.903
27	L068	3.860	174.064	1.024	0	0	0	0	6	0	0	0	0	1	0	1	6.104
28	L069	4.161	103.491	1.083	0	0	0	0	7	0	0	0	0	0	0	1	6.652
29	L071	4.161	103.491	1.083	0	0	0	0	7	0	0	0	0	0	0	1	6.652
30	L072	3.860	174.064	1.024	0	0	0	0	6	0	0	0	0	1	0	1	6.104
31	L073	4.161	103.491	1.083	0	0	0	0	7	0	0	0	0	0	0	1	6.652
32	L075	4.602	192.468	0.583	0	0	0	0	2	1	0	0	0	0	0	0	5.375
33	L078	4.602	134.267	0.768	0	0	0	1	2	0	0	0	0	0	1	0	5.196
34	L079	4.886	184.526	0.478	0	0	0	0	1	0	0	0	0	0	0	0	5.538
35	L080	4.001	172.414	0.434	0	0	0	0	1	0	0	0	0	0	0	0	4.911
36	L081	4.601	172.414	0.415	0	0	0	0	1	0	0	0	0	0	0	0	4.911
37	L085	4.301	194.887	0.532	0	0	0	1	1	0	0	0	0	0	1	0	6.026
38	L086	4.301	147.072	0.7	0	0	0	0	1	0	0	0	0	0	0	0	5.196
39	L087	4.301	161.596	0.554	0	0	0	0	1	0	0	0	0	0	0	0	5.275
40	L088	4.601	300.32	0.517	0	0	0	0	1	0	0	0	0	0	0	0	5.946
41	L089	4.602	200.544	0.471	0	0	0	0	1	0	0	0	0	0	0	0	5.771
42	L090	4.888	200.015	0.455	0	0	0	0	1	0	0	0	0	0	0	0	5.731
43	L091	4.889	180.977	0.478	0	0	0	0	1	0	0	0	0	0	0	0	5.538
44	L092	4.302	300.32	0.519	0	0	0	0	1	0	0	0	0	0	0	0	5.946
45	L094	4.001	283.527	0.476	0	0	0	0	1	0	0	0	0	2	1	0	5.652
46	L095	4.000	374.177	0.459	0	0	0	0	1	0	0	0	0	0	0	0	6.794
47	L099	4.297	315.792	0.497	0	0	0	0	1	0	0	0	0	0	1	0	5.797
48	L100	4.000	184.811	0.422	0	0	0	0	1	0	0	0	0	6	1	0	5.457
49	L103	5.404	703.965	1.071	0	0	1	0	3	1	0	1	0	0	2	0	5.401
50	L104	5.417	703.965	0.499	0	0	1	0	3	1	0	1	0	0	2	0	5.401
51	L105	4.817	722.498	1.212	0	0	1	0	3	2	0	1	0	0	2	0	5.462
52	L106	5.432	722.498	1.375	0	0	1	0	3	1	0	1	0	0	2	0	5.462
53	L107	5.114	722.498	1.014	0	0	1	0	3	1	0	1	0	0	2	0	5.462

54	L108	5.728	742.503	0.993	0	0	1	0	3	1	0	1	0	0	2	0	5.527
55	L109	4.212	722.498	1.061	0	0	1	0	5	1	0	1	0	0	2	0	5.462
56	L110	4.213	722.498	1.121	0	0	1	0	4	2	0	1	0	0	2	0	5.462
57	L111	4.292	1025.798	1.001	0	0	1	0	3	2	0	1	0	0	2	0	6.193
58	L112	4.512	731.017	1.054	0	0	1	0	3	2	0	1	0	0	2	0	5.344
59	L113	4.199	703.965	1.034	0	0	1	0	3	1	0	0	0	0	2	0	5.401
60	L114	4.200	703.965	1.023	0	0	1	0	3	2	0	1	0	0	3	0	5.401
61	L115	4.801	703.965	1.084	0	0	1	0	3	1	0	0	0	0	1	0	5.401
62	L116	4.790	677.622	1.03	0	0	1	0	2	1	0	0	0	0	2	0	5.215
63	L117	4.501	704.487	1.031	0	0	1	0	2	1	0	0	0	0	2	0	5.403
64	L119	4.200	703.965	1.023	0	0	1	0	2	1	0	0	0	0	2	0	5.401
65	L121	4.210	714.564	0.998	0	0	1	0	2	1	0	0	0	0	2	0	5.405
66	L122	4.218	695.315	1.097	0	0	1	0	2	1	0	0	0	0	2	0	5.309
67	L123	5.105	677.622	0.537	0	0	1	0	2	1	0	0	0	0	2	0	5.215
68	L124	5.406	677.622	0.489	0	0	1	0	2	1	0	0	0	0	2	0	5.215
69	L125	4.229	722.767	0.538	0	0	1	0	2	1	0	0	0	0	2	0	5.537
70	L126	4.229	724.099	0.508	0	0	1	0	2	1	0	0	0	0	2	0	5.549
71	L127	4.188	677.622	1.03	0	0	1	0	2	1	0	1	0	0	2	0	5.215
72	L128	4.189	677.622	1.067	0	0	1	0	2	1	0	1	0	0	3	0	5.215
73	L129	4.189	677.622	1.018	0	0	1	0	2	2	0	1	0	0	3	0	5.215
74	L130	5.090	677.622	1.045	0	0	1	0	2	1	0	0	0	0	1	0	5.215
75	L131	4.805	696.155	1.189	0	0	1	0	2	1	0	0	0	0	1	0	5.301
76	L132	4.819	696.155	1.373	0	0	1	0	2	1	0	0	0	0	1	0	5.301
77	L133	4.217	695.315	1.095	0	0	1	0	2	1	0	0	0	0	1	0	5.309
78	L134	4.244	713.848	1.245	0	0	1	0	2	1	0	0	0	0	1	0	5.379
79	L135	4.244	716.145	0.95	0	0	1	0	2	1	0	0	0	0	1	0	5.408
80	L136	4.244	715.304	0.795	0	0	1	0	2	1	0	0	0	0	1	0	5.412
81	L137	4.789	677.622	1.072	0	0	1	0	3	1	0	1	0	0	2	0	5.215
82	L138	4.187	677.622	1.099	0	0	1	0	3	1	0	1	0	0	2	0	5.215

83	L139	4.186	677.622	1.154	0	0	1	0	4	1	0	1	0	0	2	0	5.215
84	L140	4.200	700.195	1.087	0	0	1	0	2	1	0	1	0	0	2	0	5.384
85	L141	4.501	700.861	1.1	0	0	1	0	2	1	0	1	0	0	2	0	5.39
86	L142	4.211	723.433	1.171	0	0	1	0	2	1	0	1	0	0	2	0	5.535
87	L143	5.078	555.523	1.043	0	0	1	0	2	1	0	0	0	0	1	0	5.021
88	L144	4.283	749.397	0.998	0	0	1	0	2	1	0	0	0	0	1	0	6.278
89	L146	5.090	677.622	1.064	0	0	1	0	2	1	0	0	0	0	2	0	5.215
90	L147	5.395	637.769	0.802	0	0	1	0	2	1	0	0	0	0	1	0	5.854
91	L149	4.175	622.7	1.016	0	0	1	0	2	1	0	0	0	0	1	0	5.662
92	L150	4.507	666.439	0.932	0	0	1	0	2	1	0	0	0	0	1	0	5.861
93	L151	4.507	666.439	0.932	0	0	1	0	2	1	0	0	0	0	1	0	5.861
94	L152	5.061	595.049	1.235	0	0	1	0	2	1	0	0	0	0	1	0	5.35
95	L153	4.159	595.049	1.238	0	0	1	0	2	1	0	0	0	0	2	0	5.35
96	L154	5.397	640.699	0.857	0	0	1	0	2	1	0	0	0	0	1	0	5.883
97	L155	5.085	606.39	0.814	0	0	1	0	2	1	0	0	0	0	1	0	5.313
98	L156	4.501	497.268	0.513	0	0	1	0	2	1	0	0	0	0	2	0	5.391
99	L157	4.197	703.965	1.141	0	0	1	0	3	1	0	1	0	0	2	0	5.401
100	L158	4.524	750.968	1.229	0	0	1	0	2	2	0	1	0	0	3	0	5.632
101	L159	3.906	716.145	1.265	0	0	1	0	2	2	0	0	0	0	1	0	5.408
102	L160	3.924	741.966	0.898	0	0	1	0	3	3	0	0	0	0	1	0	5.578
103	L161	3.885	109.938	0.035	0	0	0	0	0	0	0	1	0	0	2	0	2.553
104	L162	3.523	93.176	0.032	0	0	0	0	0	0	0	1	0	0	2	0	2.88
105	L164	4.703	62.572	1.217	0	0	0	0	3	0	0	0	0	0	0	0	5.895
106	L166	3.792	49.53	1.279	0	0	0	0	3	0	0	0	0	0	0	0	4.242
107	L167	4.701	67.103	1.2	0	0	0	0	3	0	0	0	0	0	0	0	6.44
108	L168	4.090	76.386	1.172	0	0	0	0	3	0	0	0	0	0	0	0	7.512
109	L171	4.386	66.294	1.037	0	0	0	0	3	0	0	0	0	0	0	0	5.715
110	L172	4.700	180.275	1.16	0	0	0	0	3	0	0	0	0	0	0	0	7.544
111	L174	3.518	58.069	0.746	0	0	0	0	1	0	0	0	0	0	0	0	6.351

112	L175	3.840	77.821	1.161	0	0	0	0	1	0	0	0	0	0	0	1	6.666
113	L176	4.719	79.167	0.834	0	0	0	0	2	0	0	0	0	0	0	0	6.457
114	L177	4.719	79.167	1.342	0	0	0	0	2	0	0	0	0	0	0	0	6.508
115	L178	4.448	74.738	0.765	0	0	0	0	2	0	0	0	0	0	0	0	6.346
116	L185	4.529	406.03	1.113	0	0	3	0	3	1	0	0	0	3	2	0	6.139
117	L186	4.249	432.486	1.097	0	0	3	0	2	1	0	0	0	6	2	0	6.976
118	L187	4.529	406.396	1.118	0	0	3	0	3	1	0	0	0	3	2	0	6.143
119	L188	4.537	415.378	1.092	0	0	3	0	1	1	0	0	0	3	2	0	6.425
120	L189	4.537	426.945	1.09	0	0	3	0	1	1	0	0	0	3	2	0	6.641
121	L190	4.552	431.534	1.027	0	0	3	0	2	1	0	0	0	3	2	0	6.912
122	L191	4.258	440.162	1.047	0	0	3	0	1	1	0	0	0	3	2	0	7.138
123	L192	5.109	186.625	0.514	0	0	3	0	1	1	0	0	0	3	2	0	5.588
124	L193	4.843	186.625	0.514	0	0	3	0	1	1	0	0	0	3	2	0	5.588
125	L194	4.232	191.164	0.519	0	0	3	0	3	1	0	0	0	3	3	0	5.869
126	L195	4.262	154.739	0.817	0	0	1	0	3	1	0	0	0	2	0	0	4.816
127	L196	4.115	55.757	1.507	0	0	1	0	2	1	0	0	0	2	0	0	4.425
128	L197	4.226	107.909	1.116	0	0	0	0	2	0	0	0	0	0	0	0	3.001
129	L198	3.483	107.157	0.666	0	0	0	0	2	1	0	0	0	2	0	0	6.514
130	L199	3.499	113.473	0.632	0	0	0	0	2	1	0	0	0	2	0	0	7.099
131	L200	3.514	119.723	0.652	0	0	0	0	2	1	0	0	0	2	0	0	7.677
132	L201	3.542	225.73	1.22	0	0	0	0	2	1	0	0	0	5	0	0	6.6
133	L202	3.555	237.366	1.199	0	0	0	0	2	1	0	0	0	5	0	0	7.182
134	L203	3.568	248.958	1.217	0	0	0	0	2	1	0	0	0	5	0	0	7.761
135	L204	4.059	267.805	0.738	0	0	1	0	1	1	0	0	0	0	0	0	4.737
136	L205	4.718	279.953	0.753	0	0	1	0	1	1	0	0	0	0	0	0	4.892
137	L206	5.060	450.988	0.628	0	0	1	0	1	1	0	0	0	0	0	0	4.528
138	L207	4.652	175.041	0.769	0	0	1	0	1	1	0	0	0	0	0	0	4.452
139	L208	5.030	262.407	1.333	0	0	1	0	2	1	0	0	0	0	0	0	4.145
140	L209	5.053	311.634	0.953	0	0	1	0	2	1	0	0	0	0	0	0	4.572

141	L211	4.305	76.231	1.381	0	0	0	0	3	2	0	0	0	0	0	0	3.792
142	L212	5.219	182.617	1.529	0	0	0	0	2	1	1	0	0	0	0	0	2.378
143	L213	5.517	210.135	1.385	0	0	0	0	1	1	1	0	0	0	0	0	2.27
144	L214	3.093	142.717	1.238	0	0	0	0	1	0	0	0	0	0	2	0	3.463
145	L215	3.833	340.198	1.204	0	0	0	0	1	0	0	0	0	0	2	0	5.859
146	L216	3.002	354.102	0.974	0	0	0	0	1	0	0	0	0	0	2	0	6.1
147	L217	3.815	285.728	1.116	0	0	0	0	0	0	0	0	0	0	2	0	4.525
148	L218	3.890	298.258	0.933	0	0	0	0	0	0	0	0	0	0	2	0	4.664
149	L219	3.138	151.456	1.306	0	0	0	0	1	0	0	0	0	0	2	0	3.604
150	L220	3.866	354.088	1.192	0	0	0	0	1	0	0	0	0	0	2	0	5.972
151	L221	3.933	367.993	0.965	0	0	0	0	1	0	0	0	0	0	2	0	6.215
152	L222	2.946	299.029	1.112	0	0	0	0	0	0	0	0	0	0	2	0	4.685
153	L223	3.618	311.558	0.926	0	0	0	0	0	0	0	0	0	0	2	0	4.824
154	L224	3.792	151.456	1.567	0	0	0	0	1	0	0	0	0	0	2	0	3.604
155	L225	3.905	354.088	1.308	0	0	0	0	1	0	0	0	0	0	2	0	5.972
156	L226	3.967	367.993	1.131	0	0	0	0	1	0	0	0	0	0	2	0	6.215
157	L227	3.890	299.029	1.226	0	0	0	0	0	0	0	0	0	0	2	0	4.685
158	L228	3.954	311.558	1.076	0	0	0	0	0	0	0	0	0	0	2	0	4.824
159	L229	4.483	56.336	1.378	0	0	0	0	1	0	0	0	0	0	1	0	1.473
160	L230	3.889	406.023	1.203	0	0	0	0	3	1	0	0	0	0	0	0	7.246
161	L231	3.953	420.152	1.366	0	0	0	0	3	1	0	0	0	0	0	0	7.369
162	L232	4.195	413.757	1.398	0	0	0	0	3	1	0	0	0	0	0	0	7.049
163	L233	4.259	427.886	1.296	0	0	0	0	3	1	0	0	0	0	0	0	7.19
164	L234	4.174	330.649	1.101	0	0	0	0	1	0	0	0	0	0	0	0	4.91
165	L235	4.240	343.795	1.228	0	0	0	0	1	0	0	0	0	0	0	0	5.064
166	L236	4.179	336.059	1.14	0	0	0	0	1	0	0	0	0	0	0	0	4.645
167	L237	4.245	349.205	1.034	0	0	0	0	1	0	0	0	0	0	0	0	4.82
168	L238	4.174	362.733	0.565	0	0	0	0	1	0	0	0	0	0	2	0	5.935
169	L239	4.240	376.535	1.264	0	0	0	0	1	0	0	0	0	0	2	0	6.066

170	L240	4.179	369.692	0.773	0	0	0	0	1	0	0	0	0	0	2	0	5.689
171	L241	4.245	383.494	1.199	0	0	0	0	1	0	0	0	0	0	2	0	5.841
172	L242	3.755	547.623	0.964	0	0	1	0	2	1	0	0	0	4	0	0	7.935
173	L268	4.044	213.872	0.784	0	0	0	0	6	0	0	0	0	0	0	0	8.098
174	L269	4.801	306.104	0.572	0	0	0	0	10	2	0	0	0	0	0	0	10.882
175	L270	3.835	0	1.207	0	0	0	0	8	1	0	0	0	0	0	2	8.574
176	L271	4.150	0	1.264	0	0	0	0	8	1	0	0	0	0	0	2	9.075
177	L272	4.150	0	1.264	0	0	0	0	8	1	0	0	0	0	0	2	8.987
178	L274	3.821	0	1.209	0	0	0	0	8	1	0	0	0	0	0	2	8.137
179	L275	3.204	0	1.268	0	0	0	0	8	1	0	0	0	0	0	2	7.458
180	L276	3.204	0	1.212	0	0	0	0	8	1	0	0	0	0	0	2	7.419
181	L277	3.176	0	1.219	0	0	0	0	8	1	0	0	0	0	0	2	6.511
182	L278	3.187	0	1.215	0	0	0	0	8	1	0	0	0	0	0	2	6.985
183	L279	3.219	0	1.321	0	0	0	0	8	1	0	0	0	0	0	2	7.897
184	L280	3.398	51.312	1.178	0	0	0	1	4	0	0	0	0	2	1	0	4.534
185	L281	4.602	64.642	1.098	0	0	0	1	4	0	0	0	0	2	1	0	6.099
186	L282	4.600	73.924	1.06	0	0	0	1	4	0	0	0	0	2	1	0	7.145
187	L283	3.398	103.107	0.983	0	0	0	1	4	0	0	0	0	2	1	0	10.186
188	L284	4.605	151.176	0.883	0	0	0	1	4	0	0	0	0	0	1	0	4.839
189	L285	4.302	141.889	0.931	0	0	0	1	4	0	0	0	0	0	1	0	4.526
190	L286	4.302	51.312	1.178	0	0	0	1	4	0	0	0	0	2	1	0	4.534
191	L287	4.302	170.301	0.871	0	0	0	1	4	0	0	0	0	0	1	0	5.596
192	L288	3.998	140.921	0.975	0	0	0	1	4	0	0	0	0	0	1	0	4.357
193	L289	3.399	238.501	0.913	0	0	0	1	4	0	0	0	0	0	1	0	4.707
194	L296	6.113	142.555	1.424	0	0	0	0	3	1	1	2	0	1	1	0	3.079
195	L305	4.566	155.512	0.463	0	0	0	0	2	0	0	0	0	0	0	0	5.396
196	L311	4.288	166.142	0.854	0	0	0	0	3	0	0	0	0	0	0	0	5.563
197	L312	3.209	44.591	1.431	0	0	0	0	2	1	0	0	0	0	0	0	2.068
198	L313	2.937	48.458	1.311	0	0	0	0	2	1	0	0	0	0	0	0	2.247

199	L315	4.142	210.846	1.023	0	0	1	0	1	0	0	0	0	0	0	0	2.192
200	L320	4.881	39.61	0.198	0	0	0	0	3	0	0	0	0	0	0	0	2.468
201	L321	4.981	35.743	0.171	0	0	0	0	3	0	0	0	0	0	0	0	2.425
202	L322	4.904	44.91	1.119	0	0	0	0	3	0	0	0	0	0	0	0	2.839
203	L323	4.529	58.329	0.113	0	0	0	0	3	0	0	1	0	3	1	0	3.229
204	L324	4.629	141.282	0.699	0	0	0	0	3	1	0	0	0	1	0	0	2.348
205	L325	4.629	132.316	1.677	0	0	0	0	2	2	0	0	0	0	0	0	1.096
206	L326	5.181	194.277	1.165	0	0	1	0	1	0	0	0	0	1	0	0	3.985
207	L329	4.898	0	0.36	0	0	0	0	5	0	0	0	0	0	0	0	2.225
208	L330	4.237	30.514	0	1	0	0	0	1	0	0	0	0	0	1	0	1.686
209	L331	4.884	34.943	1.239	0	0	0	0	4	1	0	0	0	0	0	0	2.041
210	L333	4.582	88.45	1.029	0	0	0	0	2	0	0	0	0	2	0	0	6.496
211	L334	4.577	165.599	0.917	0	0	0	0	5	2	0	0	0	0	0	0	2.559
212	L335	5.131	147.779	0.8	0	0	0	0	3	0	0	0	0	0	0	0	2.547
213	L336	4.529	147.133	0.985	0	0	0	0	3	0	1	0	0	0	0	0	2.52
214	L337	5.463	625.259	0.868	0	0	3	0	8	5	0	0	0	2	0	0	5.083
215	L338	4.930	38.248	0	1	0	0	0	4	2	0	0	0	0	0	0	1.623
216	L339	4.780	461.434	0.915	0	0	0	0	5	2	0	0	0	0	0	0	4.105
217	L340	4.780	461.434	0.915	0	0	0	0	5	2	0	0	0	0	0	0	4.105
218	L344	4.527	441.818	0.592	0	0	0	0	4	1	0	0	0	3	0	0	3.984
219	L345	3.604	250.551	0.889	0	0	0	0	1	0	0	0	0	2	0	0	4.325
220	L353	4.831	282.146	1.074	0	1	0	0	7	4	0	0	0	0	0	0	2.822
221	L354	5.433	282.146	1.074	0	1	0	0	7	4	0	0	0	0	0	0	2.822
222	L355	5.445	292.393	1.137	0	1	0	0	7	4	0	0	0	0	0	0	2.919
223	L358	4.900	354.021	0.814	0	1	0	0	8	5	0	0	0	0	0	0	3.905
224	L362	4.842	292.671	1.08	0	1	0	0	7	4	0	0	0	0	0	0	2.885
225	L363	4.563	313.976	1.108	0	1	0	0	7	4	0	0	0	0	0	0	3.076
226	L364	5.784	317.407	1.28	0	1	0	0	7	4	0	0	0	0	0	0	2.821
227	L365	5.794	328.041	1.276	0	1	0	0	7	4	0	0	0	0	0	0	2.861

228	L366	5.511	386.895	1.252	0	1	0	0	7	4	0	0	0	0	0	0	2.959
229	L368	4.853	332.538	1.198	0	1	0	0	7	4	0	0	0	1	0	0	3.86
230	L369	5.387	506.892	0.565	0	0	1	0	2	1	0	2	0	0	4	0	6.425
231	L371	4.764	510.762	1.016	0	0	1	0	4	1	0	2	0	0	3	0	6.658
232	L372	5.968	510.896	0.968	0	0	1	0	4	0	0	2	0	0	3	0	6.602
233	L373	6.286	525.458	1	0	0	1	0	4	0	0	2	0	0	3	0	6.603
234	L374	6.269	510.896	0.968	0	0	1	0	4	0	0	2	0	0	3	0	6.602
235	L375	6.587	525.458	1	0	0	1	0	4	0	0	2	0	0	3	0	6.603
236	L377	5.683	525.458	0.987	0	0	1	0	4	0	0	2	0	0	2	0	6.603
237	L379	5.972	506.892	0.971	0	0	1	0	2	1	0	2	0	0	2	0	6.425
238	L380	5.986	506.892	0.521	0	0	1	0	2	1	0	2	0	0	2	0	6.425
239	L384	5.080	525.457	0.989	0	0	1	0	4	1	0	1	0	0	2	0	6.603
240	L385	5.380	525.457	0.995	0	0	1	0	4	1	0	1	0	0	1	0	6.603
241	L386	5.381	525.457	1.008	0	0	1	0	4	1	0	1	0	0	1	0	6.603
242	L389	4.442	45.91	0.856	0	0	0	0	2	0	0	0	0	0	0	0	2.252
243	L391	4.414	41.697	1.031	0	0	0	0	2	0	0	0	0	0	0	0	2.154
244	L392	4.469	50.123	0.745	0	0	0	0	2	0	0	0	0	0	0	0	2.348
245	L398	4.469	50.123	0.745	0	0	0	0	2	0	0	0	0	0	0	0	2.291
246	L399	3.892	54.337	0.668	0	0	0	0	2	0	0	0	0	0	0	0	2.339
247	L403	4.168	50.289	1.483	0	0	0	0	2	0	0	0	0	0	0	0	2.447
248	L405	3.892	54.502	1.329	0	0	0	0	2	0	0	0	0	0	0	0	2.412
249	L407	3.916	58.88	1.708	0	0	0	0	2	0	0	0	0	0	0	0	2.44
250	L408	3.539	46.28	1.704	0	0	0	0	2	0	0	0	0	0	0	0	2.517
251	L410	3.601	111.773	1.828	0	0	0	0	2	0	0	0	0	0	0	0	2.87
252	L412	4.465	47.066	0.745	0	0	0	0	2	0	0	0	0	0	0	0	2.291
253	L413	4.490	99.575	1.329	0	0	0	0	2	0	0	0	0	0	0	0	2.365
254	L414	3.290	56.64	1.246	0	0	0	0	2	0	0	0	0	0	0	0	2.99
255	L416	3.567	50.123	0.765	0	0	0	0	2	0	0	1	0	0	1	0	2.291
256	L417	5.130	53.99	1.063	0	0	0	0	2	0	0	0	0	0	0	0	2.466

257	L418	4.895	53.99	1.278	0	0	0	0	2	0	0	0	0	0	0	0	2.466
258	L419	4.655	53.99	1.303	0	0	0	0	2	0	0	0	0	0	0	0	2.466
259	L420	4.802	53.99	0.741	0	0	0	0	2	0	0	0	0	0	0	0	2.466
260	L421	3.899	53.99	0.777	0	0	0	0	2	1	0	0	0	0	0	0	2.35
261	L422	4.501	53.99	0.932	0	0	0	0	2	0	0	0	0	0	0	0	2.434
262	L423	3.324	53.99	1.13	0	0	0	0	2	0	0	0	0	0	0	0	2.35
263	L424	4.528	53.99	0.995	0	0	0	0	2	0	0	0	0	0	0	0	2.434
264	L425	3.954	57.857	0.954	0	0	0	0	2	0	0	0	0	0	0	0	2.553
265	L432	5.082	45.564	1.375	0	0	0	0	2	0	0	0	0	0	0	0	2.316
266	L437	3.904	252.108	0.863	0	0	0	0	8	2	0	0	0	0	0	0	8.523
267	L438	4.354	0	0	0	0	0	0	1	0	0	0	0	0	0	0	0.944
268	L439	4.078	54.963	0.863	0	0	0	0	0	0	0	0	0	0	0	0	3.403
269	L440	4.176	58.829	0.777	0	0	0	0	0	0	0	0	0	0	0	0	3.562
270	L441	4.257	58.829	0.617	0	0	0	0	0	0	0	0	0	0	0	0	3.562
271	L442	4.352	63.02	0.95	0	0	0	0	0	0	0	0	0	0	0	0	3.566
272	L443	4.352	58.829	0.087	0	0	0	0	0	0	0	0	0	0	0	0	3.538
273	L444	4.530	0	0	0	0	0	0	2	1	0	0	0	0	0	0	1.045
274	L445	4.530	0	1	0	0	0	0	2	1	0	0	0	1	0	0	2.101
275	L446	5.376	347.287	0.923	0	0	4	0	11	3	0	0	0	0	0	0	4.22
276	L447	5.980	347.287	0.923	0	0	4	0	11	4	0	0	0	0	0	0	4.22
277	L448	5.697	168.463	1.14	0	0	0	0	2	0	0	1	1	0	2	0	3.642
278	L449	5.220	168.346	1.353	0	0	0	0	2	0	0	1	1	0	2	0	3.638
279	L450	5.691	179.497	1.275	0	0	0	0	2	0	0	1	1	0	2	0	3.668
280	L451	5.691	168.463	1.118	0	0	0	0	2	0	0	1	1	0	2	0	3.642
281	L452	5.667	168.463	0.975	0	0	0	0	2	0	0	1	1	0	2	0	3.642
282	L453	5.235	168.346	1.5	0	0	0	0	2	0	0	1	1	0	2	0	3.638
283	L454	4.926	168.346	1.39	0	0	0	0	2	0	0	1	1	0	2	0	3.638
284	L456	5.396	162.172	0.981	0	0	0	0	2	0	0	1	1	0	2	0	3.461
285	L457	5.691	162.172	0.965	0	0	0	0	2	0	0	1	1	0	2	0	3.461

286	L458	5.214	162.172	0.702	0	0	0	0	2	0	0	1	1	0	2	0	3.461
287	L459	5.214	172.42	1.056	0	0	0	0	2	0	0	1	1	0	2	0	3.46
288	L460	5.396	162.33	1.173	0	0	0	0	2	0	0	1	1	0	2	0	3.47
289	L461	5.389	162.33	1.304	0	0	0	0	2	0	0	1	1	0	2	0	3.47
290	L462	4.926	162.33	1.074	0	0	0	0	2	0	0	1	1	0	2	0	3.47
291	L463	4.926	174.094	1.272	0	0	0	0	2	0	0	1	1	0	2	0	4.311
292	L464	5.717	184.508	1.196	0	0	0	0	2	0	0	1	1	0	2	0	4.306
293	L465	5.410	195.632	1.399	0	0	0	0	2	0	0	1	1	0	2	0	4.322
294	L466	5.708	184.508	1.171	0	0	0	0	2	0	0	1	1	0	2	0	4.306
295	L467	5.688	184.508	1.182	0	0	0	0	2	0	0	1	1	0	2	0	4.306
296	L468	6.572	259.735	1.129	0	0	0	0	4	2	1	2	0	0	1	0	3.231
297	L469	5.685	179.497	1.313	0	0	0	0	2	0	0	1	1	0	2	0	3.668
298	L470	5.688	179.497	1.313	0	0	0	0	2	0	0	1	1	0	2	0	3.668
299	L471	5.705	190.531	1.598	0	0	0	0	2	0	0	1	1	0	2	0	3.703
300	L472	4.600	190.531	1.598	0	0	0	0	2	0	0	1	1	0	2	0	3.703
301	L473	5.677	215.803	1.148	0	0	0	0	2	0	0	1	1	0	2	0	4.061
302	L474	5.679	215.803	1.148	0	0	0	0	2	0	0	1	1	0	2	0	4.061
303	L475	5.674	206.31	1.478	0	0	0	0	2	0	0	1	1	0	2	0	3.832
304	L476	5.692	297.345	1.43	0	0	0	0	2	0	0	1	1	0	2	0	3.914
305	L477	5.695	297.345	1.43	0	0	0	0	2	0	0	1	1	0	2	0	3.914
306	L478	5.001	297.345	1.43	0	0	0	0	2	0	0	1	1	0	2	0	3.914
307	L480	4.776	397.711	0.665	0	0	0	0	3	0	0	0	0	0	0	0	5.181
308	L481	3.905	367.903	1.684	0	0	0	0	1	0	0	0	0	1	0	0	3.296
309	L483	3.630	393.025	1.689	0	0	0	0	1	0	0	0	0	1	0	0	3.846
310	L484	3.934	380.201	0.987	0	0	0	0	1	0	0	0	0	1	0	0	3.567
311	L486	4.729	421.699	1.16	0	0	0	0	2	1	0	0	0	0	0	0	3.69
312	L487	5.073	477.062	1.17	0	0	0	0	2	1	0	0	0	0	0	0	4.582
313	L488	4.858	149.672	0.89	0	0	0	0	2	1	0	0	0	0	0	0	3.967
314	L489	4.921	177.213	0.701	0	0	0	0	2	1	0	0	0	0	0	0	4.69

315	L490	5.184	161.203	0.673	0	0	0	0	4	1	0	0	0	0	0	0	4.636
316	L491	3.713	98.826	1.35	0	0	0	0	1	1	0	0	0	0	0	0	6.931
317	L492	3.672	90.769	1.544	0	0	0	0	1	0	0	0	0	0	0	0	6.714
318	L493	4.728	0	0	0	0	0	0	1	0	0	0	0	2	0	0	1.437
319	L494	4.501	0	0	0	0	0	0	1	0	0	0	0	0	0	0	1.344
320	L498	4.371	160.015	1.004	0	0	0	0	2	2	0	0	0	0	0	0	2.23
321	L499	4.667	136.154	1.637	0	0	0	0	1	0	1	0	0	0	2	0	3.206
322	L500	4.397	151.708	1.614	0	0	0	0	1	0	1	0	0	0	2	0	3.774
323	L501	4.427	167.777	1.539	0	0	0	0	1	0	1	0	0	0	2	0	4.282
324	L502	4.696	147.882	1.614	0	0	0	0	1	0	1	0	0	0	2	0	3.774
325	L503	4.711	254.68	1.592	0	0	0	0	1	0	1	0	0	0	2	0	4.037
326	L504	4.412	254.68	1.521	0	0	1	0	1	0	1	0	0	0	2	0	4.037
327	L505	4.110	254.68	1.592	0	0	0	0	2	0	1	0	0	0	2	0	4.037
328	L506	4.382	144.225	1.615	0	0	0	0	2	0	1	0	0	0	2	0	3.6
329	L507	4.079	143.192	1.615	0	0	0	0	2	0	1	0	0	0	2	0	3.6
330	L508	4.410	156.578	1.571	0	0	0	0	2	0	1	0	0	0	2	0	4.259
331	L509	4.726	271.47	1.573	0	0	0	0	2	0	1	0	0	0	2	0	4.56
332	L510	4.439	169.167	1.575	0	0	0	0	2	0	1	0	0	0	2	0	4.839
333	L511	5.623	271.47	1.573	0	0	0	0	2	0	1	0	0	0	2	0	4.56
334	L512	5.340	283.801	1.27	0	0	0	0	2	0	1	0	0	0	2	0	4.85
335	L513	5.958	283.801	0.564	0	0	0	0	2	0	1	0	0	0	2	0	4.85
336	L514	4.750	296.637	1.512	0	0	0	0	2	0	1	0	0	0	2	0	5.141
337	L515	4.734	283.801	1.575	0	0	0	0	2	0	1	0	0	0	2	0	4.85
338	L516	4.734	288.136	1.58	0	0	0	0	2	0	1	0	0	0	2	0	5.107
339	L517	4.752	300.648	1.21	0	0	0	0	2	0	1	0	0	0	2	0	5.425
340	L518	3.885	158.99	0.417	0	0	0	0	5	1	0	0	0	2	0	0	3.058
341	L519	4.212	185.127	0.877	0	0	0	0	5	1	0	0	0	2	0	0	3.82
342	L520	4.215	170.087	0.424	0	0	0	0	3	1	0	0	0	2	0	0	3.538
343	L521	3.896	170.087	0.744	0	0	0	0	3	1	0	0	0	2	0	0	3.538

344	L522	6.293	327.163	1.095	0	1	0	0	6	3	0	0	0	0	0	0	2.228
345	L523	4.800	341.264	1.09	0	1	0	0	6	3	0	0	0	0	0	0	2.322
346	L524	5.420	351.557	1.096	0	1	0	0	6	3	0	0	0	0	0	0	2.4
347	L525	4.764	272.85	1.144	0	1	0	0	6	3	0	0	0	0	0	0	2.482
348	L526	5.439	352.22	1.086	0	1	0	0	7	3	0	0	0	0	0	0	3.53
349	L527	6.028	336.637	1.096	0	1	0	0	7	3	0	0	0	0	0	0	3.301
350	L543	3.657	87.51	1.537	0	0	0	0	3	1	0	0	0	0	3	0	3.576
351	L544	3.856	401.627	0.909	0	0	0	0	1	0	0	0	0	4	0	0	7.489
352	L545	3.856	401.627	0.909	0	0	0	0	1	0	0	0	0	4	0	0	7.489
353	L546	4.064	102.989	0.652	0	0	0	0	2	2	0	1	0	0	1	0	3.469
354	L547	3.805	120.83	0.918	0	0	0	0	3	2	0	1	0	2	1	0	4.284
355	L548	4.158	381.441	0.393	0	0	0	0	4	1	0	0	0	2	0	0	5.344
356	L549	3.857	381.441	0.393	0	0	0	0	4	0	0	0	0	2	0	0	5.344
357	L550	3.765	58.186	1.3	0	0	0	0	4	0	0	0	0	0	1	0	2.131
358	L551	3.440	28.346	1.238	0	0	0	0	5	0	0	0	0	0	2	0	2.308
359	L555	4.779	525.457	1.072	0	0	1	0	3	0	0	0	0	0	1	0	6.603
360	L556	5.727	334.46	1.096	0	1	0	0	6	3	0	0	0	0	0	0	3.245
361	L557	6.043	342.352	1.07	0	1	0	0	6	3	0	0	0	0	0	0	3.189
362	L558	5.453	342.352	0.897	0	1	0	0	6	3	0	0	0	0	0	0	3.189
363	L559	5.751	351.318	1.126	0	1	0	0	6	3	0	0	0	0	0	0	3.14
364	L560	5.772	372.085	1.046	0	1	0	0	6	3	0	0	0	0	0	0	3.068
365	L561	5.471	371.198	1.035	0	1	0	0	6	3	0	0	0	0	0	0	3.063
366	L562	5.182	383.722	1.093	0	1	1	0	6	3	0	0	0	0	0	0	3.056
367	L567	5.771	372.085	1.051	0	1	0	0	7	3	0	0	0	0	0	0	3.068
368	L568	4.597	401.817	1.014	0	1	0	0	6	3	0	0	0	0	0	0	2.991
369	L569	4.890	395.359	1.065	0	1	0	0	6	3	0	0	0	0	0	0	3.069
370	L574	5.103	310.146	1.119	0	1	0	0	7	3	0	0	0	0	0	0	2.875
371	L575	5.729	338.877	1.096	0	1	0	0	7	3	0	0	0	0	0	0	3.333
372	L576	6.041	353.583	1.086	0	1	0	0	7	3	0	0	0	0	0	0	3.608

373	L577	5.742	354.209	1.09	0	1	1	0	7	3	0	0	0	0	0	0	3.641
374	L578	5.416	323.425	1.107	0	1	0	0	6	3	0	0	0	0	0	0	3.049
375	L579	5.428	337.45	1.096	0	1	0	0	6	3	0	0	0	0	0	0	3.255
376	L581	5.739	345.772	1.086	0	1	0	0	6	3	0	0	0	0	0	0	3.452
377	L582	5.749	473.185	1.077	0	1	0	0	6	3	0	0	0	0	0	0	3.663
378	L583	5.138	349.166	1.075	0	1	0	0	7	4	0	0	0	0	0	0	3.46
379	L584	5.450	364.498	1.079	0	1	1	0	6	3	0	0	0	0	0	0	3.7
380	L585	5.159	379.83	1.111	0	1	0	0	6	3	0	0	0	0	0	0	3.944
381	L586	4.567	396.315	1.062	0	1	0	0	7	3	0	0	0	3	0	0	4.245
382	L587	4.844	364.498	1.095	0	1	0	0	6	3	0	0	0	1	0	0	3.7
383	L588	5.742	349.166	0.986	0	1	0	0	6	3	0	0	0	0	0	0	3.46
384	L591	5.732	332.063	1.08	0	1	0	0	7	3	0	0	0	0	0	0	3.034
385	L592	6.344	344.528	1.07	0	1	0	0	7	3	0	0	0	0	0	0	3.242
386	L593	6.356	360.112	1.06	0	1	0	0	7	3	0	0	0	0	0	0	3.458
387	L594	6.033	331.317	1.08	0	1	0	0	6	3	0	0	0	0	0	0	3.007
388	L595	5.756	357.058	0.998	0	1	0	0	6	3	0	0	0	0	0	0	3.392
389	L596	6.365	483.003	1.052	0	1	0	0	6	3	0	0	0	0	0	0	3.586
390	L597	5.753	355.735	1.126	0	1	0	0	7	3	0	0	0	0	0	0	3.216
391	L598	6.353	353.495	1.126	0	1	0	0	7	3	0	0	0	0	0	0	3.188
392	L599	6.365	370.441	1.117	0	1	0	0	7	3	0	0	0	0	0	0	3.462
393	L600	5.462	369.078	1.117	0	1	0	0	7	3	0	0	0	0	0	0	3.392
394	L601	5.742	340.283	1.136	0	1	0	0	6	3	0	0	0	0	0	0	2.972
395	L602	6.367	366.024	1.012	0	1	0	0	6	3	0	0	0	0	0	0	3.33
396	L603	5.762	362.63	1.117	0	1	0	0	6	3	0	0	0	0	0	0	3.322
397	L604	5.440	335.97	1.178	0	1	0	0	7	3	0	0	0	0	0	0	2.815
398	L605	5.473	380.033	1.143	0	1	1	0	7	3	0	0	0	0	0	0	3.425
399	L606	4.548	349.249	1.17	0	1	0	0	6	3	0	0	0	0	0	0	2.942
400	L607	4.567	371.596	1.154	0	1	0	0	6	3	0	0	0	0	0	0	3.264
401	L608	5.081	284.407	1.027	0	1	0	0	6	3	0	0	0	0	0	0	2.588

402	L610	5.710	301.264	1.034	0	1	0	0	6	3	0	0	0	0	0	0	2.605
403	L611	4.517	310.231	1.087	0	1	0	0	6	3	0	0	0	0	0	0	2.625
404	L612	5.125	328.746	1.052	0	1	0	0	7	3	0	0	0	0	0	0	3.178
405	L613	4.513	316.857	1.076	0	1	0	0	6	3	0	0	0	0	0	0	2.956
406	L615	4.778	282.694	1.151	0	1	0	0	8	3	0	0	0	0	0	0	2.569
407	L617	4.501	304.304	1.181	0	1	0	0	6	3	0	0	0	0	0	0	2.784
408	L621	2.959	187.03	1.261	0	0	0	1	0	0	0	0	0	3	1	0	4.498
409	L622	2.955	187.746	1.392	0	0	0	1	1	0	0	0	0	3	1	0	4.494
410	L624	2.943	215.641	0.899	0	0	0	1	1	0	0	0	0	3	1	0	4.597
411	L626	2.959	316.191	0.879	0	0	0	1	1	0	0	0	0	3	1	0	4.643
412	L628	2.959	316.191	0.922	0	0	1	1	1	0	0	0	0	3	1	0	4.643
413	L630	3.260	316.191	0.912	0	0	0	1	2	0	0	0	0	3	1	0	4.643
414	L632	3.585	344.643	0.89	0	0	0	1	1	0	0	0	0	3	1	0	4.778
415	L634	3.602	375.827	0.889	0	0	0	1	1	0	0	0	0	3	1	0	5.013
416	L636	3.328	408.996	0.89	0	0	0	1	1	0	0	0	0	3	1	0	5.4
417	L642	4.292	516.572	0.891	0	0	0	1	1	0	0	0	0	3	1	0	7.6
418	L644	3.301	359.95	0.815	0	0	0	1	2	0	0	0	0	3	1	0	4.879
419	L646	2.996	359.95	0.907	0	0	0	1	1	0	0	0	0	3	1	0	4.879
420	L648	2.991	359.95	0.907	0	0	0	1	1	0	0	0	0	3	1	0	4.879
421	L650	2.991	359.95	0.884	0	0	0	1	1	0	0	0	0	3	1	0	4.879
422	L652	5.038	227.224	1.562	0	0	0	0	1	0	1	0	0	0	0	0	2.536
423	L666	3.337	93.411	1.036	0	0	0	0	3	2	0	0	0	2	0	0	2.341
424	L667	3.033	93.411	1.036	0	0	0	0	2	1	0	0	0	0	0	0	2.341
425	L668	3.537	47.105	1.379	0	0	0	0	3	0	0	0	0	0	0	0	2.083
426	L673	3.835	389.431	0.829	0	0	1	0	4	2	0	0	0	0	0	0	6.81
427	L674	4.036	183.244	1.037	0	0	0	0	2	2	0	0	0	0	0	0	4.548
428	L677	4.033	0	1.273	0	0	0	0	0	0	0	0	0	5	0	0	4.679
429	L678	3.932	0	1.143	0	0	0	0	1	0	0	0	0	0	0	0	5.07
430	L679	3.654	344.671	0.923	0	0	0	0	3	0	0	0	0	0	1	0	8.848

431	L681	4.256	345.574	0.904	0	0	0	0	3	0	0	0	0	0	1	0	8.856
432	L683	4.271	344.671	0.827	0	0	0	0	3	0	0	0	0	0	1	0	8.848
433	L685	4.572	345.574	0.547	0	0	0	0	3	0	0	0	0	0	1	0	8.856
434	L687	4.302	383.209	0.796	0	0	0	0	3	0	0	0	0	0	1	0	9.544
435	L689	4.302	386.72	0.576	0	0	0	0	3	0	0	0	0	0	1	0	9.578
436	L691	6.291	131.457	1.641	0	0	0	0	3	0	1	0	0	0	0	0	2.609
437	L692	5.999	125.869	1.573	0	0	0	0	3	0	1	0	0	0	0	0	2.464
438	L694	6.307	201.575	1.51	0	0	0	0	2	0	1	0	0	0	0	0	2.797
439	L695	6.266	140.552	1.474	0	0	0	0	3	0	1	0	0	0	0	0	2.964
440	L696	3.472	0	0.525	0	0	0	0	1	0	0	0	0	0	0	0	6.873
441	L697	3.630	221.611	0.674	0	0	0	0	1	0	0	0	0	0	0	0	2.212
442	L698	3.329	223.217	0.66	0	0	0	0	1	0	0	0	0	0	0	0	2.246
443	L699	3.317	167.372	0.769	0	0	0	0	1	0	0	0	0	0	0	0	4.08
444	L700	3.673	196.364	0.716	0	0	0	0	0	0	0	0	0	3	0	0	4.132
445	L702	4.507	509.969	1.671	0	0	0	0	2	1	0	0	0	2	0	0	3.517
446	L703	4.133	451.398	0.699	0	0	0	0	0	0	0	0	0	2	0	0	3.943
447	L704	4.222	519.152	1.243	0	0	0	0	0	0	0	0	0	2	0	0	3.52
448	L705	4.355	264.316	0.86	0	0	0	0	1	0	0	0	0	2	0	0	4.739
449	L706	4.256	126.16	0.735	1	0	0	0	2	1	0	0	0	2	0	0	3.366
450	L707	3.532	161.844	1.474	0	0	0	0	1	1	0	0	0	0	1	0	3.331
451	L708	3.740	87.007	1.577	0	0	0	0	1	1	0	0	0	0	2	0	2.539
452	L709	3.542	144.168	1.169	0	0	0	0	0	0	0	0	0	0	1	0	2.286
453	L710	3.516	186.368	1.536	0	0	0	0	2	1	0	0	0	0	0	0	3.542
454	L712	3.465	144.168	1.175	0	0	0	0	1	0	0	0	0	0	0	0	2.286
455	L713	3.468	101.97	1.358	0	0	0	0	2	1	0	0	0	0	0	0	3.797
456	L714	3.401	77.655	1.373	0	0	0	0	2	1	0	0	0	0	0	0	2.129
457	L715	3.480	160.762	1.51	0	0	0	0	1	1	0	0	0	0	0	0	3.007
458	L716	3.459	95.851	1.454	0	0	0	0	1	1	0	0	0	0	0	0	3.194
459	L718	3.424	85.472	1.457	0	0	0	0	2	1	0	0	0	0	0	0	2.667

460	L721	3.454	148.777	1.555	0	0	0	0	1	1	0	0	0	0	1	0	2.777
461	L722	3.413	78.317	0.792	0	0	0	1	0	0	0	0	0	0	1	0	1.89
462	L724	3.539	104.851	1.258	0	0	0	1	2	1	0	0	0	2	1	0	3.84
463	L726	3.544	110.577	1.328	0	0	0	0	1	1	0	0	0	0	0	0	4.371
464	L727	3.489	85.472	1.405	0	0	0	0	1	1	0	0	0	0	0	0	2.667
465	L728	3.527	101.97	1.35	0	0	0	0	1	1	0	0	0	0	0	0	3.797
466	L729	3.546	110.349	1.338	0	0	0	0	2	1	0	0	0	5	0	0	4.277
467	L730	3.489	84.281	0.707	0	0	0	0	1	1	0	0	0	0	0	0	2.19
468	L731	3.489	83.777	0.764	0	0	0	0	0	0	0	0	0	0	0	0	1.89
469	L732	4.601	95.544	1.251	0	0	0	0	4	0	0	0	0	0	0	0	7.18
470	L733	4.036	106.353	1.227	0	0	0	0	4	0	0	0	0	0	0	0	8.178
471	L734	4.303	95.544	1.251	0	0	0	0	4	0	0	0	0	0	0	0	7.18
472	L735	3.784	120.475	1.161	0	0	0	0	3	0	0	0	0	3	0	0	7.765
473	L736	3.754	109.666	1.163	0	0	0	0	3	0	0	0	0	3	0	0	6.68
474	L737	3.752	109.666	1.163	0	0	0	0	3	0	0	0	0	3	0	0	6.68
475	L738	3.815	131.391	1.16	0	0	0	0	3	0	0	0	0	3	0	0	8.837
476	L739	3.977	91.677	1.685	0	0	0	0	3	0	0	0	0	0	0	0	7.069
477	L740	3.735	105.8	1.393	0	0	0	0	2	0	0	0	0	3	0	0	6.65
478	L741	3.732	105.8	1.393	0	0	0	0	2	0	0	0	0	3	0	0	6.65
479	L742	3.899	195.107	0.915	0	0	0	0	2	1	0	0	0	0	0	0	1.995
480	L747	4.077	161.708	0.802	0	0	0	0	0	0	0	0	0	0	0	0	4.552
481	L753	3.412	140.238	0.713	0	0	0	0	3	0	0	0	0	0	0	0	2.645
482	L760	4.002	261.892	1.226	0	0	0	0	3	1	0	0	0	0	0	0	2.465
483	L761	3.796	138.477	1.163	0	0	0	0	3	1	0	0	0	0	0	0	2.628
484	L762	3.990	93.102	1.009	0	0	0	0	3	1	0	0	0	0	0	0	1.603
485	L763	4.017	99.614	1.096	0	0	0	0	4	3	0	0	0	0	0	0	1.636
486	L764	5.344	785.333	1.103	0	0	0	0	9	3	0	0	0	0	0	0	9.005
487	L765	5.304	694.174	1.239	0	0	0	0	8	1	0	0	0	2	2	0	8.477
488	L766	5.304	686.837	1.208	0	0	0	0	8	1	0	0	0	2	2	0	8.436

489	L767	5.312	711.274	1.232	0	0	0	0	8	1	0	0	0	2	2	0	8.646
490	L768	5.017	962.13	1.252	0	0	0	0	8	1	0	0	0	2	2	0	8.742
491	L769	5.004	676.968	0.674	0	0	0	0	8	1	0	1	0	0	2	0	8.315
492	L770	5.007	935.338	1.23	0	0	0	0	8	1	0	0	0	0	2	0	8.564
493	L771	5.308	901.408	1.211	0	0	0	0	8	1	0	0	0	0	2	0	8.513
494	L772	5.021	988.667	1.259	0	0	0	0	8	1	0	0	0	2	2	0	8.942
495	L773	5.327	745.414	1.135	0	0	1	0	8	1	0	0	0	0	2	0	9.021
496	L774	5.029	1015.035	1.141	0	0	1	0	8	1	0	0	0	0	2	0	9.163
497	L775	5.346	1256.719	1.167	0	0	0	0	8	1	0	0	0	0	2	0	8.962
498	L776	5.069	952.21	0.792	0	0	0	0	8	1	0	0	0	0	2	0	8.63
499	L777	3.758	114.094	1.05	0	0	0	0	3	1	0	0	0	0	0	0	1.689
500	L778	4.338	105.488	1.064	0	0	0	0	4	1	0	0	0	0	0	0	1.542
501	L779	4.071	478.258	1.007	0	0	0	0	9	5	0	0	0	0	0	0	3.402
502	L780	4.035	435.848	0.972	0	0	0	0	8	2	0	0	0	0	0	0	3.235
503	L781	4.059	465.311	0.993	0	0	0	0	8	4	0	0	0	0	0	0	3.36
504	L782	4.046	452.364	0.98	0	0	0	0	7	2	0	0	0	0	0	0	3.313
505	L785	4.302	44.111	1.074	0	0	1	0	1	0	0	0	0	0	0	0	1.649
506	L787	4.841	245.174	1.014	0	0	0	0	5	2	0	0	0	0	1	0	5.351
507	L791	2.699	0	1.494	0	0	0	0	1	0	0	1	0	3	2	0	4.243
508	L793	2.699	56.913	1.723	0	0	0	1	2	0	0	1	0	3	3	0	4.786
509	L798	5.231	2187.71	1.141	0	0	0	0	16	6	0	0	0	0	0	0	3.734
510	L799	4.262	420.766	0.982	0	0	0	0	12	4	0	0	0	0	0	0	3.712
511	L800	4.704	565.688	0.962	0	0	0	0	20	7	0	0	0	0	0	0	4.697
512	L801	3.698	191.923	1.366	0	0	0	1	5	0	0	0	0	0	1	0	5.556
513	L802	4.304	171.163	0.688	0	0	0	1	5	0	0	0	0	0	1	0	4.987
514	L803	3.700	182.079	1.431	0	0	0	1	5	0	0	0	0	0	1	0	5.293
515	L804	4.300	170.25	0.9	0	0	0	1	5	0	0	0	0	0	1	0	4.965
516	L806	3.701	171.163	1.652	0	0	0	1	5	0	0	0	0	0	1	0	4.987
517	L807	4.004	199.158	1.31	0	0	0	1	5	0	0	0	0	0	1	0	5.518

518	L808	3.699	196.559	0.833	0	0	0	1	5	0	0	0	0	0	1	0	5.41
519	L810	4.824	217.207	1.218	0	0	0	1	4	0	0	0	0	0	1	0	5.909
520	L811	4.301	196.447	0.617	0	0	0	1	4	0	0	0	0	0	1	0	5.328
521	L812	3.699	207.363	1.261	0	0	0	1	4	0	0	0	0	0	1	0	5.638
522	L813	3.699	195.534	0.816	0	0	0	1	4	0	0	0	0	0	1	0	5.309
523	L814	3.699	224.381	0.824	0	0	0	1	4	0	0	0	0	0	1	0	5.863
524	L815	3.699	196.447	1.458	0	0	0	1	4	0	0	0	0	0	1	0	5.328
525	L816	3.699	224.442	1.18	0	0	0	1	4	0	0	0	0	0	1	0	5.914
526	L817	3.699	221.843	0.741	0	0	0	1	4	0	0	0	0	0	1	0	5.823
527	L818	3.699	246.326	0.6	0	0	0	1	4	0	0	0	0	0	1	0	5.966
528	L819	3.699	223.611	0.479	0	0	0	1	4	0	0	0	0	0	1	0	5.818
529	L820	3.699	204.452	0.505	0	0	0	1	4	0	0	0	0	0	1	0	5.47
530	L821	5.177	261.66	0.964	0	0	0	0	23	7	0	0	0	2	0	0	8.788
531	L822	5.165	0	0.933	0	0	0	0	23	7	0	0	0	2	0	0	8.89
532	L824	5.473	258.39	0.969	0	0	0	0	23	8	0	0	0	2	0	0	8.873
533	L825	5.779	871.895	0.999	0	0	0	0	23	7	0	0	0	0	0	0	9.173
534	L826	5.789	959.82	0.985	0	0	0	0	23	7	0	0	0	0	0	0	9.683
535	L827	5.493	982.76	0.982	0	0	0	0	24	7	0	0	0	0	0	0	10.067
536	L829	6.090	993.858	0.977	0	0	0	0	23	7	0	0	0	0	0	0	9.702
537	L830	5.795	1013.268	0.923	0	0	0	0	23	7	0	1	0	0	1	0	9.988
538	L831	6.099	1014.933	0.972	0	0	0	0	23	7	0	0	0	0	0	0	10.099
539	L832	6.400	981.397	1.005	0	0	0	0	23	7	0	0	0	0	0	0	10.085
540	L833	5.799	981.397	0.95	0	0	0	0	23	7	0	0	0	0	0	0	10.085
541	L834	5.498	982.726	1	0	0	0	0	23	7	0	0	0	0	0	0	10.07
542	L835	5.498	984.537	1.001	0	0	0	0	23	7	0	0	0	0	0	0	10.049
543	L836	6.102	970.27	0.741	0	0	0	0	23	8	0	0	0	0	0	0	9.885
544	L837	5.500	970.27	0.737	0	0	0	0	23	7	0	0	0	0	1	0	9.885
545	L838	5.508	995.873	0.745	0	0	0	0	23	7	0	0	0	0	0	0	10.351
546	L839	6.090	959.82	0.989	0	0	0	0	23	7	0	0	0	0	1	0	9.683

547	L840	5.488	959.82	0.999	0	0	0	0	23	7	0	0	0	0	1	0	9.683
548	L841	5.800	985.422	0.97	0	0	0	0	23	7	0	0	0	0	1	0	10.169
549	L842	5.499	982.726	1.005	0	0	0	0	23	7	0	1	0	0	1	0	10.07
550	L843	5.789	959.82	1.004	0	0	0	0	23	7	0	0	0	0	2	0	9.683
551	L844	6.102	969.788	0.649	0	0	0	0	23	7	0	0	0	0	1	0	9.918
552	L845	6.403	970.952	0.649	0	0	0	0	23	7	0	0	0	0	1	0	9.924
553	L846	6.102	972.377	0.618	0	0	0	0	23	7	0	0	0	0	1	0	9.926
554	L848	4.402	422.252	0.957	0	0	0	0	8	1	0	0	0	0	3	0	8.058
555	L865	4.703	0	0.888	0	0	0	0	4	2	0	0	0	0	1	0	4.211
556	L867	4.701	0	0.888	0	0	0	0	4	2	0	0	0	0	1	0	4.211
557	L868	3.877	0	0.512	0	0	0	0	4	2	0	0	0	0	1	0	4.414
558	L884	4.320	80.865	0.655	0	0	0	0	2	0	0	0	0	2	0	0	4.128
559	L887	5.854	194.915	1.031	0	0	0	0	9	3	1	0	0	0	1	0	7.674
560	L888	4.051	0	0.888	0	0	0	0	5	2	0	0	0	0	1	0	4.184
561	L889	5.087	131.613	0.895	0	0	0	0	6	1	1	0	0	0	1	0	5.291
562	L890	6.438	197.426	0.837	0	0	0	0	8	3	1	0	0	0	1	0	8.301
563	L893	4.622	176.894	0.978	0	0	0	0	8	2	1	0	0	0	2	0	7.072
564	L894	5.199	175.043	0.969	0	0	0	0	9	2	1	0	0	0	1	0	6.911
565	L895	6.016	141.376	0.729	0	0	0	0	6	2	1	0	0	0	1	0	5.499
566	L896	4.478	0	0.749	0	0	0	0	5	2	0	0	0	3	1	0	5.159
567	L897	5.099	137.51	0.711	0	0	0	0	5	2	1	0	0	0	1	0	5.369
568	L898	4.547	154.742	0.868	0	0	0	0	5	2	1	0	0	0	1	0	6.638
569	L899	4.822	148.14	0.75	0	0	0	0	5	2	1	0	0	0	1	0	6.054
570	L900	6.027	145.567	0.684	0	0	0	0	6	2	1	0	0	0	1	0	5.479
571	L902	5.738	149.995	0.65	0	0	0	0	6	2	1	0	0	0	1	0	5.466
572	L904	5.749	154.424	0.622	0	0	0	0	6	2	1	0	0	0	1	0	5.455
573	L906	5.747	153.059	0.622	0	0	0	0	6	2	1	0	0	0	1	0	5.455
574	L908	5.435	149.995	0.65	0	0	0	0	6	2	1	0	0	0	1	0	5.466
575	L910	5.774	325.241	0.564	0	0	0	0	6	2	1	0	0	0	1	0	5.461

576	L912	5.779	159.371	0.825	0	0	0	0	6	2	1	0	0	0	1	0	5.453
577	L914	5.779	159.371	0.831	0	0	0	0	6	2	1	0	0	0	2	0	5.453
578	L916	6.051	154.424	0.657	0	0	0	0	7	2	1	0	0	0	1	0	5.455
579	L918	5.761	159.035	0.631	0	0	0	0	6	2	1	0	0	0	1	0	5.454
580	L920	6.061	159.035	0.619	0	0	0	0	7	2	1	0	0	0	1	0	5.454
581	L922	6.040	149.995	0.669	0	0	0	0	7	2	1	0	0	0	1	0	5.466
582	L924	6.051	154.606	0.639	0	0	1	0	6	2	1	0	0	0	1	0	5.464
583	L926	5.470	164.118	0.605	0	0	1	0	6	2	1	0	0	0	1	0	5.503
584	L928	6.063	154.606	0.901	0	0	0	0	6	2	1	0	0	0	1	0	5.464
585	L930	5.783	164.118	0.848	0	0	0	0	6	2	1	0	0	0	1	0	5.503
586	L932	5.485	163.829	0.856	0	0	0	0	6	2	1	0	0	0	1	0	5.478
587	L934	5.754	149.388	0.768	0	0	0	0	6	2	1	0	0	0	1	0	5.327
588	L936	5.426	145.567	0.698	0	0	0	0	6	2	1	0	0	0	2	0	5.479
589	L938	6.076	158.246	0.695	0	0	0	0	6	2	1	0	0	0	1	0	5.312
590	L939	5.448	154.424	0.634	0	0	0	0	6	2	1	0	0	0	2	0	5.455
591	L942	5.275	380.395	0.823	0	0	0	0	8	2	0	0	0	0	1	0	7.476
592	L944	4.570	108.754	0.753	0	0	0	0	1	0	0	0	0	2	0	0	3.426

Table S3. Average values for each molecular descriptor

	Variable	Exp1	Exp2	Exp3	Exp4	Exp5	Exp6	Exp7	Exp8	Exp9	Exp10	Average	Est. Dev.
	Intercept	4.328	4.134	4.336	4.338	4.358	4.299	4.256	4.239	4.286	4.342	4.292	0.005
1	D/Dr06	0.000	0.001	0.000	0.001	0.001	0.001	0.001	0.001	0.001	0.001	0.001	0.000
2	GATS6m	-0.479	-0.380	-0.487	-0.455	-0.426	-0.404	-0.317	-0.511	-0.405	-0.513	-0.438	0.004
3	nArCOOH	0.480	0.509	0.514	0.475	0.506	0.624	0.649	0.521	0.475	0.537	0.529	0.004
4	nRCONH2	1.147	1.267	1.320	1.267	1.185	1.198	1.183	1.311	1.355	1.252	1.249	0.005
5	nROR	0.300	0.401	0.381	0.341	0.334	0.326	0.303	0.298	0.305	0.347	0.334	0.001
6	nImidazoles	-0.430	-0.261	-0.493	-0.405	-0.464	-0.470	-0.447	-0.437	-0.533	-0.353	-0.429	0.006
7	nHDon	0.094	0.117	0.126	0.111	0.119	0.121	0.132	0.111	0.108	0.110	0.115	0.000
8	nHBonds	-0.144	-0.209	-0.231	-0.203	-0.213	-0.219	-0.232	-0.202	-0.188	-0.195	-0.204	0.001
9	C-018	1.245	1.322	1.267	1.274	1.258	1.405	1.213	1.188	1.156	1.242	1.257	0.005
10	C-029	0.414	0.480	0.523	0.469	0.451	0.516	0.480	0.464	0.456	0.506	0.476	0.001
11	C-032	1.109	1.186	1.116	1.085	1.116	1.086	1.138	1.194	1.152	1.304	1.149	0.004
12	H-051	-0.128	-0.090	-0.099	-0.144	-0.102	-0.101	-0.089	-0.088	-0.086	-0.126	-0.105	0.000
13	N-075	-0.133	-0.212	-0.200	-0.184	-0.206	-0.187	-0.193	-0.168	-0.168	-0.212	-0.186	0.001
14	N-079	-0.512	-0.589	-0.558	-0.581	-0.536	-0.543	-0.614	-0.525	-0.545	-0.544	-0.555	0.001
15	TI2	0.037	0.030	0.030	0.022	0.013	0.009	0.004	0.047	0.024	0.035	0.025	0.000

Table S4. Statistical parameters for the evaluation of the predictive power.

Exp.	Q^2	r^2	$\frac{r^2 - r_0^2}{r^2}$	k	k'	$\frac{r^2 - r'_0{}^2}{r^2}$	$\overline{r_m^2}$	$\lvert r_0^2 - r'_0{}^2 \rvert$	Δr_m^2
1	0.664	0.680	0.000	0.999	0.991	0.218	0.548	0.148	0.261
2	0.674	0.676	0.001	1.004	0.986	0.183	0.549	0.124	0.221
3	0.699	0.682	0.001	0.996	0.994	0.166	0.557	0.113	0.209
4	0.674	0.680	0.001	1.002	0.988	0.174	0.554	0.119	0.216
5	0.668	0.682	0.000	0.997	0.993	0.211	0.552	0.144	0.259
6	0.679	0.679	0.002	0.996	0.994	0.162	0.554	0.110	0.199
7	0.674	0.678	0.000	0.999	0.991	0.205	0.549	0.139	0.247
8	0.685	0.678	0.001	1.002	0.988	0.189	0.550	0.128	0.230
9	0.684	0.678	0.000	0.998	0.992	0.195	0.549	0.132	0.236
10	0.686	0.681	0.001	0.998	0.992	0.171	0.555	0.117	0.214
Average	0.679	0.679	0.001	0.999	0.991	0.187	0.552	0.127	0.229
Est. Dev.	0.000	0.000	0.000	0.000	0.000	0.000	0.000	0.000	0.000

$$r^2 = \frac{[\Sigma(Y_{obs} - \overline{Y_{obs}})(Y_{pred} - \overline{Y_{pred}})]^2}{\Sigma(Y_{pred} - \overline{Y_{pred}})^2 * \Sigma(Y_{obs} - \overline{Y_{obs}})^2} \quad \text{(S1)}$$

$$r_0^2 = 1 - \frac{[\Sigma(Y_{obs} - (k * Y_{pred}))]^2}{\Sigma(Y_{obs} - \overline{Y_{obs}})^2} \quad \text{(S2)}$$

$$r'_0{}^2 = 1 - \frac{[\Sigma(Y_{pred} - (k' * Y_{obs}))]^2}{\Sigma(Y_{pred} - \overline{Y_{pred}})^2} \quad \text{(S3)}$$

$$k = \frac{\Sigma(Y_{obs} * Y_{pred})}{\Sigma(Y_{pred})^2} \quad \text{(S4)}$$

$$k' = \frac{\Sigma(Y_{obs} * Y_{pred})}{\Sigma(Y_{obs})^2} \quad \text{(S5)}$$

Table S5. List of molecules used for the validation tests sets, their calculated molecular descriptors, and predicted *p*MIC values. Molecules were obtained as follows: (a) 1 to 10 from reference [8]; (b) 11 to 32 from reference [9]; (c) 33 to 39 from reference [10]; 40 to 45 from reference; 46 and 47 from reference [11]; 48 to 76 from reference [12]; and 77 to 98 from reference [13].

No.	MolID	*p*MIC	MW	D/Dr06	GATS6m	nArCOOH	nRCONH2	nROR	nImidazoles	nHDon	nHBonds	C-018	C-029	C-032	H-051	N-075	N-079	TI2	*p*MIC$_{pred}$
1	P001	5.040	411.49	292.916	0.831	0	0	0	0	1	0	0	0	0	0	0	0	2.81	4.280
2	P002	4.103	380.52	264.61	1.416	0	0	0	0	1	0	0	0	0	0	0	0	2.594	4.002
3	P003	3.807	384.48	264.61	0.779	0	0	0	0	1	0	0	0	0	0	0	0	2.594	4.281
4	P004	4.172	445.38	264.61	0.583	0	0	0	0	1	0	0	0	0	0	0	0	2.594	4.367
5	P005	4.730	402.47	274.785	1.073	0	0	0	0	1	0	0	0	0	0	0	0	2.603	4.159
6	P006	4.760	431.35	255.939	0.6	0	0	0	0	1	0	0	0	0	0	0	0	2.672	4.357
7	P007	4.803	476.35	296.928	0.634	0	0	0	0	1	0	0	0	0	0	0	0	2.811	4.369
8	P008	3.747	335.39	164.429	0.765	0	0	0	0	1	0	0	0	0	0	0	0	2.736	4.234
9	P009	4.938	324.83	142.277	0.739	0	0	0	0	1	0	0	0	0	0	0	0	2.268	4.221
10	P010	4.037	326.37	150.278	1.223	0	0	0	0	1	0	0	0	0	0	0	0	2.339	4.015
11	P011	4.648	355.44	86.758	0.844	0	0	0	0	5	1	0	0	0	0	1	0	4.245	4.261
12	P012	4.393	395.51	100.554	0.77	0	0	0	0	4	1	0	0	0	0	1	0	4.847	4.202
13	P013	4.090	393.49	100.554	0.77	0	0	0	0	4	1	0	0	0	0	1	0	4.847	4.202
14	P014	5.068	467.96	235.976	0.85	0	0	0	0	4	1	0	1	1	0	3	0	5.555	5.513
15	P015	4.438	438.56	106.005	0.844	0	0	0	0	4	1	0	0	0	0	2	0	5.198	3.995
16	P016	4.453	453.58	110.383	0.817	0	0	0	0	4	1	0	0	0	3	3	0	5.389	3.511
17	P017	4.135	436.52	110.383	0.724	0	0	0	0	4	1	0	0	0	3	2	0	5.389	3.738
18	P018	4.450	450.55	113.825	0.705	0	0	0	0	4	1	0	0	0	3	2	0	5.534	3.752
19	P019	4.734	433.52	228.131	0.714	0	0	0	0	4	1	0	0	1	0	3	0	5.382	5.088
20	P020	4.146	447.55	235.976	0.693	0	0	0	0	4	1	0	0	1	3	3	0	5.555	4.790
21	P021	4.161	463.55	246.209	0.857	0	0	0	0	4	1	0	0	1	0	3	0	5.756	5.045
22	P022	4.159	461.58	243.821	0.677	0	0	0	0	4	1	0	0	1	6	3	0	5.701	4.489
23	P023	3.559	463.55	244.305	0.88	0	0	0	0	4	1	0	2	0	0	3	0	5.735	4.837

24	P024	4.790	493.58	260.48	1.025	0	0	0	0	4	1	0	2	1	0	3	0	5.974	5.937
25	P025	4.432	432.53	228.131	0.704	0	0	0	0	4	1	0	1	0	0	2	0	5.382	4.606
26	P026	4.462	463.55	242.713	0.696	0	0	0	0	4	1	0	2	0	0	3	0	5.675	4.915
27	P027	3.860	463.55	246.209	0.857	0	0	0	0	4	1	0	2	0	0	3	0	5.756	4.849
28	P028	4.664	369.47	92.854	0.837	0	0	0	0	5	1	0	0	0	0	1	0	4.935	4.285
29	P029	5.885	383.5	98.843	0.918	0	0	0	0	5	1	0	0	0	0	1	0	5.611	4.270
30	P030	4.094	397.48	100.409	0.794	0	0	0	0	4	1	0	0	0	3	1	0	4.809	3.874
31	P031	4.997	397.49	100.409	0.794	0	0	0	0	7	2	0	0	0	0	1	0	4.809	4.331
32	P032	6.264	459.55	249.294	0.897	0	0	0	0	4	1	0	0	0	0	1	0	5.818	4.255
33	P033	4.549	566.9	644.687	1.162	0	0	0	0	0	0	0	2	0	0	2	2	8.161	3.824
34	P034	4.590	623.02	719.33	1.178	0	0	0	0	0	0	0	2	0	0	2	2	9.329	3.889
35	P035	4.549	566.9	704.755	1.462	0	0	0	0	0	0	0	0	0	0	2	2	8.716	2.789
36	P036	5.192	623.02	791.073	1.465	0	0	0	0	0	0	0	0	0	0	2	2	9.958	2.868
37	P037	4.561	582.9	730.69	1.049	0	0	0	0	0	0	0	0	0	0	2	2	9.287	2.999
38	P038	4.601	639.02	817.152	1.05	0	0	0	0	0	0	0	0	0	0	2	2	10.507	3.079
39	P039	4.620	667.08	860.738	1.051	0	0	0	0	0	0	0	0	0	0	2	2	11.148	3.119
40	P040	5.256	564.92	94.221	1.765	0	0	0	0	4	1	0	0	0	3	0	0	6.233	3.667
41	P041	5.510	566.94	94.221	1.765	0	0	0	0	5	1	0	0	0	0	0	0	6.233	4.098
42	P042	5.338	681.11	126.495	1.677	0	0	0	0	8	2	0	0	0	0	0	0	8.446	4.351
43	P043	5.300	624	109.95	1.73	0	0	0	0	6	2	0	0	0	0	0	0	7.361	4.062
44	P044	5.290	610.02	106.016	1.734	0	0	0	0	6	1	0	0	0	0	0	0	7.103	4.255
45	P045	6.171	444.48	221.13	1.164	0	0	0	0	7	4	0	0	0	0	0	0	2.026	3.948
46	P046	3.872	372.46	230.709	1.935	0	0	0	1	2	0	0	0	0	5	1	0	6.505	2.825
47	P047	3.946	441.34	251.358	1.051	0	0	0	1	2	1	0	0	0	5	1	0	6.718	3.026
48	P048	3.421	337.39	163.964	1.044	0	0	1	0	1	1	0	0	0	3	0	0	3.901	3.954
49	P049	3.496	401.44	317.333	1.064	0	0	1	0	1	1	0	0	0	0	2	0	5.295	4.011
50	P050	3.591	499.4	215.396	0.517	0	0	1	0	1	1	0	0	0	3	1	0	5.496	4.068
51	P051	3.501	405.44	208.358	1.137	0	0	1	0	2	2	0	0	0	2	3	0	5.488	3.436
52	P052	3.545	448.46	234.275	0.874	0	0	1	1	1	1	0	1	0	0	1	0	5.923	4.295

53	P053	3.579	485.37	207.282	0.517	0	0	1	0	1	1	0	0	0	0	1	0	5.303	4.374
54	P054	3.517	420.51	206.103	0.876	0	0	1	0	1	0	0	0	0	3	1	0	5.277	4.103
55	P055	3.502	406.48	197.99	0.879	0	0	1	0	1	0	0	0	0	0	1	0	5.044	4.407
56	P056	3.484	390.41	197.99	1.033	0	0	1	0	1	0	0	0	0	0	1	0	5.044	4.340
57	P057	3.501	405.49	197.99	0.869	0	0	1	0	1	0	0	0	0	0	0	0	5.044	4.598
58	P058	3.501	405.49	197.99	0.629	0	0	1	0	1	0	0	0	0	0	0	0	5.044	4.703
59	P059	3.482	388.44	197.99	1.104	0	0	1	0	2	0	0	0	0	0	0	0	5.044	4.610
60	P060	3.494	399.46	317.333	1.076	0	0	1	0	1	0	0	0	0	0	0	0	5.295	4.582
61	P061	3.524	427.52	348.902	1.082	0	0	1	0	1	0	0	0	0	2	0	0	6.042	4.405
62	P062	3.537	440.47	329.53	1.099	0	0	1	0	1	1	0	0	0	0	1	0	5.933	4.205
63	P063	3.535	438.5	329.53	1.135	0	0	1	0	2	1	0	0	0	0	0	0	5.933	4.490
64	P064	3.559	463.55	445.155	1.182	0	0	1	0	1	0	0	0	0	2	0	0	6.588	4.430
65	P065	3.505	409.53	197.99	0.869	0	0	1	0	1	0	0	0	0	0	0	0	5.044	4.598
66	P066	3.487	392.48	197.99	1.104	0	0	1	0	2	1	0	0	0	1	0	0	5.044	4.301
67	P067	3.502	406.51	207.282	1.096	0	0	1	0	1	1	0	0	0	1	0	0	5.303	4.202
68	P068	3.483	389.47	197.99	1.083	0	0	1	0	1	0	0	0	0	1	0	0	5.044	4.399
69	P069	3.439	351.42	173.546	1.071	0	0	1	0	1	1	0	0	0	2	0	0	4.201	4.061
70	P070	3.456	365.45	183.284	1.06	0	0	1	0	1	1	0	0	0	2	0	0	4.538	4.079
71	P071	3.456	365.45	183.128	1.098	0	0	1	0	1	1	0	0	0	1	0	0	4.488	4.167
72	P072	3.453	363.43	181.84	1.098	0	0	1	0	1	1	0	0	0	1	0	0	4.488	4.166
73	P073	3.470	377.46	190.459	1.09	0	0	1	0	1	1	0	0	0	1	0	0	4.774	4.182
74	P074	3.440	352.41	173.546	1.041	0	0	1	0	3	1	0	0	0	0	0	0	4.201	4.514
75	P075	3.457	366.44	183.284	1.087	0	0	1	0	3	1	0	0	0	2	0	0	4.538	4.297
76	P076	3.473	380.47	193.158	1.075	0	0	1	0	3	1	0	0	0	2	0	0	4.909	4.317
77	P077	6.006	506.57	484.937	0.935	0	0	0	0	1	0	0	4	0	0	2	0	6.202	5.960
78	P078	7.228	506.57	484.937	0.989	0	0	0	0	2	0	0	4	0	0	2	0	6.202	6.051
79	P079	5.705	506.57	484.937	0.953	0	0	0	0	2	0	0	2	0	0	2	0	6.202	5.115
80	P080	6.927	507.56	484.937	0.988	0	0	0	0	2	0	0	4	0	0	2	0	6.202	6.051
81	P081	6.926	505.58	484.937	0.973	0	0	0	0	2	0	0	2	0	0	1	0	6.202	5.292

82	P082	6.626	507.56	484.937	0.971	0	0	0	0	2	0	0	4	0	0	3	0	6.202	5.872
83	P083	6.006	506.57	484.937	0.936	0	0	0	0	2	0	0	3	0	0	2	0	6.202	5.598
84	P084	4.200	507.56	484.937	0.953	0	0	0	0	2	0	0	3	1	0	3	0	6.202	6.553
85	P085	3.909	519.56	501.962	1.158	1	0	0	0	3	0	0	2	0	0	1	0	6.347	5.868
86	P086	4.796	500.56	485.563	0.984	0	0	0	0	2	0	0	2	0	0	1	0	6.213	5.288
87	P087	6.302	500.56	484.937	1.061	0	0	0	0	2	0	0	2	0	0	1	0	6.202	5.254
88	P088	5.700	501.55	484.937	1.036	0	0	0	0	2	0	0	2	0	0	2	0	6.202	5.078
89	P089	7.217	494.53	467.912	1.022	0	0	0	0	2	0	0	2	0	0	1	0	6.049	5.257
90	P090	7.542	522.64	484.937	0.863	0	0	0	0	2	0	0	4	0	0	2	0	6.202	6.106
91	P091	6.635	517.62	484.937	0.865	0	0	0	0	2	0	0	2	0	0	2	0	6.202	5.153
92	P092	5.705	507.56	484.937	0.953	0	0	0	0	2	0	0	3	1	0	3	0	6.202	6.553
93	P093	5.728	534.63	519.047	0.981	0	0	0	0	2	0	0	4	0	0	2	0	6.359	6.078
94	P094	6.938	520.6	513.191	0.915	0	0	0	0	2	0	0	4	0	0	2	0	6.896	6.116
95	P095	6.627	508.56	495.866	1.026	0	0	0	0	2	0	0	2	0	0	1	0	6.731	5.288
96	P096	6.632	514.59	513.191	1.02	0	0	0	0	2	0	0	2	0	0	1	0	6.896	5.305
97	P097	6.018	520.6	513.191	0.951	0	0	0	0	2	0	0	2	0	0	2	0	6.896	5.149
98	P098	5.411	515.58	513.191	1.033	0	0	0	0	2	0	0	2	0	0	2	0	6.896	5.113

Table S6. List of molecules from the BIOFACQUIM database, their calculated molecular descriptors, and predicted *p*MIC values. The C-029, C-032 and N-079 were omitted as all of their values were zero. Data were obtained from the reference [14].

No.	MolID	MW	D/Dr06	GATS6m	nArCOOH	nRCONH2	nROR	nImidazoles	nHDon	nHBonds	C-018	H-051	N-075	TI2	*p*MIC
1	FQNP001	344.34	197.427	0.893	0	0	1	0	2	2	0	0	0	2.635	4.235
2	FQNP002	248.35	60.75	1.7	0	0	0	0	1	0	0	0	0	1.427	3.732
3	FQNP007	170.18	31.526	0	0	0	2	0	1	1	0	0	0	1.24	4.919
4	FQNP008	168.16	37.924	2.063	0	0	1	0	1	0	0	0	0	1.27	3.890
5	FQNP009	184.16	41.79	1.95	0	0	1	0	2	1	0	0	0	1.173	3.850
6	FQNP010	206.64	41.79	6.677	0	0	1	0	2	2	0	0	0	1.221	1.578
7	FQNP011	167.18	37.924	2.467	0	0	0	0	2	0	0	0	0	1.27	3.494
8	FQNP012	263.32	124.927	1.138	0	0	1	0	3	1	0	0	0	2.766	4.408
9	FQNP013	299.4	155.266	1.021	0	0	1	0	1	1	0	0	0	3.893	4.275
10	FQNP014	642.6	905.967	0.827	0	0	0	0	4	4	0	0	0	6.373	4.251
11	FQNP015	468.6	238.283	1.081	0	0	2	0	4	2	0	5	0	3.682	4.238
12	FQNP016	508.62	260.667	0.993	0	0	2	0	4	3	0	0	0	4.062	4.623
13	FQNP017	538.65	278.539	0.977	0	0	3	0	4	3	0	0	0	4.371	4.981
14	FQNP018	538.65	279.997	0.977	0	0	3	0	4	3	0	0	0	4.393	4.982
15	FQNP019	492.62	252.747	0.964	0	0	2	0	4	3	0	0	0	3.923	4.627
16	FQNP020	316.28	178.548	0.847	0	0	0	0	4	3	0	0	0	2.752	3.940
17	FQNP021	432.41	362.775	0.791	0	0	1	0	6	3	0	0	0	4.198	4.669
18	FQNP022	344.34	201.632	0.882	0	0	0	0	2	2	0	0	0	3.041	3.919
19	FQNP023	250.37	81.198	1.236	0	0	0	0	2	0	0	0	0	1.549	4.065
20	FQNP024	234.37	76.119	1.648	0	0	0	0	1	0	0	0	0	1.634	3.769
21	FQNP025	248.35	74.007	1.236	0	0	1	0	1	0	0	0	0	1.499	4.278
22	FQNP026	250.37	74.007	1.236	0	0	1	0	2	0	0	0	0	1.499	4.393
23	FQNP027	384.41	171.69	1.059	0	0	2	0	2	0	0	0	0	1.443	4.859
24	FQNP028	462.48	318.519	0.787	0	0	2	0	2	0	0	0	0	2.107	5.078

25	FQNP029	292.26	108.265	0.476	0	0	1	0	2	2	0	0	0	1.166	4.330
26	FQNP030	274.24	101.297	0.399	0	0	1	0	1	0	1	0	0	1.208	5.910
27	FQNP031	248.25	79.846	0.642	0	0	1	0	1	1	0	0	0	1.519	4.339
28	FQNP032	210.62	26.957	2.896	0	0	0	0	1	0	0	0	0	1.421	3.189
29	FQNP033	342.33	119.54	0.909	0	0	1	0	5	3	0	0	0	3.373	4.343
30	FQNP034	176.18	24.549	1.733	0	0	0	0	1	0	0	0	0	1.343	3.695
31	FQNP035	224.28	38.08	1.25	0	0	1	0	3	1	0	0	0	2.84	4.311
32	FQNP036	226.25	45.591	1.091	0	0	0	0	2	0	0	2	0	1.32	3.892
33	FQNP037	224.23	45.591	1.091	0	0	0	0	2	0	0	0	0	1.32	4.102
34	FQNP038	180.22	42.114	1.3	0	0	0	0	2	0	0	3	0	1.502	3.697
35	FQNP039	404.51	208.41	1.05	0	0	5	0	2	2	0	0	0	3.678	5.533
36	FQNP040	194.2	55.767	1.138	0	0	0	0	2	1	0	0	0	1.148	3.880
37	FQNP041	194.2	55.767	1.138	0	0	0	0	2	1	0	0	0	1.148	3.880
38	FQNP042	192.23	56.472	0.919	0	0	0	0	0	0	0	0	0	1.379	3.956
39	FQNP044	170.18	31.526	0	0	0	2	0	1	1	0	0	0	1.24	4.919
40	FQNP045	172.2	37.924	0	0	0	1	0	2	1	0	2	0	1.253	4.493
41	FQNP046	418.53	194.21	1.485	0	0	0	0	2	0	0	0	0	2.76	4.051
42	FQNP047	460.57	215.462	1.493	0	0	0	0	1	0	0	3	0	2.657	3.626
43	FQNP048	452.55	230.455	1.406	0	0	0	0	5	3	0	0	0	4.969	3.895
44	FQNP049	436.55	223.697	1.417	0	0	0	0	4	2	0	0	0	4.977	3.975
45	FQNP050	420.55	210.898	1.289	0	0	0	0	4	1	0	0	0	5.218	4.233
46	FQNP051	434.53	202.035	1.486	0	0	0	0	3	1	0	0	0	2.747	3.966
47	FQNP052	476.57	223.286	1.489	0	0	0	0	2	1	0	3	0	2.633	3.543
48	FQNP053	466.58	239.281	1.376	0	0	0	0	4	2	0	0	0	4.823	3.998
49	FQNP059	402.48	228.301	1.071	0	0	1	0	1	1	0	0	0	6.528	4.360
50	FQNP060	388.45	219.002	1.078	0	0	1	0	2	0	0	0	0	6.272	4.664
51	FQNP061	402.48	228.301	1.071	0	0	1	0	1	1	0	0	0	6.528	4.360
52	FQNP062	388.45	219.002	1.078	0	0	1	0	2	0	0	0	0	6.272	4.664
53	FQNP063	404.45	227.357	1.057	0	0	1	0	3	1	0	0	0	6.479	4.594

54	FQNP077	1167.41	1692.143	0.951	0	0	12	0	16	8	0	2	0	8.304	9.049
55	FQNP078	1195.47	1739.777	0.958	0	0	12	0	16	8	0	2	0	8.397	9.076
56	FQNP079	1341.63	2360.184	0.965	0	0	13	0	19	10	0	2	0	10.723	9.756
57	FQNP080	1690.16	2257.102	1.01	0	0	14	0	13	8	0	9	0	6.18	8.879
58	FQNP081	2473.43	6328.172	1.071	0	0	19	0	16	11	0	10	0	20.998	12.845
59	FQNP082	2449.3	7015.971	1.082	0	0	19	0	16	11	0	8	0	21.234	13.451
60	FQNP083	2445.37	6531.924	1.071	0	0	19	0	16	11	0	10	0	21.416	12.972
61	FQNP084	2501.49	6713.547	1.076	0	0	19	0	16	9	0	10	0	21.566	13.485
62	FQNP085	248.35	65.655	0.729	0	0	0	0	1	1	0	0	0	3.637	4.012
63	FQNP086	248.35	32.852	0	0	0	0	0	1	1	0	1	0	0.868	4.138
64	FQNP087	248.35	32.852	0	0	0	0	0	1	1	0	1	0	0.868	4.138
65	FQNP088	368.37	204.391	1.062	0	0	2	0	4	2	0	0	0	3.488	4.749
66	FQNP089	381.46	200.156	1.021	0	0	0	0	0	0	0	0	0	2.363	4.018
67	FQNP090	270.36	73.874	1.415	0	0	0	0	1	0	0	0	0	1.83	3.875
68	FQNP091	270.36	73.874	1.415	0	0	0	0	1	0	0	0	0	1.83	3.875
69	FQNP092	256.33	68.062	1.427	0	0	0	0	1	0	0	0	0	1.586	3.860
70	FQNP093	228.22	57.413	1.759	0	0	0	0	1	0	0	0	0	1.299	3.701
71	FQNP094	506.64	639.103	1.685	0	0	0	0	2	1	0	0	0	5.637	4.086
72	FQNP095	348.75	140.462	1.188	0	0	0	0	4	1	0	0	0	2.233	4.163
73	FQNP105	394.45	172.17	1.051	0	0	0	0	0	0	0	1	0	2.644	3.890
74	FQNP106	336.31	115.969	0.905	0	0	0	0	0	0	0	1	0	2.237	3.912
75	FQNP107	382.34	135.316	0.9	0	0	0	0	1	1	0	0	0	2.198	3.941
76	FQNP108	336.31	140.815	0.957	0	0	0	0	0	0	0	0	0	2.878	4.025
77	FQNP109	284.28	160.088	0.788	0	0	0	0	2	0	0	0	0	2.78	4.337
78	FQNP110	300.28	170.12	0.875	0	0	0	0	3	0	0	0	0	2.847	4.421
79	FQNP112	344.34	201.768	1.02	0	0	0	0	2	1	0	0	0	3.05	4.062
80	FQNP113	330.31	189.459	1.031	0	0	0	0	3	1	0	0	0	2.92	4.162
81	FQNP114	262.33	64.764	1.425	0	0	0	0	1	1	0	0	0	1.422	3.651
82	FQNP115	254.41	0	1	0	0	0	0	2	0	0	1	0	5.078	4.104

83	FQNP116	530.52	453.461	1.168	0	0	0	0	6	3	0	0	0	6.283	4.274
84	FQNP119	540.5	662.017	0.96	0	0	0	0	6	0	0	2	0	4.777	4.847
85	FQNP120	342.34	157.033	0.994	0	0	3	0	8	4	0	0	0	3.361	5.134
86	FQNP121	528.95	179.831	1.378	0	0	0	0	1	0	0	0	0	14.694	4.272
87	FQNP122	557.01	191.167	1.366	0	0	0	0	1	0	0	0	0	15.562	4.305
88	FQNP123	585.07	202.538	1.356	0	0	0	0	1	0	0	0	0	16.423	4.337
89	FQNP124	138.18	30.514	0	0	0	0	0	1	0	0	0	0	1.783	4.468
90	FQNP125	122.13	26.324	0	0	0	0	0	1	0	0	0	0	1.544	4.460
91	FQNP126	302.35	120.139	1.182	0	0	0	0	2	1	0	0	0	1.857	3.915
92	FQNP127	300.33	120.139	1.182	0	0	0	0	2	1	0	0	0	1.857	3.915
93	FQNP128	270.3	106.425	1.319	0	0	0	0	2	1	0	0	0	1.7	3.843
94	FQNP129	274.34	139.317	0.742	0	0	0	0	2	1	0	0	0	4.117	4.175
95	FQNP130	244.31	122.152	0.729	0	0	0	0	2	0	0	0	0	3.862	4.368
96	FQNP131	358.37	208.04	0.862	0	0	1	0	1	1	0	0	0	2.814	4.348
97	FQNP132	344.34	195.731	0.866	0	0	1	0	2	2	0	0	0	2.68	4.247
98	FQNP133	710.95	787.218	0.937	0	0	0	0	3	1	0	0	0	4.01	4.572
99	FQNP134	538.59	452.095	0.448	1	0	0	0	5	3	0	0	0	5.632	4.986
100	FQNP135	538.59	452.691	0.441	1	0	0	0	4	2	0	0	0	5.45	5.074
101	FQNP136	552.62	463.793	0.433	1	0	0	0	4	2	0	0	0	5.492	5.085
102	FQNP137	594.57	478.798	0.994	0	0	3	0	8	5	0	0	0	5.466	5.167
103	FQNP138	354.34	187.811	1.042	0	0	0	0	6	4	0	0	0	5.15	3.945
104	FQNP139	578.57	618.478	0.879	0	0	3	0	8	5	0	0	0	4.087	5.263
105	FQNP140	432.41	330.321	0.926	0	0	1	0	7	3	0	0	0	3.17	4.680
106	FQNP141	454.76	185.141	0.533	0	0	0	0	1	0	0	2	0	4.232	4.174
107	FQNP142	456.78	185.141	0.533	0	0	0	0	2	0	0	0	0	4.232	4.499
108	FQNP143	426.8	223.217	0.66	0	0	0	0	1	0	0	0	0	2.246	4.301
109	FQNP144	414.79	167.372	0.769	0	0	0	0	1	0	0	0	0	4.08	4.267
110	FQNP145	218.32	56.795	1.143	0	0	0	0	0	0	0	0	0	2.878	3.895
111	FQNP146	150.24	34.381	0	0	0	0	0	1	0	0	0	0	1.602	4.466

112	FQNP147	274.34	72.859	0.95	0	0	1	0	0	0	0	3	0	3.145	4.013
113	FQNP148	402.68	125.97	0.679	1	0	0	0	2	1	0	0	0	10.287	4.877
114	FQNP149	346.56	104.141	0.718	1	0	0	0	2	1	0	0	0	8.322	4.799
115	FQNP150	404.7	125.97	0.679	1	0	0	0	2	1	0	0	0	10.287	4.877
116	FQNP151	348.58	104.141	0.718	1	0	0	0	2	1	0	0	0	8.322	4.799
117	FQNP152	454.76	185.141	0.533	0	0	0	0	1	0	0	2	0	4.232	4.174
118	FQNP153	456.78	185.141	0.533	0	0	0	0	2	0	0	0	0	4.232	4.499
119	FQNP154	456.78	245.125	1.235	0	0	0	0	2	0	0	0	0	2.301	4.178
120	FQNP155	386.43	223.207	1.161	0	0	0	0	2	0	0	1	0	6.592	4.199
121	FQNP156	400.46	232.506	1.153	0	0	0	0	1	0	0	1	0	6.839	4.099
122	FQNP157	386.43	222.633	1.161	0	0	0	0	2	0	0	1	0	6.58	4.199
123	FQNP158	416.46	240.861	1.123	0	0	0	0	2	1	0	1	0	6.998	4.032
124	FQNP159	218.27	70.528	1.368	0	0	0	0	1	1	0	3	0	1.788	3.373
125	FQNP160	234.27	77.573	1.427	0	0	0	0	2	0	0	0	0	2.048	3.992
126	FQNP161	420.5	0	1.098	0	0	0	0	1	1	0	3	0	2.298	3.463
127	FQNP162	420.5	0	1.098	0	0	0	0	1	1	0	3	0	2.298	3.463
128	FQNP164	284.28	158.458	0.75	0	0	0	0	2	0	0	0	0	2.613	4.348
129	FQNP165	316.28	176.374	0.956	0	0	0	0	4	2	0	0	0	2.715	4.093
130	FQNP166	286.25	155.567	0.889	0	0	0	0	4	1	0	0	0	2.431	4.307
131	FQNP167	302.25	165.599	0.917	0	0	0	0	5	2	0	0	0	2.559	4.215
132	FQNP168	516.49	440.044	1.132	0	0	0	0	7	4	0	0	0	6.453	4.197
133	FQNP169	380.52	161.315	1.284	0	0	0	0	0	0	0	0	0	2.461	3.883
134	FQNP170	190.26	30.952	0.542	0	0	0	0	0	0	0	0	0	2.32	4.130
135	FQNP171	188.24	30.952	0.542	0	0	0	0	0	0	0	0	0	2.32	4.130
136	FQNP172	192.23	31.084	1.379	0	0	0	0	0	0	0	0	0	1.816	3.751
137	FQNP173	194.2	48.032	1.706	0	0	0	0	2	1	0	0	0	2.922	3.671
138	FQNP174	614.59	368.003	1.091	0	0	0	0	5	4	0	4	0	2.756	3.431
139	FQNP175	614.59	442.102	1.141	0	0	0	0	6	3	0	4	0	3.834	3.797
140	FQNP182	328.44	96.148	0.932	0	0	0	0	0	0	0	0	0	2.547	4.002

141	FQNP183	330.46	96.148	0.932	0	0	0	0	0	0	0	1	0	2.547	3.897
142	FQNP184	328.44	96.148	0.932	0	0	0	0	0	0	0	0	0	2.547	4.002
143	FQNP185	246.33	61.057	0.756	0	0	0	0	1	0	0	0	0	1.247	4.141
144	FQNP186	330.41	96.006	0.83	0	0	0	0	1	0	0	0	0	2.591	4.162
145	FQNP187	272.27	101.297	0.894	0	0	1	0	0	0	1	0	0	1.208	5.579
146	FQNP188	304.32	115.007	0.802	0	0	1	0	1	0	0	0	0	1.061	4.481
147	FQNP191	254.25	97.643	1.096	0	0	0	0	2	1	0	0	0	1.464	3.930
148	FQNP192	346.31	89.966	1.133	0	0	2	0	1	0	1	0	0	1.397	5.920
149	FQNP193	295.32	147.825	0.885	0	0	2	0	4	2	0	0	0	3.39	4.792
150	FQNP194	290.29	158.057	0.889	0	0	0	0	5	1	0	0	0	2.637	4.429
151	FQNP195	464.41	358.561	0.903	0	0	2	0	8	4	0	0	0	3.293	4.954
152	FQNP196	464.41	358.561	0.903	0	0	2	0	8	4	0	0	0	3.293	4.954
153	FQNP197	448.41	344.764	0.92	0	0	2	0	7	4	0	0	0	3.156	4.821
154	FQNP198	568.52	614.71	0.974	0	0	2	0	7	4	0	0	0	5.105	5.000
155	FQNP199	298.32	145.105	0.857	0	0	1	0	4	2	0	3	0	3.532	4.157
156	FQNP200	286.31	135.944	0.989	0	0	1	0	4	1	0	0	0	3.326	4.608
157	FQNP201	456.78	243.519	1.086	0	0	0	0	2	0	0	0	0	2.275	4.242
158	FQNP202	428.77	223.896	1.023	0	0	0	0	2	0	0	0	0	2.209	4.256
159	FQNP203	374.42	92.876	1.093	0	0	0	0	3	1	0	0	0	1.834	4.053
160	FQNP204	356.4	89.5	1.115	0	0	0	0	2	1	0	0	0	1.903	3.928
161	FQNP205	402.48	107.121	1.055	0	0	0	0	1	1	0	3	0	1.717	3.529
162	FQNP206	360.44	93.257	1.083	0	0	0	0	3	2	0	2	0	1.91	3.645
163	FQNP207	374.52	158.607	1.176	0	0	0	0	2	1	0	5	0	2.507	3.429
164	FQNP208	340.4	85.377	1.2	0	0	0	0	1	1	0	0	0	1.905	3.774
165	FQNP209	342.42	85.377	1.2	0	0	0	0	1	1	0	0	0	1.905	3.774
166	FQNP210	342.42	89.565	1.1	0	0	0	0	1	0	0	0	0	1.986	4.025
167	FQNP211	356.4	88.753	1.195	0	0	0	0	2	2	0	0	0	1.836	3.687
168	FQNP212	362.46	173.725	1.006	0	0	1	0	3	2	0	0	0	1.676	4.263
169	FQNP213	390.52	163.382	1.134	0	0	0	0	2	1	0	3	0	1.627	3.639

170	FQNP214	256.27	136.096	0.838	0	0	0	0	2	1	0	2	0	2.346	3.876
171	FQNP215	272.27	143.884	0.933	0	0	0	0	3	1	0	0	0	2.23	4.162
172	FQNP216	254.25	136.096	0.838	0	0	0	0	2	0	0	0	0	2.346	4.290
173	FQNP218	286.3	153.219	1	0	0	0	0	2	0	0	0	0	2.266	4.227
174	FQNP219	270.3	145.431	0.825	0	0	0	0	1	0	0	2	0	2.379	3.977
175	FQNP220	286.3	154.563	0.875	0	0	0	0	2	1	0	0	0	2.3	4.080
176	FQNP221	270.3	146.775	0.781	0	0	0	0	1	1	0	2	0	2.415	3.794
177	FQNP222	300.28	167.876	0.875	0	0	0	0	3	1	0	0	0	2.666	4.212
178	FQNP223	284.28	153.219	1	0	0	0	0	2	1	0	0	0	2.266	4.024
179	FQNP224	580.54	601.67	1.023	0	0	3	0	9	5	0	0	0	5.514	5.341
180	FQNP225	434.38	335.179	1.101	0	0	1	0	6	2	0	0	0	4.209	4.721
181	FQNP226	594.57	618.03	0.994	0	0	3	0	8	5	0	0	0	5.445	5.246
182	FQNP227	462.44	364.378	1.133	0	0	1	0	5	2	0	0	0	4.286	4.611
183	FQNP228	448.41	348.381	1.095	0	0	1	0	5	2	0	0	0	4.089	4.613
184	FQNP229	528.65	276.827	0.964	0	0	0	0	1	1	0	7	0	2.445	3.262
185	FQNP230	544.65	268.661	0.98	0	0	1	0	1	1	0	7	0	2.334	3.582
186	FQNP231	556.71	297.826	0.932	0	0	0	0	1	1	0	5	0	2.53	3.501
187	FQNP232	568.72	309.014	0.929	0	0	0	0	1	1	0	4	0	2.641	3.617
188	FQNP233	598.75	328.291	0.944	0	0	0	0	0	0	0	8	0	2.387	3.283
189	FQNP234	596.73	330.786	0.945	0	0	0	0	0	0	0	7	0	2.486	3.391
190	FQNP235	572.71	288.775	0.945	0	0	1	0	1	0	0	5	0	2.417	4.025
191	FQNP236	614.75	318.687	0.95	0	0	1	0	0	0	0	8	0	2.279	3.605
192	FQNP239	234.27	77.068	1.427	0	0	0	0	2	1	0	3	0	1.92	3.469
193	FQNP240	406.47	0	1.105	0	0	0	0	1	1	0	3	0	1.981	3.452
194	FQNP241	420.5	0	1.098	0	0	0	0	1	1	0	3	0	2.298	3.463
195	FQNP242	176.18	52.989	1.04	0	0	0	0	0	0	0	0	0	1.613	3.907
196	FQNP243	206.21	65.449	1.193	0	0	0	0	0	0	0	0	0	1.571	3.846
197	FQNP244	298.31	170.767	0.741	0	0	0	0	1	0	0	0	0	2.842	4.250
198	FQNP245	204.39	0	0	0	0	0	0	0	0	0	0	0	1.378	4.326

199	FQNP246	220.39	0	2.182	0	0	1	0	0	0	0	0	0	1.439	3.706
200	FQNP247	270.3	77.645	0.565	0	0	0	0	0	0	0	0	0	2.938	4.162
201	FQNP248	244.31	83.872	1.253	0	0	0	0	0	0	0	0	0	2.448	3.852
202	FQNP249	216.2	53.167	0.938	0	0	0	0	0	0	0	0	0	1.341	3.945
203	FQNP252	162.15	46.449	1.63	0	0	0	0	1	0	0	0	0	1.338	3.753
204	FQNP253	304.32	90.115	0.891	0	0	0	0	2	1	0	0	0	3.382	4.063
205	FQNP254	216.2	53.167	0.625	0	0	0	0	0	0	0	0	0	1.347	4.082
206	FQNP255	192.18	58.909	1.138	0	0	0	0	1	0	0	0	0	1.553	3.980
207	FQNP256	192.18	58.909	1.517	0	0	0	0	1	0	0	0	0	1.557	3.815
208	FQNP257	302.3	101.823	1.251	0	0	0	0	5	3	0	0	0	1.848	3.811
209	FQNP258	286.3	96.041	1.25	0	0	0	0	4	1	0	0	0	1.797	4.099
210	FQNP259	180.17	43.841	1.857	0	0	0	0	3	1	0	0	0	2.91	3.717
211	FQNP260	184.16	42.676	0.78	0	0	0	0	3	2	0	0	0	1.949	3.960
212	FQNP261	170.13	38.248	0	1	0	0	0	4	2	0	0	0	1.623	4.935
213	FQNP262	126.12	26	0	0	0	0	0	3	0	0	0	0	0.95	4.674
214	FQNP263	164.17	39.975	2.037	0	0	0	0	2	0	0	0	0	2.95	3.725
215	FQNP265	472.78	253.777	1.002	0	0	0	0	3	0	0	0	0	2.352	4.401
216	FQNP266	168.16	38.571	0	1	0	0	0	2	1	0	0	0	1.469	4.905
217	FQNP267	152.16	34.705	0	1	0	0	0	1	0	0	0	0	1.602	4.995
218	FQNP268	468.84	258.649	0.662	0	0	0	0	0	0	0	3	0	2.576	3.898
219	FQNP269	426.8	224.18	1.033	0	0	0	0	0	0	0	3	0	2.167	3.705
220	FQNP270	440.78	235.134	1.323	0	0	0	0	0	0	0	3	0	2.187	3.585
221	FQNP271	442.8	235.134	1.323	0	0	0	0	1	0	0	3	0	2.187	3.700
222	FQNP273	576.95	375.158	0.64	0	0	2	0	4	2	0	0	0	5.141	5.073
223	FQNP274	290.29	158.057	0.889	0	0	0	0	5	1	0	0	0	2.637	4.429
224	FQNP275	454.86	174.078	0.75	0	0	0	0	1	0	0	0	0	3.868	4.274
225	FQNP276	452.84	174.078	0.75	0	0	0	0	0	0	0	2	0	3.868	3.948
226	FQNP278	304.37	157.395	0.755	0	0	0	0	2	1	0	0	0	4.22	4.182
227	FQNP279	314.36	126.626	0.969	0	0	0	0	1	0	0	0	0	1.632	4.095

228	FQNP280	272.32	106.02	1.319	0	0	0	0	2	1	0	0	0	1.71	3.843
229	FQNP281	454.86	174.803	1.076	0	0	1	0	0	0	0	0	0	3.844	4.350
230	FQNP282	585.01	389.689	1.001	0	0	0	0	1	0	0	1	0	4.941	4.209
231	FQNP283	440.83	174.693	0.727	0	0	0	0	1	0	0	0	0	3.716	4.280
232	FQNP284	454.86	183.434	0.717	0	0	0	0	1	0	0	0	0	3.936	4.295
233	FQNP285	404.37	180.983	0.757	0	0	0	0	2	0	0	0	0	1.929	4.341
234	FQNP286	369.93	169.52	0.75	0	0	0	0	2	1	0	0	0	1.825	4.132
235	FQNP287	302.5	173.691	1.155	0	0	0	0	1	0	0	0	0	1.7	4.042
236	FQNP288	218.37	57.922	0.667	0	0	0	0	1	0	0	0	0	3.825	4.243
237	FQNP289	234.37	60.604	0.971	0	0	1	0	1	0	0	0	0	3.891	4.446
238	FQNP290	252.39	67.55	1.063	0	0	0	0	3	0	0	0	0	4.156	4.312
239	FQNP291	560.71	440.654	0.966	0	0	4	0	8	4	0	0	0	5.474	5.695
240	FQNP292	560.54	515.161	0.801	0	0	0	0	8	1	0	0	0	2.939	5.023
241	FQNP293	814.79	978.98	0.809	0	0	0	0	10	1	0	0	0	3.214	5.521
242	FQNP294	707.91	0	0.7	0	0	0	0	9	1	0	10	0	6.403	3.920
243	FQNP295	302.35	123.419	0.679	0	0	0	0	1	1	0	1	0	1.847	3.917
244	FQNP296	332.38	136.113	0.977	0	0	0	0	1	1	0	1	0	1.696	3.790
245	FQNP297	288.32	115.575	0.716	0	0	0	0	1	1	0	2	0	1.779	3.789
246	FQNP298	318.35	128.269	0.96	0	0	0	0	1	1	0	2	0	1.634	3.686
247	FQNP299	302.35	123.419	0.679	0	0	0	0	1	1	0	1	0	1.847	3.917
248	FQNP300	332.38	136.113	0.977	0	0	0	0	1	1	0	1	0	1.696	3.790
249	FQNP301	300.33	123.419	0.679	0	0	0	0	1	1	0	0	0	1.847	4.022
250	FQNP302	286.3	115.575	0.716	0	0	0	0	1	1	0	0	0	1.779	4.000
251	FQNP303	392.68	158.183	0.784	0	0	0	0	0	0	0	2	0	3.831	3.923
252	FQNP304	250.27	80.687	0.942	0	0	1	0	2	1	1	0	0	1.546	5.580
253	FQNP307	554.53	681.752	0.962	0	0	0	0	5	0	0	2	0	4.884	4.745
254	FQNP310	288.37	147.646	0.686	0	0	0	0	1	1	0	0	0	4.174	4.091
255	FQNP311	302.4	156.888	0.675	0	0	0	0	0	0	0	0	0	4.278	4.192
256	FQNP312	244.31	123.143	0.729	0	0	0	0	2	0	0	0	0	3.887	4.369

257	FQNP313	258.34	130.481	0.712	0	0	0	0	1	0	0	0	0	4.045	4.270
258	FQNP314	230.28	113.823	0.747	0	0	0	0	3	0	0	0	0	3.78	4.468
259	FQNP315	230.28	114.814	0.747	0	0	0	0	3	0	0	0	0	3.806	4.469
260	FQNP316	272.32	107.638	1.397	0	0	0	0	2	1	0	0	0	1.779	3.812
261	FQNP317	270.25	147.779	0.8	0	0	0	0	3	0	0	0	0	2.547	4.433
262	FQNP318	300.33	118.116	1.045	0	0	0	0	2	1	0	0	0	1.59	3.967
263	FQNP319	432.41	351.825	0.949	0	0	1	0	7	4	0	0	0	3.44	4.486
264	FQNP321	292.41	38.703	0.772	0	0	0	0	1	0	0	0	0	1.769	4.135
265	FQNP322	138.18	30.752	2.813	0	0	0	0	2	0	0	0	0	2.063	3.358
266	FQNP323	268.26	44.396	1.096	0	0	1	1	4	2	0	0	1	2.535	3.670
267	FQNP324	396.72	158.183	0.784	0	0	0	0	1	0	0	0	0	3.831	4.249
268	FQNP330	1151.41	1669.117	0.921	0	0	11	0	16	9	0	2	0	8.266	8.511
269	FQNP331	1179.47	1715.713	0.923	0	0	11	0	16	9	0	2	0	8.347	8.539
270	FQNP332	869.18	889.623	0.957	0	0	7	0	10	5	0	2	0	8.006	6.834
271	FQNP333	1035.28	1396.119	0.967	0	0	10	0	14	8	0	2	0	8.601	7.984
272	FQNP334	1165.44	1695.422	0.921	0	0	11	0	15	10	0	2	0	8.575	8.216
273	FQNP335	1193.5	1742.017	0.923	0	0	11	0	15	9	0	2	0	8.623	8.446
274	FQNP340	330.36	136.113	0.977	0	0	0	0	1	1	0	0	0	1.696	3.895
275	FQNP341	316.33	128.269	0.96	0	0	0	0	1	1	0	0	0	1.634	3.897
276	FQNP342	270.25	105.488	1.043	0	0	0	0	3	1	0	0	0	1.542	4.074
277	FQNP343	250.27	80.687	0.942	0	0	1	0	2	1	1	0	0	1.546	5.580
278	FQNP344	152.16	34.943	0	0	0	0	0	1	0	0	0	0	2.041	4.477
279	FQNP345	466.87	183.44	0.739	0	0	0	0	0	0	0	2	0	4.141	3.965
280	FQNP346	468.84	180.147	0.746	0	0	0	0	0	0	0	0	0	3.694	4.160
281	FQNP347	438.81	165.605	0.762	0	0	0	0	0	0	0	2	0	3.647	3.933
282	FQNP348	440.83	165.605	0.762	0	0	0	0	1	0	0	0	0	3.647	4.258
283	FQNP349	482.87	187.726	0.736	0	0	0	0	0	0	0	3	0	3.743	3.854
284	FQNP350	360.39	154.53	1.116	0	0	0	0	0	0	0	5	0	1.931	3.413
285	FQNP351	332.38	137.144	1.114	0	0	0	0	1	0	0	1	0	2.409	3.952

286	FQNP352	344.39	146.041	1.091	0	0	0	0	1	0	0	0	0	2.719	4.080
287	FQNP353	344.39	152.751	1.203	0	0	0	0	1	0	0	0	0	2.934	4.040
288	FQNP354	374.42	162.844	1.103	0	0	0	0	0	0	0	4	0	2.142	3.534
289	FQNP355	346.36	146.216	1.134	0	0	0	0	0	0	0	6	0	1.888	3.294
290	FQNP356	262.28	98.32	1.374	0	0	0	0	2	2	0	0	0	1.651	3.610
291	FQNP357	328.39	146.742	1.218	0	0	0	0	0	0	0	0	0	3.051	3.918
292	FQNP358	316.38	136.813	1.26	0	0	0	0	0	0	0	1	0	2.748	3.781
293	FQNP359	372.45	172.733	1.214	0	0	0	0	0	0	0	0	0	3.015	3.934
294	FQNP360	360.44	162.403	1.244	0	0	0	0	0	0	0	1	0	2.718	3.802
295	FQNP361	314.31	169.95	0.96	0	0	1	0	2	1	0	0	0	2.129	4.381
296	FQNP362	314.31	171.951	0.974	0	0	0	0	2	1	0	0	0	2.262	4.046
297	FQNP363	258.29	79.872	1.14	0	0	1	0	1	1	0	2	0	1.76	3.916
298	FQNP364	304.37	156.483	0.715	0	0	0	0	2	1	0	0	0	4.19	4.198
299	FQNP367	256.27	98.673	1.535	0	0	1	0	1	0	0	0	0	1.7	4.167
300	FQNP369	272.32	106.425	1.319	0	0	0	0	2	1	0	0	0	1.7	3.843
301	FQNP371	242.29	92.238	1.545	0	0	0	0	2	0	0	0	0	1.73	3.940
302	FQNP373	208.23	52.932	1.193	0	0	0	0	1	0	0	3	0	3.201	3.678
303	FQNP374	340.4	141.874	1	0	0	1	0	2	0	0	0	0	2.262	4.554
304	FQNP375	334.45	93.422	0.968	0	0	0	0	0	0	0	3	0	4.107	3.707
305	FQNP376	316.43	0	1.28	0	0	0	0	0	0	0	1	0	2.744	3.694
306	FQNP377	330.46	0	1.278	0	0	0	0	0	0	0	2	0	3.18	3.600
307	FQNP378	320.42	88.432	0.969	0	0	0	0	0	0	0	2	0	3.783	3.801
308	FQNP379	230.33	0	2.015	0	0	0	0	0	0	0	0	0	1.342	3.443
309	FQNP380	232.35	0	1.417	0	0	0	0	0	0	0	0	0	1.524	3.709
310	FQNP381	332.48	0	1.335	0	0	0	0	0	0	0	2	0	3.802	3.591
311	FQNP382	318.45	0	1.289	0	0	0	0	0	0	0	1	0	3.335	3.705
312	FQNP384	218.37	57.922	1.333	0	0	0	0	1	0	0	0	0	3.825	3.951
313	FQNP385	384.46	145.069	1.227	0	0	2	0	0	0	0	0	0	3.818	4.599
314	FQNP386	370.43	139.8	1.182	0	0	2	0	0	0	0	0	0	3.791	4.615

315	FQNP387	370.43	137.372	1.229	0	0	2	0	1	1	0	0	0	3.699	4.502
316	FQNP388	414.44	132.901	0.942	0	0	2	0	0	0	0	0	0	3.812	4.717
317	FQNP389	274.29	137.479	0.603	0	0	0	0	3	1	0	0	0	3.627	4.337
318	FQNP390	468.44	395.379	0.508	1	0	0	0	5	3	0	0	0	5.736	4.930
319	FQNP391	210.2	50.733	0.3	0	0	0	0	2	2	0	0	0	1.807	4.057
320	FQNP392	286.3	116.827	0.875	0	0	0	0	1	0	0	0	0	1.9	4.137
321	FQNP393	182.19	42.676	0	0	0	0	0	2	1	0	0	0	1.806	4.387
322	FQNP394	124.15	26	0	0	0	0	0	2	0	0	0	0	0.95	4.560
323	FQNP395	212.32	0	1.167	0	0	0	0	1	0	0	2	0	1.8	3.729
324	FQNP396	228.32	0	1.136	0	0	0	0	2	1	0	2	0	1.688	3.651
325	FQNP397	244.32	0	1.133	0	0	0	0	3	1	0	0	0	1.667	3.978
326	FQNP398	260.41	0	1.078	0	0	0	0	2	0	0	0	0	6.774	4.218
327	FQNP400	300.33	119.85	1.087	0	0	0	0	2	1	0	0	0	1.641	3.951
328	FQNP402	338.43	143.089	1.152	0	0	1	0	1	0	0	0	0	2.288	4.374
329	FQNP403	298.36	163.337	1.283	0	0	0	0	2	2	0	4	0	4.811	3.344
330	FQNP404	232.35	61.406	1.007	0	0	0	0	0	0	0	0	0	3.047	3.962
331	FQNP405	362.46	105.103	1.069	0	0	0	0	2	0	0	0	0	3.626	4.203
332	FQNP406	280.35	76.266	1.086	0	0	0	0	3	2	0	0	0	3.797	3.891
333	FQNP407	262.33	68.43	0.991	0	0	1	0	1	0	0	0	0	3.09	4.422
334	FQNP408	304.37	82.552	0.996	0	0	1	0	0	0	0	3	0	3.596	4.010
335	FQNP409	322.39	90.388	1.04	0	0	0	0	2	2	0	3	0	3.429	3.480
336	FQNP418	152.16	35.181	2.292	0	0	0	0	2	0	0	2	0	2.349	3.385
337	FQNP419	284.28	114.56	1.193	0	0	0	0	1	0	0	0	0	2.034	4.000
338	FQNP424	166.19	39.792	1.467	0	0	0	0	1	0	0	2	0	2.678	3.643
339	FQNP425	248.35	83.447	1.36	0	0	0	0	1	0	0	2	0	2.132	3.701
340	FQNP426	238.41	0	3.4	0	0	0	0	2	0	0	0	0	1.35	3.066
341	FQNP428	366.7	0	0.677	0	0	0	0	0	0	0	2	0	10.179	4.038
342	FQNP429	380.73	0	0.669	0	0	0	0	0	0	0	2	0	10.607	4.052
343	FQNP430	394.76	0	0.661	0	0	0	0	0	0	0	2	0	11.033	4.066

344	FQNP431	408.79	0	0.654	0	0	0	0	0	0	0	2	0	11.458	4.080
345	FQNP432	422.82	0	0.647	0	0	0	0	0	0	0	2	0	11.881	4.093
346	FQNP433	436.85	0	0.641	0	0	0	0	0	0	0	2	0	12.302	4.106
347	FQNP434	450.88	0	0.636	0	0	0	0	0	0	0	2	0	12.722	4.119
348	FQNP435	260.31	66.799	1.425	0	0	0	0	1	1	0	2	0	1.58	3.446
349	FQNP436	170.18	0	0	0	0	1	0	1	0	0	0	0	1.461	4.776
350	FQNP437	288.32	148.231	0.729	0	0	0	0	1	0	0	0	0	4.167	4.276
351	FQNP438	258.29	132.485	0.713	0	0	0	0	1	1	0	0	0	4.167	4.070
352	FQNP439	272.32	140.226	0.699	0	0	0	0	0	0	0	0	0	3.951	4.164
353	FQNP440	288.32	148.955	0.926	0	0	0	0	1	1	0	0	0	4.378	3.992
354	FQNP441	258.29	132.485	0.76	0	0	0	0	1	1	0	0	0	3.972	4.045
355	FQNP442	302.35	156.697	1.112	0	0	0	0	0	0	0	0	0	3.916	3.992
356	FQNP443	288.32	148.955	1.105	0	0	0	0	1	1	0	0	0	3.982	3.903
357	FQNP444	302.35	156.697	0.858	0	0	0	0	0	0	0	0	0	4.388	4.115
358	FQNP448	516.74	210.886	1.114	0	0	0	0	3	0	0	0	0	2.05	4.320
359	FQNP449	574.78	242.922	1.046	0	0	0	0	3	1	0	3	0	2.075	3.849
360	FQNP450	532.74	217.92	1.112	0	0	0	0	4	2	0	0	0	2.037	4.032
361	FQNP451	574.78	239.868	1.106	0	0	0	0	3	1	0	3	0	1.971	3.818
362	FQNP452	516.74	210.886	1.114	0	0	0	0	3	1	0	0	0	2.05	4.116
363	FQNP453	574.78	242.922	1.046	0	0	0	0	3	2	0	3	0	2.075	3.645
364	FQNP454	532.74	217.92	1.112	0	0	0	0	4	3	0	0	0	2.037	3.829
365	FQNP455	574.78	239.868	1.106	0	0	0	0	3	1	0	3	0	1.971	3.818
366	FQNP456	516.74	210.886	1.114	0	0	0	0	3	1	0	0	0	2.05	4.116
367	FQNP457	304.47	67.283	0.76	0	0	0	0	0	0	0	5	0	7.31	3.653
368	FQNP458	332.53	76.826	0.73	0	0	0	0	0	0	0	5	0	8.309	3.696
369	FQNP459	360.59	86.574	0.707	0	0	0	0	0	0	0	5	0	9.279	3.736
370	FQNP460	388.65	96.494	0.687	0	0	0	0	0	0	0	5	0	10.225	3.774
371	FQNP461	330.51	76.826	0.73	0	0	0	0	0	0	0	5	0	8.309	3.696
372	FQNP462	358.57	86.574	0.707	0	0	0	0	0	0	0	5	0	9.279	3.736

373	FQNP463	386.63	96.494	0.687	0	0	0	0	0	0	0	5	0	10.225	3.774
374	FQNP464	314.56	98.865	0.676	0	0	0	0	0	0	0	3	0	8.824	3.956
375	FQNP465	374.62	225.677	1.016	0	0	1	0	0	0	0	2	0	8.77	4.317
376	FQNP466	340.61	98.806	1.043	0	0	1	0	0	0	0	2	0	7.696	4.206
377	FQNP467	340.61	0	0.692	0	0	1	0	0	0	0	2	0	8.237	4.316
378	FQNP468	330.51	76.826	0.73	0	0	0	0	0	0	0	3	0	8.309	3.907
379	FQNP469	358.57	86.574	0.707	0	0	0	0	0	0	0	3	0	9.279	3.946
380	FQNP470	316.58	98.865	0.676	0	0	0	0	0	0	0	5	0	8.824	3.745
381	FQNP473	482.62	219.028	0.89	0	0	1	0	0	0	0	3	0	2.07	4.096
382	FQNP474	222.41	67.335	2.182	0	0	0	0	1	0	0	0	0	1.419	3.525
383	FQNP475	444.66	243.866	1.304	0	0	0	0	2	1	0	0	0	2.436	3.947
384	FQNP476	228.31	75.336	1.943	1	0	0	0	1	0	0	0	0	1.853	4.174
385	FQNP477	246.33	81.527	0.85	0	0	0	0	2	1	0	0	0	1.631	4.033
386	FQNP478	230.28	83.129	0.747	0	0	0	0	0	0	0	0	0	3.493	4.099
387	FQNP479	214.33	68.176	1.6	0	0	0	0	1	0	0	0	0	1.545	3.783
388	FQNP480	218.37	68.176	1.6	0	0	0	0	1	0	0	0	0	1.545	3.783
389	FQNP481	214.38	68.176	0	0	0	0	0	0	0	0	0	0	1.545	4.369
390	FQNP482	250.37	80.415	1.8	0	0	0	0	2	0	0	0	0	1.769	3.823
391	FQNP491	218.37	29.016	0	0	0	0	0	0	0	0	0	0	0.903	4.331
392	FQNP492	220.39	29.016	0	0	0	0	0	1	0	0	0	0	0.903	4.445
393	FQNP493	270.41	35.971	1.14	0	0	0	0	2	1	0	0	0	1.475	3.876
394	FQNP495	546.96	371.127	0.955	0	0	1	0	1	0	0	0	0	2.753	4.603
395	FQNP496	228.26	88.562	1.349	0	0	0	0	0	0	0	0	0	1.789	3.796
396	FQNP497	296.39	131.487	0.921	0	0	0	0	0	0	0	0	0	2.425	4.024
397	FQNP498	254.3	69.628	0.445	0	0	0	0	0	0	0	0	0	2.178	4.191
398	FQNP499	228.26	61.241	0.81	0	0	0	0	0	0	0	0	0	2.036	4.023
399	FQNP500	314.41	93.122	0.694	0	0	0	0	1	1	0	0	0	2.695	4.019
400	FQNP501	228.26	61.241	0.81	0	0	0	0	0	0	0	1	0	2.036	3.917
401	FQNP502	354.38	111.762	1.185	0	0	2	0	0	0	0	0	0	3.672	4.595

402	FQNP503	370.43	139.8	1.182	0	0	2	0	0	0	0	0	0	3.791	4.615
403	FQNP504	220.39	0	2.182	0	0	1	0	0	0	0	0	0	1.439	3.706
404	FQNP505	223.4	0	0.845	0	0	0	0	1	0	0	0	0	5.769	4.180
405	FQNP506	1199.65	1656.598	1.024	0	0	10	0	10	7	0	4	0	8.71	7.644
406	FQNP507	1223.67	1189.333	1.054	0	0	10	0	8	6	0	5	0	5.649	7.156
407	FQNP508	306.54	43.844	0.963	0	0	0	0	2	0	0	0	0	1.648	4.166
408	FQNP509	348.58	54.411	0.866	0	0	0	0	1	0	0	3	0	2.614	3.807
409	FQNP510	272.52	38.593	0	0	0	0	0	0	0	0	0	0	1.514	4.351
410	FQNP511	272.52	38.593	0	0	0	0	0	0	0	0	0	0	1.514	4.351
411	FQNP512	272.52	38.593	0	0	0	0	0	0	0	0	0	0	1.514	4.351
412	FQNP513	230.33	56.674	1.007	0	0	0	0	1	1	0	0	0	1.318	3.827
413	FQNP514	246.33	61.057	0.756	0	0	0	0	1	0	0	0	0	1.247	4.141
414	FQNP517	531.76	75.315	1.165	0	0	0	0	2	0	0	4	0	2.911	3.705
415	FQNP518	517.73	73.202	1.134	0	0	0	0	2	0	0	4	0	2.978	3.719
416	FQNP519	533.73	75.65	1.084	0	0	0	0	3	0	0	3	0	2.96	3.962
417	FQNP520	434.48	59.269	0.884	0	0	1	0	2	0	0	5	0	1.761	4.019
418	FQNP521	474.5	65.611	0.827	0	0	2	0	0	0	0	8	0	2.4	3.851
419	FQNP522	432.46	56.312	0.922	0	0	3	0	0	0	0	5	0	1.702	4.436
420	FQNP523	344.44	0	0.83	0	0	1	0	0	0	0	2	0	1.667	4.093
421	FQNP524	312.44	0	0.862	0	0	0	0	0	0	0	2	0	1.568	3.743
422	FQNP527	178.15	52.369	1.625	0	0	0	0	2	0	0	0	0	1.384	3.874
423	FQNP530	332.28	186.472	0.967	0	0	0	0	5	3	0	0	0	2.648	4.004
424	FQNP531	318.25	175.657	0.892	0	0	0	0	6	4	0	0	0	2.59	3.940
425	FQNP533	204.19	63.096	1.364	0	0	0	0	1	1	0	3	0	1.52	3.363
426	FQNP535	218.22	71.989	1	0	0	0	0	0	0	0	3	0	1.659	3.620
427	FQNP536	560.9	237.694	1.147	0	0	0	0	2	0	0	7	0	4.453	3.528
428	FQNP537	574.78	244.195	1.183	0	0	0	0	4	0	0	5	0	4.401	3.955
429	FQNP538	652.91	412.194	0.956	0	0	2	0	7	3	0	5	0	4.444	4.552
430	FQNP539	460.47	352.603	0.934	0	0	1	0	6	3	0	2	0	3.689	4.377

431	FQNP540	460.47	352.603	0.934	0	0	1	0	6	3	0	2	0	3.689	4.377
432	FQNP541	446.44	339.588	0.966	0	0	1	0	7	3	0	2	0	3.797	4.473
433	FQNP542	512.56	409.063	0.805	0	0	3	0	6	2	1	2	1	4.621	6.430
434	FQNP543	290.34	80.673	1.074	0	0	0	0	1	0	0	0	0	2.018	4.032
435	FQNP544	372.45	122.65	1.035	0	0	0	0	0	0	0	0	0	3.639	3.999
436	FQNP546	302.5	0	0.78	0	0	1	0	0	0	0	2	0	1.754	4.117
437	FQNP547	302.5	0	0.78	0	0	1	0	0	0	0	2	0	1.754	4.117
438	FQNP548	446.49	61.383	0.86	0	0	2	0	0	0	0	5	0	1.996	4.140
439	FQNP549	434.48	59.269	0.884	0	0	1	0	2	1	0	5	0	1.761	3.815
440	FQNP550	328.44	0	0.883	0	0	0	0	0	0	0	2	0	1.689	3.736
441	FQNP551	416.46	54.202	0.858	0	0	2	0	0	0	0	5	0	1.756	4.131
442	FQNP552	279.49	87.419	2.021	0	0	0	0	1	0	0	0	0	1.885	3.618
443	FQNP553	295.49	93.959	1.729	0	0	0	0	1	0	0	0	0	1.908	3.751
444	FQNP554	302.36	106.192	1.753	0	0	5	0	2	1	0	0	0	1.027	5.304
445	FQNP556	302.35	172.423	0.988	0	0	0	0	2	1	0	0	0	2.838	4.054
446	FQNP557	352.41	136.173	1.004	0	0	0	0	2	0	0	0	0	2.638	4.225
447	FQNP558	272.32	152.916	1.048	0	0	0	0	2	0	0	0	0	2.826	4.220
448	FQNP559	300.33	106.57	0.776	0	0	0	0	1	1	0	0	0	2.186	3.978
449	FQNP560	316.33	112.887	0.827	0	0	0	0	2	2	0	0	0	2.204	3.871
450	FQNP561	240.27	127.648	0.85	0	0	0	0	1	0	0	2	0	2.349	3.955
451	FQNP562	442.8	223.21	1.323	0	0	0	0	1	0	0	1	0	2.371	3.908
452	FQNP563	442.8	232.849	1.033	0	0	0	0	1	0	0	3	0	2.194	3.826
453	FQNP564	458.8	244.695	1.035	0	0	0	0	2	0	0	1	0	2.333	4.161
454	FQNP565	498.82	282.541	0.984	0	0	0	0	1	0	0	3	0	2.668	3.887
455	FQNP566	208.28	51.057	1.094	0	0	0	0	0	0	0	0	0	1.788	3.886
456	FQNP567	418.48	183.245	1.236	0	0	2	0	2	2	0	0	0	3.955	4.443
457	FQNP568	226.25	54.924	1.19	0	0	0	0	2	2	0	2	0	1.791	3.458
458	FQNP569	182.19	43.659	1.3	0	0	0	0	1	0	0	2	0	2.495	3.714
459	FQNP571	1369.82	1718.197	1.079	0	0	10	0	8	6	0	5	0	6.149	7.460

FQNP572	1383.85	1778.609	1.097	0	0	10	0	8	6	0	6	0	6.324	7.386
FQNP573	2795.76	9251.423	1.099	0	0	20	0	16	8	0	10	0	22.254	15.481
FQNP574	320.52	81.901	1.23	0	0	1	0	2	0	0	0	0	1.688	4.405
FQNP576	304.52	76.911	0.912	0	0	1	0	1	0	0	0	0	1.641	4.425
FQNP579	217.24	49.06	1.132	0	0	1	0	1	0	0	0	0	2.602	4.337
FQNP580	264.35	69.14	0.6	0	0	0	0	2	0	0	2	0	2.85	4.158
FQNP581	447.58	280.529	1.024	0	0	0	0	2	0	0	0	0	2.394	4.293
FQNP582	645.69	673.098	0.995	0	0	5	0	14	12	0	0	0	6.025	5.223
FQNP583	207.26	46.543	0.701	0	0	0	0	5	2	0	0	0	1.666	4.219
FQNP584	344.54	114.451	1.364	0	0	0	0	0	0	0	5	0	1.685	3.275
FQNP585	330.56	134.288	1.613	0	0	0	0	0	0	0	3	0	1.687	3.388
FQNP586	330.56	134.288	1.613	0	0	0	0	0	0	0	3	0	1.687	3.388
FQNP587	274.29	102.014	0.447	0	0	2	0	0	0	0	1	0	1.152	4.745
FQNP590	306.54	109.544	0.502	0	0	0	0	1	0	0	2	0	3.642	4.129
FQNP591	320.57	117.416	0.502	0	0	0	0	0	0	0	2	0	4.025	4.028
FQNP592	324.56	115.464	1.054	0	0	0	0	2	0	0	2	0	3.524	4.003
FQNP593	216.25	69.552	1.026	0	0	0	0	1	1	0	3	0	1.738	3.521
FQNP594	544.64	455.734	1.102	0	0	2	0	2	2	0	2	0	2.977	4.423
FQNP595	570.63	485.217	1.065	0	0	2	0	2	1	0	0	0	3.108	4.873
FQNP596	544.64	460.628	1.061	0	0	2	0	3	2	0	2	0	3.187	4.563
FQNP597	526.62	443.139	1.116	0	0	2	0	2	1	0	0	0	3.087	4.826
FQNP598	540.6	455.132	1.051	0	0	2	0	2	1	0	0	0	3.101	4.862
FQNP599	194.24	71.54	1.875	0	0	0	0	0	0	0	2	0	1.495	3.338
FQNP600	318.34	203.129	0.493	0	0	0	0	1	1	0	2	0	1.968	3.941
FQNP601	334.34	213.855	0.677	0	0	0	0	2	2	0	2	0	1.982	3.779
FQNP602	392.68	158.183	0.784	0	0	0	0	0	0	0	2	0	3.831	3.923
FQNP603	348.53	147.179	1.224	0	0	0	0	3	1	0	0	0	2.7	4.048
FQNP604	330.51	141.617	1.314	0	0	0	0	2	0	0	0	0	2.733	4.095
FQNP605	330.51	139.883	1.209	0	0	0	0	2	0	0	0	0	2.708	4.139

489	FQNP607	330.51	139.883	1.209	0	0	0	0	2	0	0	0	0	2.708	4.139
490	FQNP608	330.51	129.87	1.209	0	0	1	0	1	0	0	0	0	2.707	4.352
491	FQNP609	416.56	193.082	1.11	1	0	0	0	2	1	0	3	0	2.964	4.229
492	FQNP610	348.53	147.179	1.224	0	0	0	0	3	1	0	0	0	2.7	4.048
493	FQNP612	380.52	161.315	1.284	0	0	0	0	0	0	0	0	0	2.461	3.883
494	FQNP613	412.77	167.372	0.769	0	0	0	0	1	0	0	0	0	4.08	4.267
495	FQNP614	180.18	37.6	0	0	0	0	0	6	5	0	0	0	0.873	4.006
496	FQNP615	252.44	34.261	1.594	0	0	0	0	2	0	0	0	0	1.213	3.873
497	FQNP616	304.46	186.229	0.885	0	0	0	0	0	0	0	0	0	3.652	4.102
498	FQNP617	218.37	68.176	1.6	0	0	0	0	1	0	0	0	0	1.545	3.783
499	FQNP618	316.33	180.798	0.827	0	0	0	0	2	2	0	2	0	2.918	3.717
500	FQNP620	456.48	197.256	1.258	0	0	0	0	0	0	0	4	0	2.603	3.497
501	FQNP621	382.39	132.831	1.259	0	0	0	0	0	0	0	1	0	2.382	3.770
502	FQNP622	348.44	163.557	0.904	0	0	2	0	6	2	0	0	0	3.322	5.021
503	FQNP623	332.44	155.904	0.935	0	0	2	0	5	1	0	0	0	3.309	5.091
504	FQNP624	186.28	42.114	1.3	0	0	0	0	3	1	0	0	0	1.566	3.926
505	FQNP625	186.28	42.114	1.3	0	0	0	0	3	1	0	0	0	1.566	3.926
506	FQNP626	300.28	168.586	0.942	0	0	0	0	3	0	0	0	0	2.82	4.390
507	FQNP627	290.27	148.117	0.683	1	0	0	0	3	0	0	0	1	3.723	4.857
508	FQNP628	420.49	331.777	1.409	0	0	0	0	2	1	0	1	0	3.432	3.870
509	FQNP629	420.49	326.113	1.442	0	0	0	0	2	1	0	1	0	3.367	3.851
510	FQNP630	406.51	265.803	1.182	0	0	0	0	2	1	0	2	0	2.604	3.806
511	FQNP631	422.51	295.134	1.319	0	0	0	0	3	1	0	1	0	3.672	4.010
512	FQNP632	422.51	275.107	1.196	0	0	0	0	3	2	0	0	0	2.537	3.926
513	FQNP636	472.78	203.683	1.109	0	0	0	0	3	0	0	0	0	2.381	4.326
514	FQNP637	486.76	212.614	1.133	0	0	0	0	3	0	0	0	0	2.467	4.323
515	FQNP638	512.85	257.276	1.07	0	0	2	0	1	0	0	0	0	2.622	4.817
516	FQNP639	428.5	195.18	0.712	0	1	0	0	2	0	0	2	0	2.342	5.417
517	FQNP640	547.62	417.868	1.231	0	0	0	0	1	0	0	0	0	5.489	4.243

518	FQNP641	293.29	80.316	1.304	0	0	0	0	1	0	0	0	0	1.492	3.918
519	FQNP642	339.37	116.861	1.016	0	0	0	0	1	0	0	0	0	1.446	4.064
520	FQNP643	256.43	0	1.545	0	0	0	0	3	0	0	0	0	1.48	3.996
521	FQNP644	442.85	178.252	0.744	0	0	0	0	1	0	0	0	0	4.082	4.284
522	FQNP645	426.8	221.611	0.674	0	0	0	0	1	0	0	0	0	2.212	4.293
523	FQNP646	456.78	245.124	1.235	0	0	0	0	2	0	0	0	0	2.301	4.178
524	FQNP647	456.78	243.519	1.086	0	0	0	0	2	0	0	0	0	2.275	4.242
525	FQNP648	442.81	0	0.662	0	0	0	0	2	1	0	2	0	12.188	4.120
526	FQNP651	194.21	41.79	1.857	0	0	1	0	5	5	0	0	0	1.173	3.421
527	FQNP652	434.43	362.775	0.791	0	0	1	0	6	3	0	2	0	4.198	4.458
528	FQNP653	342.34	80.959	0.958	0	0	3	0	8	6	0	0	0	3.136	4.694

Table S7. List of 60-derivatives, their calculated molecular descriptors, and predicted pMIC values

MolID	MW	D/Dr06	GATS6m	nArCOOH	nRCONH2	nROR	nHDon	nHBonds	H051	TI2	*p*MIC
60	334.46	90.65	0.904	0	0	2	4	3	2	4.886	4.374
60a	333.48	90.65	0.922	0	1	2	5	3	2	4.886	5.730
60b	454.57	266.815	0.842	1	0	2	4	3	2	7.848	5.105
60c	334.51	90.71	0.893	0	0	3	3	3	0	4.958	4.810
60d	376.55	103.221	0.867	0	0	5	1	0	2	4.848	5.664
60e	390.58	108.402	0.862	0	0	5	0	0	2	5.284	5.565

References

1. Gonzalez, M.; Zamilpa, A.; Marquina, S.; Navarro, V.; Alvarez, L., Antimycotic spirostanol saponins from Solanum hispidum leaves and their structure-activity relationships. *J Nat Prod* **2004,** *67* (6), 938-41.

2. Pettit, G. R.; Schaufelberger, D. E.; Nieman, R. A.; Dufresne, C.; Saenz-Renauld, J. A., Antineoplastic agents, 177. Isolation and structure of phyllanthostatin 6. *J Nat Prod* **1990,** *53* (6), 1406-13.

3. Wang, T. M.; Hojo, T.; Ran, F. X.; Wang, R. F.; Wang, R. Q.; Chen, H. B.; Cui, J. R.; Shang, M. Y.; Cai, S. Q., Cardenolides from Saussurea stella with cytotoxicity toward cancer cells. *J Nat Prod* **2007,** *70* (9), 1429-33.

4. Tezuka, Y.; Honda, K.; Banskota, A. H.; Thet, M. M.; Kadota, S., Kinmoonosides A-C, three new cytotoxic saponins from the fruits of Acacia concinna, a medicinal plant collected in myanmar. *J Nat Prod* **2000,** *63* (12), 1658-64.

5. Susplugas, S.; Hung, N. V.; Bignon, J.; Thoison, O.; Kruczynski, A.; Sevenet, T.; Gueritte, F., Cytotoxic arylnaphthalene lignans from a Vietnamese acanthaceae, Justicia patentiflora. *J Nat Prod* **2005,** *68* (5), 734-8.

6. Zhang, H. J.; Rumschlag-Booms, E.; Guan, Y. F.; Wang, D. Y.; Liu, K. L.; Li, W. F.; Nguyen, V. H.; Cuong, N. M.; Soejarto, D. D.; Fong, H. H. S.; Rong, L., Potent Inhibitor of Drug-Resistant HIV-1 Strains Identified from the Medicinal Plant Justicia gendarussa. *J Nat Prod* **2017,** *80* (6), 1798-1807.

7. Gelmi, M. L.; Fontana, G.; Pocar, D.; Pontremoli, G.; Pellegrino, S.; Bombardelli, E.; Riva, A.; Balduini, W.; Carloni, S.; Cimino, M., Novel 3-O-glycosyl-3-demethylthiocolchicines as ligands for glycine and gamma-aminobutyric acid receptors. *J Med Chem* **2007,** *50* (9), 2245-8.

8. Singh, A.; Kumar, N.; Singh, S.; Sewariya, S.; Sharma, M. K.; Chandra, R., High-valued pyrazinoindole analogues: Synthesis, antibacterial activity, structure activity relationship and molecular dynamics analyses. *Results in Chemistry* **2021,** *3*, 100194.

9. Wang, J.; Zhang, P. L.; Ansari, M. F.; Li, S.; Zhou, C. H., Molecular design and preparation of 2-aminothiazole sulfanilamide oximes as membrane active antibacterial agents for drug resistant Acinetobacter baumannii. *Bioorg Chem* **2021,** *113*, 105039.

10. Vereshchagin, A. N.; Frolov, N. A.; Konyuhova, V. Y.; Kapelistaya, E. A.; Hansford, K. A.; Egorov, M. P., Investigations into the structure-activity relationship in gemini QACs based on biphenyl and oxydiphenyl linker. *RSC Adv* **2021,** *11* (6), 3429-3438.

11. Zhou, M.; Eun, Y. J.; Guzei, I. A.; Weibel, D. B., Structure-activity studies of divin: an inhibitor of bacterial cell division. *ACS Med Chem Lett* **2013,** *4* (9), 880-885.

12. Matsingos, C.; Al-Adhami, T.; Jamshidi, S.; Hind, C.; Clifford, M.; Mark Sutton, J.; Rahman, K. M., Synthesis, microbiological evaluation and structure activity relationship analysis of linezolid analogues with different C5-acylamino substituents. *Bioorg Med Chem* **2021,** *49*, 116397.

13. Lyons, A.; Kirkham, J.; Blades, K.; Orr, D.; Dauncey, E.; Smith, O.; Dick, E.; Walker, R.; Matthews, T.; Bunt, A.; Finlayson, J.; Morrison, I.; Savage, V. J.; Moyo, E.; Butler, H. S.; Newman, R.; Ooi, N.; Smith, A.; Charrier, C.; Ratcliffe, A. J.; Stokes, N. R.; Best, S.; Salisbury, A. M.; Craighead, M.; Cooper, I. R., Discovery and structure-activity relationships of a novel oxazolidinone class of bacterial type II topoisomerase inhibitors. *Bioorg Med Chem Lett* **2022,** *65*, 128648.

14. Pilon-Jimenez, B. A.; Saldivar-Gonzalez, F. I.; Diaz-Eufracio, B. I.; Medina-Franco, J. L., BIOFACQUIM: A Mexican Compound Database of Natural Products. *Biomolecules* **2019,** *9* (1), 31.
